# Selective Manganese-Catalyzed Dimerization and Cross-Coupling of Terminal Alkynes

**DOI:** 10.1021/acscatal.1c01137

**Published:** 2021-05-18

**Authors:** Stefan Weber, Luis F. Veiros, Karl Kirchner

**Affiliations:** † Institute of Applied Synthetic Chemistry, Vienna University of Technology, Getreidemarkt 9, A-1060 Vienna, Austria; ‡ Centro de Química Estrutural and Departamento de Engenharia Química, Instituto Superior Técnico, Universidade de Lisboa, Av Rovisco Pais, 1049-001 Lisboa, Portugal

**Keywords:** manganese, alkyl complex, terminal alkynes, 1,3-enynes, bisphosphine, DFT calculations, cross-coupling

## Abstract

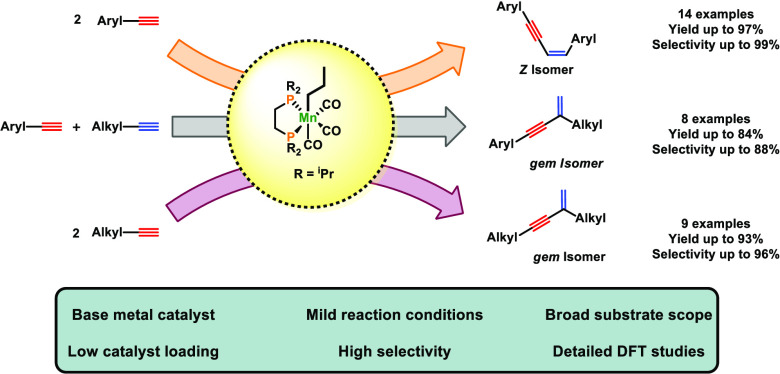

Herein, efficient manganese-catalyzed dimerization of terminal alkynes to afford 1,3-enynes is described. This reaction is atom economic, implementing an inexpensive, earth-abundant nonprecious metal catalyst. The precatalyst is the bench-stable alkyl bisphosphine Mn(I) complex *fac-*[Mn(dippe)(CO)_3_(CH_2_CH_2_CH_3_)]. The catalytic process is initiated by migratory insertion of a CO ligand into the Mn–alkyl bond to yield an acyl intermediate that undergoes rapid C–H bond cleavage of alkyne, forming an active Mn(I) acetylide catalyst [Mn(dippe)(CO)_2_(C≡CPh)(η^2^-HC≡CPh)] together with liberated butanal. A range of aromatic and aliphatic terminal alkynes were efficiently and selectively converted into head-to-head *Z*-1,3-enynes and head-to-tail *gem*-1,3-enynes, respectively, in good to excellent yields. Moreover, cross-coupling of aromatic and aliphatic alkynes selectively yields head-to-tail *gem*-1,3-enynes. In all cases, the reactions were performed at 70 °C with a catalyst loading of 1–2 mol %. A mechanism based on density functional theory (DFT) calculations is presented.

## Introduction

**Scheme 1:**
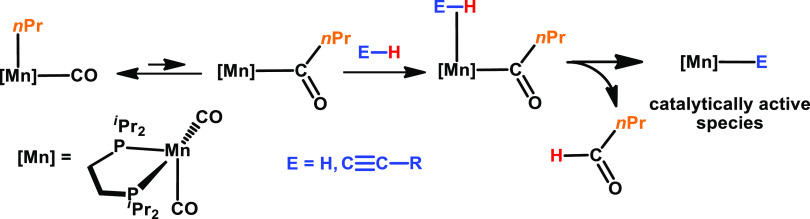
Formation of Mn(I) Hydride and Acetylide Species by Alkyl Migration Followed by Aldehyde Release upon E–H Bond Cleavage

CO ligands in Mn(I) alkyl carbonyl complexes are known to undergo migratory insertions to form highly reactive coordinatively unsaturated acyl complexes, which can be trapped in the presence of strong field ligands such as CO or tertiary phosphines—a well-known textbook reaction.^1^ The classic reaction is the formation of Mn(CO)_5_(η^1^-COCH_3_) from Mn(CO)_5_(CH_3_) in the presence of CO.^2−4^ On the other hand, these acyl 16e^–^ intermediates can be utilized to activate dihydrogen, possibly also molecules featuring weakly polar E–H (E = e.g., C, Si, B) bonds. For instance, dihydrogen is able to react with transition metal–acyl complexes to afford aldehydes and metal hydride complexes. This process is accompanied by H–H and metal−σ–C bond cleavage and is typically the final step in both stoichiometric and catalytic hydroformylations of alkenes (Scheme 1).^5,6^

We have recently described the hydrogenation of alkenes and nitriles utilizing Mn(I) complexes *fac-*[Mn(dpre)(CO)_3_(R)] (dpre = 1,2-bis(di-*n*-propylphosphino) ethane, R = CH_3_, CH_2_CH_3_, CH_2_CH_2_CH_3_) and *fac-*[Mn(dippe)(CO)_3_(CH_2_CH_2_CH_3_) (dippe = 1,2-bis(di-*iso-*propylphosphino)ethane) where we took the advantage of the migratory insertion and hydrogenolysis processes to create the active 16e^–^ Mn(I) hydride catalysts (Scheme 1).^7^

Here, we describe the activity of *fac*-[Mn(dippe)(CO)_3_(CH_2_CH_2_CH_3_)] (**1**) as a precatalyst for the dimerization of terminal aromatic and aliphatic alkynes to afford selectively head-to-head *Z*-1,3-enynes and head-to-tail *gem*-1,3-enynes, respectively. The initiation step involves C–H bond activation of the terminal alkyne forming an active Mn(I) acetylide catalyst together with free butanal (Scheme 1). This is the first example of manganese-catalyzed dimerization and cross-coupling of terminal alkynes,^8,9^ which proceeds without any additives under mild conditions.

## Results and Discussion

The catalytic performance of **1** was first investigated for the dimerization of phenylacetylene as a model substrate. Selected optimization experiments are depicted in Table 1. With **1** as a precatalyst and a catalyst loading of 2 mol %, quantitative formation of 1,3-enyne was observed at 70 °C (Table 1, entry 1). Lower reactivities and selectivities were observed where the steric demand of the phosphine donor was reduced by the replacement of *^i^*Pr to *^n^*Pr groups. The use of phenyl groups resulted in negligible reactivity and poor isomer ratio distribution.

**Table 1:**
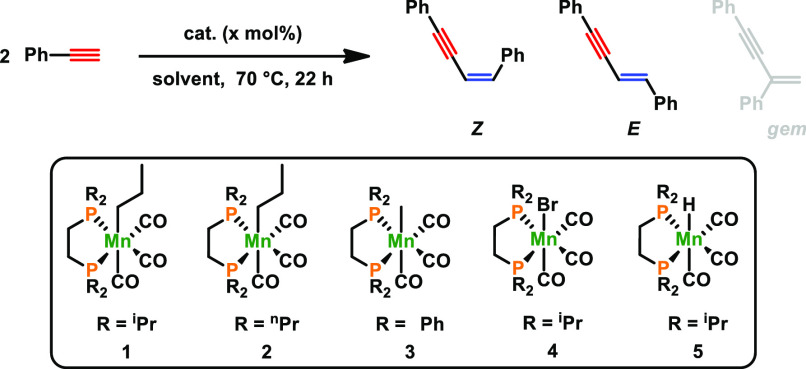
Optimization Reaction for the Dimerization of Phenylacetylene^a^

entry	catalyst (mol %)	solvent	conversion [%]	*Z:E* ratio
1	**1** (2)	THF	>99	96:4
2	**2** (2)	THF	67	79:21
3	**3** (2)	THF	12	73:27
4	**4** (2)	THF	−	n.d.
5	**5** (2)	THF	−	n.d.
6	**1** (2)	toluene	17	95:5
7	**1** (2)	CHCl_3_	12	97:3
**8**^b^	**1 (1)**	**THF**	**>99**	**97:3**
9^b^	**1** (0.5)	THF	82	96:4
10^b^	**1** (0.1)	THF	26	91:9
11^b^^,^^c^	**1** (1)	THF	traces	n.d.
12^b^^,^^e^	**1** (1)	THF	traces	n.d.
13^b^^,^^d^	**1** (1)	THF	94	95:5

a Reaction conditions: phenylacetylene (1.1 mmol), 0.5 mL of anhydrous solvent, 70 °C, Ar, 22 h, conversion and the isomer ratio are determined by gas chromatography–mass spectrometry (GC–MS).

b 18 h.

c 25 °C.

d In air.

e In a 4 M aqueous tetrahydrofuran (THF) solution.

Complexes **2** and **3** exhibited poor reactivities (Table 1, entries 2 and 3), while no reaction took place with complexes **4** and **5** (Table 1 entries 4 and 5), emphasizing the crucial role of the alkyl substituents at the phosphorus donors. It should be noted that high selectivity toward the formation of the *Z*-isomer (>95%) is attributed to the use of **1** as a catalyst, where only small amounts of the *E*-isomer (<5%) and no formation of the geminal isomer were detected. Upon optimization reactions (for details see the Supporting Information (SI)), the catalyst loading could be decreased to 1 mol % (Table 1, entry 8). Lower catalyst loadings of **1** from 0.5 to 0.1 mol % resulted in a significant drop in yields (Table 1, entries 9 and 10). Moreover, only traces of the product could be detected at room temperature (Table 1, entry 11). In the presence of oxygen, the reaction is completely inhibited (Table 1, entry 12), while water is well tolerated. In fact, when the reaction was carried out in a 4 M aqueous solution of THF, 94% conversion was observed (Table 1, entry 13).

Having established the optimized reaction conditions, a broad variety of different aromatic substrates were investigated. Excellent yields could be achieved for substrates containing a halide or an electron-withdrawing group in the para- or ortho-position (Table 2, **7**–**9**, **14**, and **15**). Slightly lower yields were achieved for aryl substrates, containing electron-donating groups such as alkyl or methoxy groups (Table 2, **10**–**13**). Amine functionalities were tolerated (Table 2, **16, 17**, and **19**); however, the reactivity of the system decreased. Good yield could be achieved with 2-ethynylthiophene as a substrate (Table 2, **18**). No conversion was observed for 2-ethynylpyridine and 2-ethynylaniline, presumably due to the coordination of the nitrogen donor blocking the vacant coordination site of the active Mn(I) acetylide catalyst.

**Table 2:**
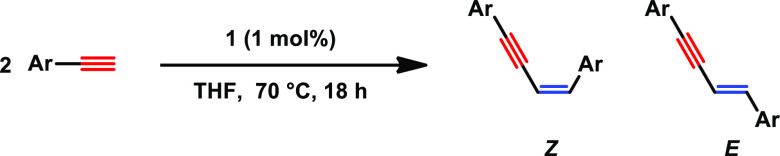

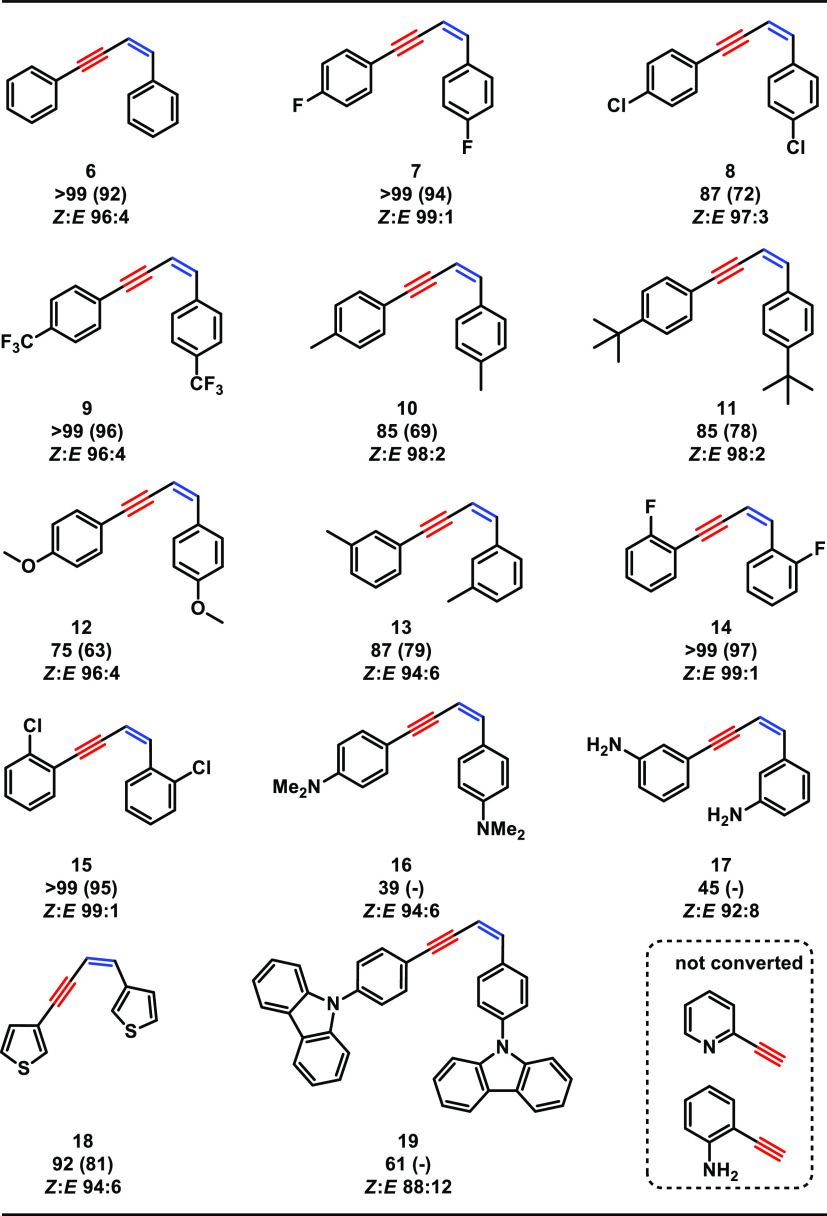
Scope and Limitation of the Dimerization of Aromatic Alkynes Catalyzed by **1**^a^

a Reaction conditions: alkyne (1.1 mmol), **1** (1 mol %), 0.5 mL of anhydrous THF, 70 °C, Ar, 18 h, conversion and the isomer ratio are determined by GC–MS, and isolated yield is given in parenthesis.

The investigation of aliphatic alkynes unveiled an unexpected observation. Apart from the lower reactivity of aliphatic systems, which led to higher catalyst loadings and prolonged reaction times, the product changed drastically. Instead of head-to-head dimerization, massive formation of a head-to-tail product, small amounts of *Z*-1,3-enyne, and no *E*-1,3-enyne were detected. Excellent yields could be achieved for linear aliphatic substrates such as 1-hexyne or 1-octyne (Table 3, **20** and **21**). Lower reactivity was observed for 3-phenyl-1-propyne (Table 3, **24**). Interestingly, a ratio of 39:61 *gem*/*Z* was observed for trimethylsilylacetylene (Table 3, **25**). Cyclic aliphatic systems (Table 3, **26**–**28**) gave excellent yields with high selectivity toward the geminal isomer.

**Table 3:**
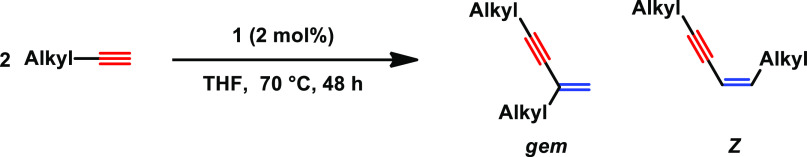

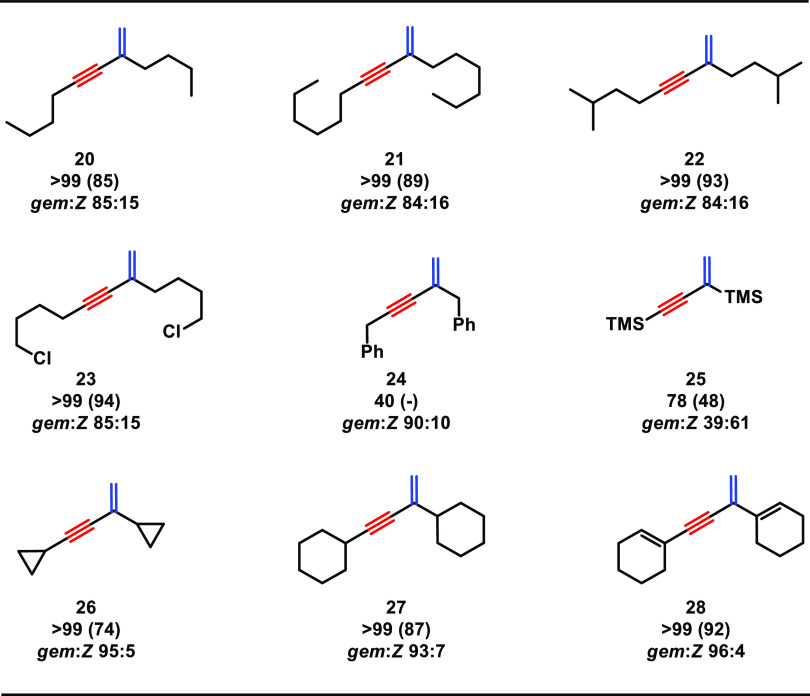
Scope and Limitation of the Dimerization of Aliphatic Alkynes Catalyzed by **1**^a^

a Reaction conditions: alkyne (1.1 mmol), **1** (2 mol %), 0.5 mL of anhydrous THF, 70 °C, Ar, 48 h, conversion and the isomer ratio are determined by GC–MS, and isolated yield is given in parenthesis.

Encouraged by these findings, we aimed for the cross-coupling of aromatic alkynes with aliphatic alkynes, yielding geminal 1,3-enynes. The cross-coupling of alkynes is a challenging field due to the high number of possible cross- and homo-coupling products. The results are represented in Table 4. A detailed table of all detected isomers is provided in the SI. We were able to couple a variety of aromatic substrates with cyclopropylacetylene (Table 4, **29**–**32**). The highest yields were achieved if electron-donating groups, such as *t*Bu or OMe, are present in the para-position. These substrates show a lower tendency to dimerize under the given reaction conditions. Phenylacetylene and 1-chloro-4-phenylacetylene showed a higher amount of dimerization for the investigated systems, which lowers the amount of the cross-coupling product. Good to excellent yields were achieved for the coupling of 4-ethynylanisole with 6-chloro-1-hexyne as well as 1-ethynylcyclohexene. Furthermore, **1** is suitable for the cross-coupling of two aliphatic substrates (cyclopropylacetylene and 1-ethynylcyclohexene, Table 4, **36**). The practical use of the cross-coupling procedure was demonstrated upon upscaling the reaction by a factor of 10, yielding 1.36 g (55%) of **31**.

**Table 4:**
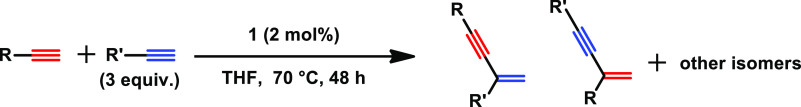

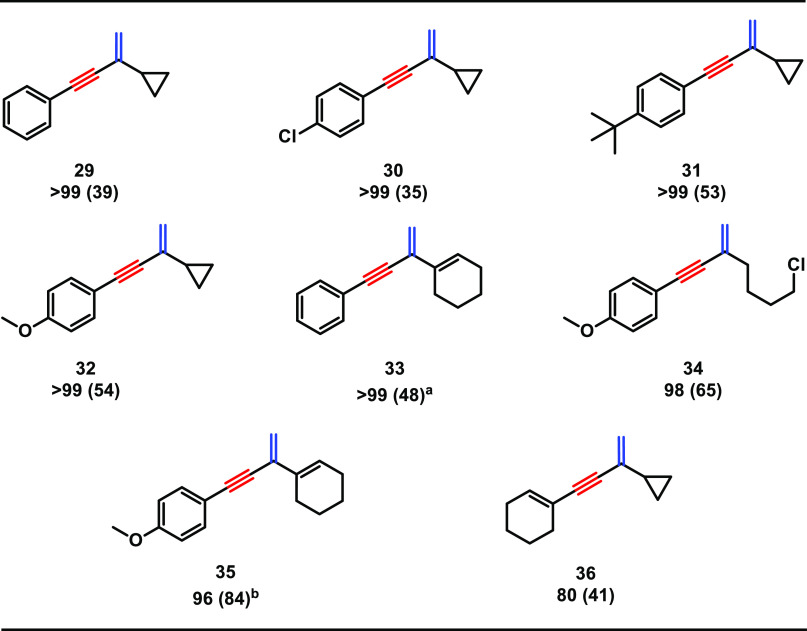
Scope and Limitation of the Cross-Coupling of Alkynes Catalyzed by **1**^a^

a Reaction conditions: alkyne 1 (1.1 mmol, 1 equiv), alkyne 2 (3.3 mmol, 3 equiv), **1** (2 mol %), 0.5 mL of anhydrous THF, 70 °C, Ar, 48 h, conversion of alkyne 1 is determined by GC–MS, isolated yield is given in parenthesis.

b The yield is determined by GC–MS using hexadecane as a standard.

**Scheme 2:**
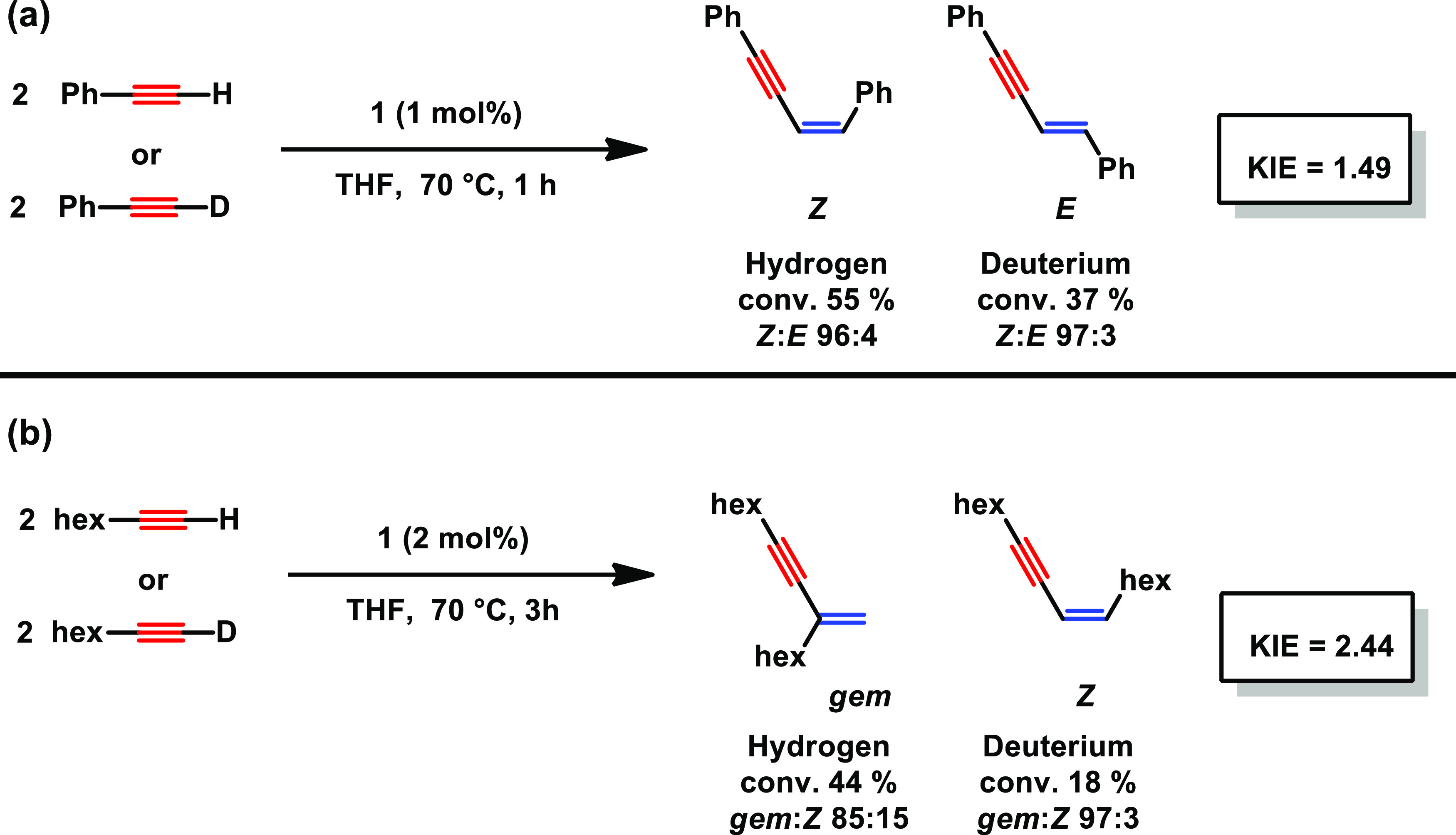
Determination of the KIE for the Dimerization of Phenylacetylene (a) and 1-Octyne (b) Catalyzed by **1**

The homogeneity of the system was confirmed upon the addition of one drop of mercury, whereas no decrease of reactivity and selectivity was observed for the dimerization of phenylacetylene. In the presence of 1 equiv of PMe_3_ (with respect to the substrate), only traces of product formation could be detected, which indicates an inner-sphere mechanism, due to the coordination of PMe_3_ at a vacant site of the active species.^10^ Kinetic isotope effects of 1.49 and 2.44 were detected for the dimerization of phenylacetylene versus phenylacetylene-*d*_1_ and 1-octyne versus 1-octyne-*d*_1_, respectively, as depicted in Scheme 2.

This suggests that the activation of the C–H bond is the rate-determining step during the reaction. These findings are in line with theoretical calculations since the C–H activation upon product release shows the highest energy barrier in the catalytic reaction (*vide infra*). Interestingly, the ratio of *gem*/*Z* for 1-octyne-*d*_1_ has drastically increased to 97:3 (85:17 for 1-octyne). Moreover, a 1,2-aryl shift among the C≡C bond could be ruled out by a ^13^C-labeling experiment with Ph–C≡^13^C–H as a substrate (for details, see the SI).

To get more insights into the dimerization of terminal alkynes, we performed DFT calculations^11^ based on *fac*-[Mn(dippe)(CO)_3_(CH_2_CH_2_CH_3_)] (**1**) as the precatalyst and employing phenylacetylene and cyclopropylacetylene as substrates.

**Scheme 3:**
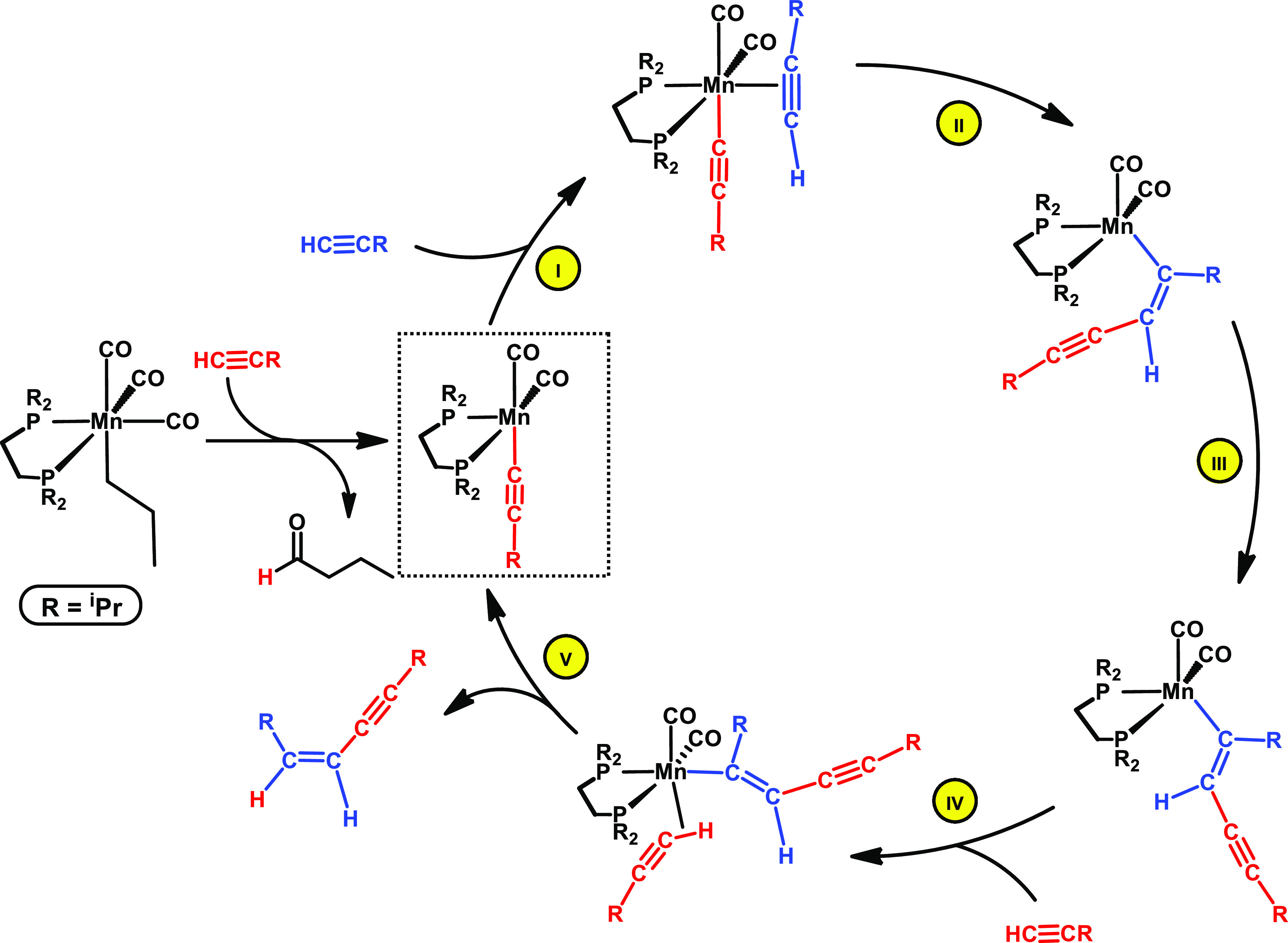
Simplified Catalytic Cycle for the (*Z*)-Selective Dimerization of Terminal Alkynes

Scheme 3 depicts a summary of the catalytic cycle. Our calculations unveiled an acetylene-vinyl mechanism, starting with a head-to-head coupling between the acetylide ligand and the newly coordinated acetylene molecule, yielding a vinyl intermediate (**II**). This intermediate suffers an *E*–*Z* isomerization (**III**) and then is protonated to the final product by a new acetylene molecule (**IV** and **V**), closing the cycle.

**Figure 1:**
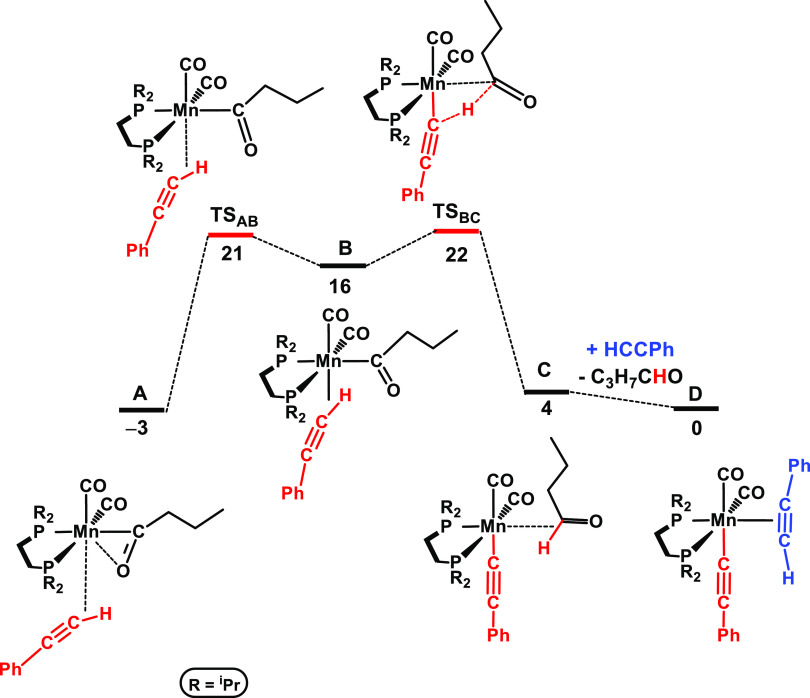
Free energy profile calculated for the formation of the active acetylide catalyst. Free energies (kcal/mol) are referred to [Mn(dippe)(CO)_2_(C≡CPh)(η^2^-*C,H*–HC≡CPh)] (**D** in the calculations).

The free energy profile calculated for the formation of the active species, previous to the initiation of the catalytic cycle, is represented in Figure 1.

Catalyst initiation, starting from **1**, has been reported previously.^7b^ We demonstrated that *n*-butanol is liberated during the catalytic reaction (*n*-butanal is hydrogenated under the reaction conditions) as detected by ^1^H and ^13^C{^1^H} NMR spectroscopy. The first step following the formation of an acyl intermediate is the coordination of one phenylacetylene molecule, from **A** to **B**, and then there is H-transfer from the newly bonded acetylene ligand to the carbonyl C atom, with the formation of aldehyde, from **B** to **C**. This part of the path includes the highest barrier in the profile, Δ*G*^‡^ = 25 kcal/mol, from **A** to **TS**_**BC**_.

From **C**, butanal is liberated and exchanges with a second substrate molecule that coordinates to the metal in a η^2^ mode, in **D**, in an exergonic step (Δ*G* = −4 kcal/mol). **D** is the initial active species in the catalytic cycle.

**Figure 2:**
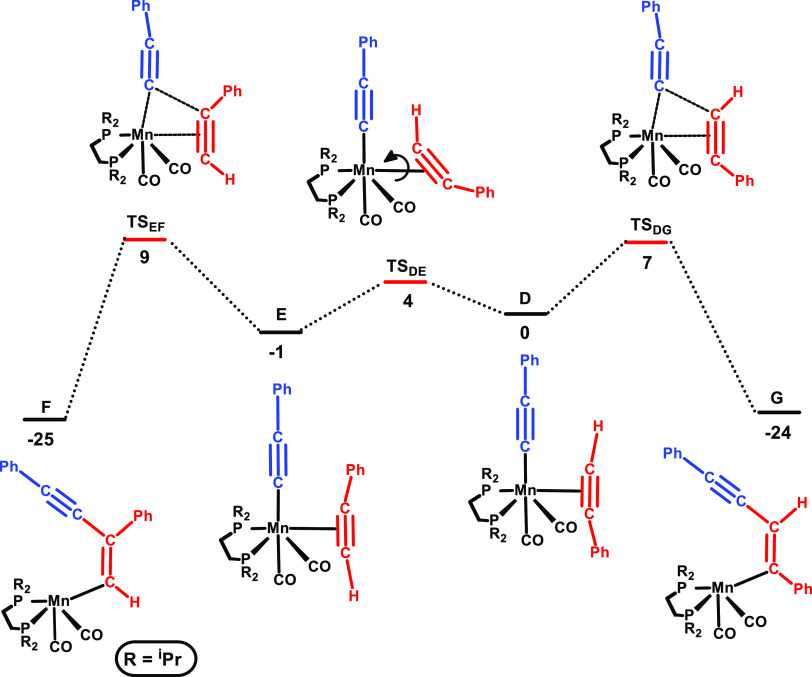
Free energy profile calculated for the regioselectivity of aromatic alkynes. Left side: head-to-tail coupling and right side: head-to-head coupling. Free energies (kcal/mol) are referred to [Mn(dippe)(CO)_2_(C≡CPh)(η^2^-HC≡CPh)] (**D** in the calculations).

The free energy profile represented in Figure 2 addresses the regioselectivity observed for the reaction of phenylacetylene. Starting from the last species in the profile of Figure 1, acetylene η^2^ complex **D**, the relative orientation of the two ligands is the right one for head-to-head coupling and formation of internal *E*-vinyl (**G**). This process has a barrier of 7 kcal/mol (**TS**_**DG**_) and is exergonic with Δ*G* = −24 kcal/mol. This corresponds to step **II** in the catalytic cycle given in Scheme 3.

Alternatively, the rotation of η^2^-acetylene in **D** is an easy process with a barrier of 4 kcal/mol (**TS**_**DE**_) and results in **E**. Here, the orientation of acetylene leads to head-to-tail coupling with the adjacent acetylide and the formation of the terminal vinyl species (**F**). This process is less favorable than the former one, with a barrier of 9 kcal/mol (measured from **D**), that is, 2 kcal/mol higher than the one associated with the head-to-head coupling. This is in agreement with the regioselectivity experimentally observed for aromatic substrates (see Table 2 and its discussion above).

**Figure 3:**
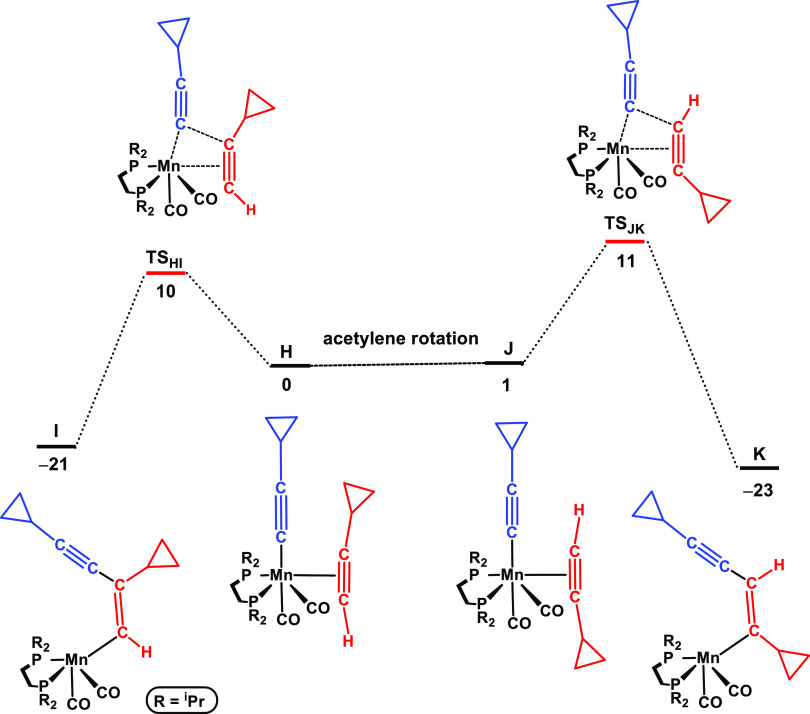
Free energy profile calculated for the regioselectivity of aliphatic alkynes. Left side: head-to-tail coupling and right side: head-to-head coupling. Free energies (kcal/mol) are referred to [Mn(dippe)(CO)_2_(C≡CPh)(η^2^-HC≡CPh)] (**D** in the calculations).

Interestingly, the equivalent study for an aliphatic substrate reveals the opposite result. The corresponding free energy profile (Figure 3) shows that the barrier for head-to-tail coupling and the formation of the corresponding terminal vinyl species (**I**) is 1 kcal/mol lower than the one calculated for head-to-head coupling that results in internal *E*-vinyl complex **K**. This result corroborates the observed regioselectivity for aliphatic substrates (see Table 3 and its discussion above).

**Figure 4:**
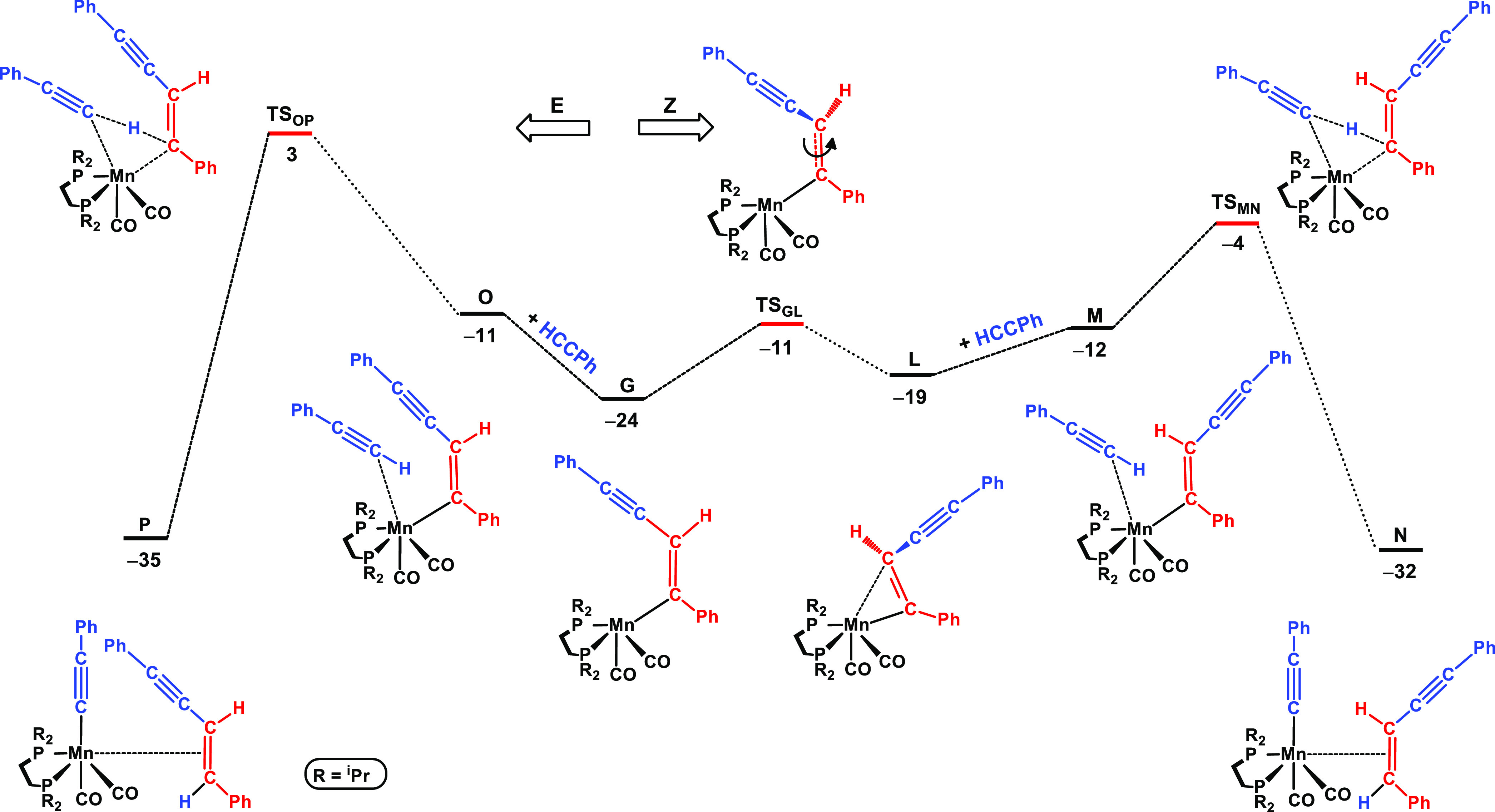
Free energy profile calculated for the formation of the enyne product from vinyl intermediate **G**. Left side: formation of the *E*-product and right side: formation of the *Z*-product. Free energies (kcal/mol) are referred to [Mn(dippe)(CO)_2_(C≡CPh)(η^2^-HC≡CPh)] (**D** in the calculations).

The free energy profile obtained for the conclusion of the reaction is depicted in Figure 4, corresponding to steps **III**–**V** in the cycle given in Scheme 3. Here, we start from *E*-vinyl intermediate **G** that results from the head-to-head coupling of the initial acetylene complex and is the last species on the right side of Figure 2. The reaction stereoselectivity is addressed in the profile of Figure 4 with the formation of *E*-enyne represented on the left-hand side and the path leading to the *Z*-isomer depicted on the right-hand side.

From **G** to **L**, there is *E*–*Z* isomerization of the vinyl ligand, a process that overcomes a barrier of Δ*G*^‡^ = 13 kcal/mol (**TS**_**GL**_) and has a balance of Δ*G* = 5 kcal/mol, the *Z*-vinyl complex (**L**) being less stable than its *E* counterpart (**G**). From **L**, the addition of acetylene leads to the final step in the mechanism, with the protonation of the vinyl ligand and release of the final product, while the acetylide ligand is regenerated. The barrier associated with this process from a pair of molecules, acetylene and the vinyl intermediate, in **M**, to the acetylide complex with the coordinated *Z*-enyne product, in **N**, is Δ*G*^‡^ = 8 kcal/mol (**TS**_**MN**_), and the step is clearly favorable, from the thermodynamic point of view, with Δ*G* = −20 kcal/mol. Importantly, the equivalent step for the formation of *E*-enyne (left side of Figure 4) has a barrier 7 kcal/mol higher (**TS**_**OP**_ vs **TS**_**MN**_), corroborating the observed stereoselectivity and preferential formation of the *Z*-enyne product.

Closure of the catalytic cycle from **N** back to acetylene complex **D** with product release and addition of a fresh PhC≡CH substrate has a free energy of Δ*G* = −7 kcal/mol. The highest barrier along the path is Δ*G*^‡^ = 20 kcal/mol, measured from intermediate **G** to **TS**_**MN**_, the transition state for acetylene addition, vinyl protonation, and regeneration of the acetylide ligand. Interestingly, this step corresponds to H-transfer and being the rate-determining step corroborates the kinetic isotopic effect experimentally detected and discussed above (Scheme 2).

## Conclusions

In summary, efficient additive-free manganese-catalyzed dimerization of terminal alkynes to afford enynes is described. To the best of our knowledge, this is the first example of a well-defined Mn(I)-based catalyst for such a process. The precatalyst is the alkyl bisphosphine Mn(I) complex *fac*-[Mn(dippe)(CO)_3_ (CH_2_CH_2_CH_3_)] (**1**), which is air-stable for several weeks in the solid state. The initiation step involves the migratory insertion of a CO ligand into the Mn–alkyl bond followed by C–H bond activation of the terminal alkyne to form the 16e^–^ Mn(I) acetylide complex [Mn(dippe)(CO)_2_(C≡CR)]. This species is an efficient catalyst for the regio- and stereoselective head-to-head dimerization of terminal aromatic and aliphatic alkynes, giving *Z*-1,3-enynes and head-to-tail *gem*-1,3-enynes, respectively, in high yields with up to 99% selectivity. Moreover, complex **1** is also capable to promote cross-dimerizations of aromatic alkynes with aliphatic alkynes, yielding selectively geminal 1,3-enynes. DFT calculations disclosed an acetylene-vinyl mechanism. In the case of aromatic alkynes, *Z-*1,3-enyne is a kinetic product, resulting from a lower energy barrier, compared with the one associated with the formation of its *E* counterpart. Based on the kinetic isotope effects of 1.49 and 2.44 detected for the dimerization of phenylacetylene versus phenylacetylene-*d*_1_ and 1-octyne versus 1-octyne-*d*_1_, respectively, the activation of the C–H bond appears to be the rate-determining step. These findings are in line with theoretical calculations since the C–H activation upon product release shows the highest energy barrier in the catalytic reaction.

## Supporting Information Available

^1^H and ^13^C{^1^H} NMR spectra of all compounds and complete computational details (PDF)
Cartesian coordinates for DFT-optimized structures (XYZ)

## Supplementary Material

cs1c01137_si_001.pdf

cs1c01137_si_002.xyz
