# Supplementary material for: Selective Manganese-Catalyzed Dimerization and Cross-Coupling of Terminal Alkynes
Source: ACS Catal. 2021 May 18;11(11):6474–83. doi: 10.1021/acscatal.1c01137 (PMC8185884; doi:10.1021/acscatal.1c01137)
Supplement: Supplementary file 1 — cs1c01137_si_001.pdf [file cs1c01137_si_001.pdf]

Supporting Information

Selective Manganese-Catalyzed Dimerization and Cross-Coupling of Terminal Alkynes

Stefan Weber,[†] Luis F. Veiros,[§] and Karl Kirchner*[, †]

[†]Institute of Applied Synthetic Chemistry, Vienna University of Technology, Getreidemarkt 9, A-1060 Vienna, AUSTRIA

[§] Centro de Química Estrutural and Departamento de Engenharia Química, Instituto Superior Técnico, Universidade de Lisboa, Av Rovisco Pais, 1049-001 Lisboa, PORTUGAL

E-mail: karl.kirchner@tuwien.ac.at

1. General information 2
2. Synthetic procedures 3
   Optimization reactions for the dimerization of phenylacetylene 3
   General procedure for the dimerization of aromatic alkynes 5
   General procedure for the dimerization of aliphatic alkynes 5
   General procedure for cross coupling of alkynes 6
   Cross coupling of alkynes in big scale 6
   Detection of 1-butanal 8
   Dimerization of Ph-C≡^{13}C-H 9
3. Computational Details 11
4. Characterization of organic products 11
5. NMR Spectra 19
6. References 44

1. General information

All reactions were performed under an inert atmosphere of argon by using Schlenk techniques or in a MBraun inert-gas glovebox. The solvents were purified according to standard procedures. The deuterated solvents were purchased from Aldrich and dried over 3 Å molecular sieves.

Complexes *fac*-[Mn(dippe)$(CO)_3$(Pr)][1] (dippe = 1,2-bis(di-*iso*-propylphosphino)ethane) (**1**), *fac*-[Mn(dprpe)$(CO)_3$(Pr)][1] (dprpe = 1,2-bis(dipropylphosphino)ethane) (**2**), *fac*-[Mn(dpppe)$(CO)_3$(Me)][2] (dppe = 1,2-bis(diphenylphosphino)ethane) (**3**), *fac*-[Mn(dippe)$(CO)_3$(Br)][3] (**4**) and *fac*-[Mn(dippe)$(CO)_3$(H)][4] (**5**) were synthesized according to literature. Phenylacetylene-d_1 (>98 % D) and 1-octyne-d_1 (>98 % D) was synthesized from phenylacetylene, 1-octyne, *n*-BuLi and D_2O. Phenylacetylene-^{13}C (50% ^{13}C enriched on the terminal carbon) was synthesized according to literature, starting from 50% ^{13}C enriched CH_3I.[5]

^{1}H- and $^{13}C\{^{1}H\}$- NMR spectra were recorded on a Bruker AVANCE-400 spectrometer. ^{1}H and $^{13}C\{^{1}H\}$-NMR spectra were referenced internally to residual protio-solvent and solvent resonances, respectively, and are reported relative to tetramethylsilane (δ = 0 ppm).

GC–MS analysis was conducted on a ISQ LT Single quadrupole MS (Thermo Fisher) directly interfaced to a TRACE 1300 Gas Chromatographic systems (Thermo Fisher), using a Rxi-5Sil MS (30 m, 0.25mm ID) cross-bonded dimethyl polysiloxane capillary column.

High-resolution accurate mass spectra were recorded on an Agilent 6545 QTOF equipped with an Agilent MMI ion source (Agilent Technologies, Santa Clara, CA, USA) which can be operated in mixed ESI and APCI mode. Measured accurate mass data for confirming calculated elemental compositions were within ±3 ppm accuracy.

2. Synthetic Procedures

Optimization reactions for the dimerization of phenylacetylene

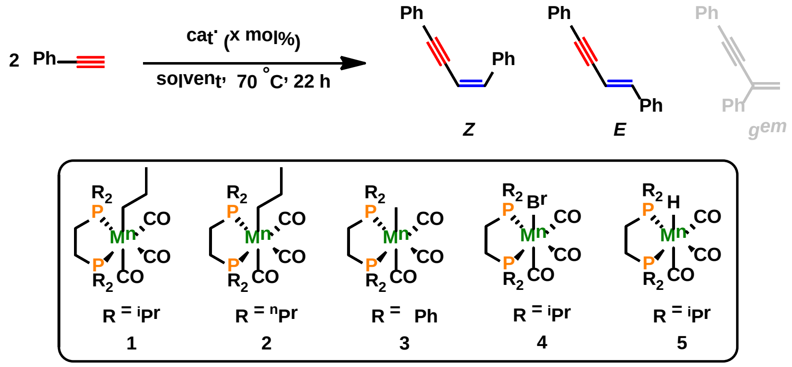

Inside an Ar-flushed glovebox an 8 mL-screw cap vial was charged with complex **1-5** (0.1 - 2 mol%) and phenylacetylene (1.1 mmol, 1 eq.). A stirring bar was added and 0.5 mL solvent were added. The vial was sealed, transferred out of the glovebox, and heated (if required) to the indicated temperature for the indicated time. The color of the mixture changes from colorless to dark brown upon reaction progress. The reaction mixture was allowed to reach room temperature and exposed to air. A sample was taken for GC-MS analysis.

<u>Full Table of optimization reaction</u>

Table S1. Full table of optimization reaction for the dimerization of phenylacetylene catalyzed by complex **1**.

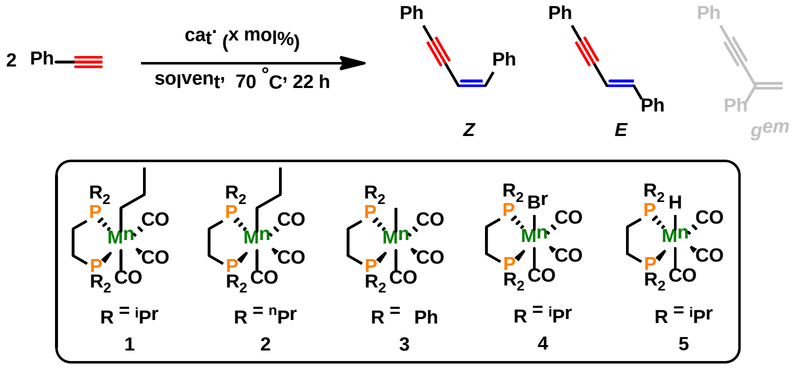

entry	catalyst (mol%)	solvent	conversion [%]	*Z:E ratio*
1	**1** (2)	THF	>99	96:4
2	2 (2)	THF	67	79:21
3	3 (2)	THF	12	73:27

4	**4** (2)	THF	-	n.d.
5	**5** (2)	THF	-	n.d.
6	**1** (2)	DME	97	95:5
7	**1** (2)	iPrOH	97	97:3
8	**1** (2)	toluene	17	95: 5
9	**1** (2)	$CHCl_3$	12	97:3
**10**[a]	**1 (1)**	**THF**	**>99**	**97:3**
11[a]	**1** (1)	neat	95	96:4
12[a]	**1** (0.5)	THF	82	96:4
13[a,b]	**1** (0.5)	THF	traces	n.d.
14[a,b]	(0.5)	neat	38	98:2
15[a]	1 (0.1)	THF	26	91:9
16[a,c]	1 (1)	THF	traces	n.d.
17[a,d]	1 (1)	THF	94	95:5

Reaction conditions: phenylacetylene (1.1 mmol), 0.5 mL anhydrous solvent, 70 °C, Ar, 22 h, conversion and isomer ratio determined by GC-MS. [a] 18 h. [b] 25 °C. [c] on air. [d] in 4 M aqueous THF solution.

General procedure for the dimerization of aromatic alkynes

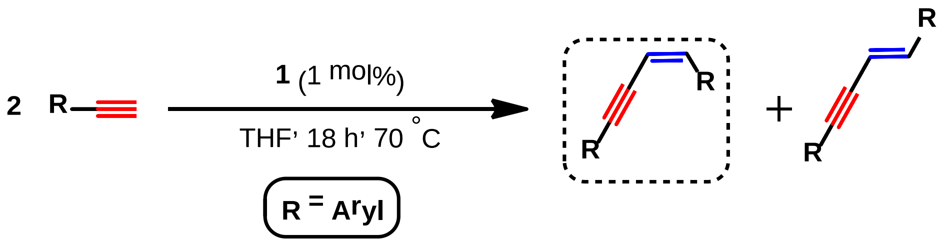

Inside an Ar-flushed glovebox an 8 mL-screw cap vial was charged with **1** (4.5 mg, 1 mol%) and substrate (1.1 mmol, 1 eq.). A stirring bar was added and 0.5 mL THF were added. The vial was sealed, transferred out of the glovebox, and heated to 70 °C for 18 h. The color of the mixture changes from colorless to dark brown upon heating. The reaction mixture was allowed to reach room temperature and exposed to air. A sample was taken for GC-MS analysis. The solvent was removed under reduced pressure. The crude products were isolated by flash chromatograph (2-3 g silica 60 in a Pasteur pipette) using light petroleum/Et_2O as eluent.

General procedure for the dimerization of aliphatic alkynes

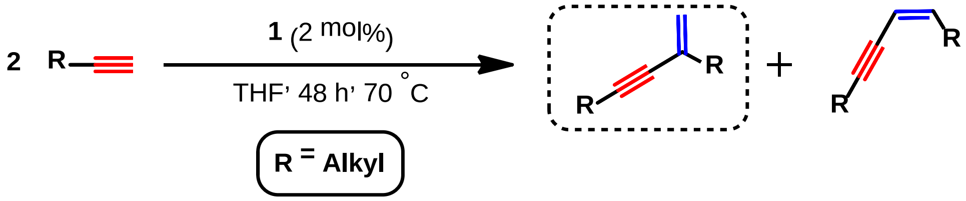

Inside an Ar-flushed glovebox an 8 mL-screw cap vial was charged with **1** (9 mg, 2 mol%) and substrate (1.1 mmol, 1 eq.). A stirring bar was added and 0.5 mL THF were added. The vial was sealed, transferred out of the glovebox, and heated to 70 °C for 48 h. The color of the mixture changes from colorless to orange upon heating. The reaction mixture was allowed to reach room temperature and exposed to air. A sample was taken for GC-MS analysis. The solvent was removed under reduced pressure. The crude products were isolated by flash chromatograph (2-3 g silica 60 in a Pasteur pipette) using light petroleum/Et_2O as eluent.

General procedure for cross coupling of alkynes

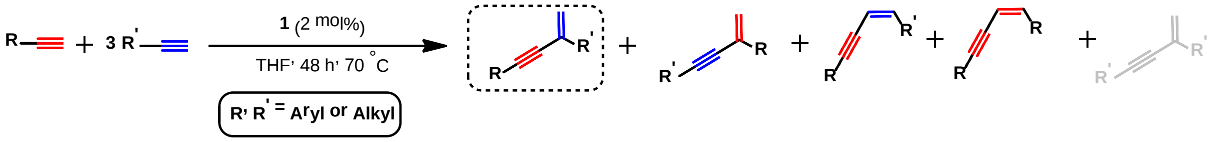

Inside an Ar-flushed glovebox an 8 mL-screw cap vial was charged with **1** (9 mg, 2 mol%), alkyne (1.1 mmol, 1 eq.) and the coupling partner alkyne (3.3 mmol, 3 eq.). A stirring bar was added and 0.5 mL THF were added. The vial was sealed, transferred out of the glovebox, and heated to 70 °C for 48 h. The color of the mixture changes from colorless to red upon heating. The reaction mixture was allowed to reach room temperature and exposed to air. A sample was taken for GC-MS analysis. The solvent was removed under reduced pressure. The crude products were isolated by flash chromatograph.

Cross coupling of alkynes in big scale

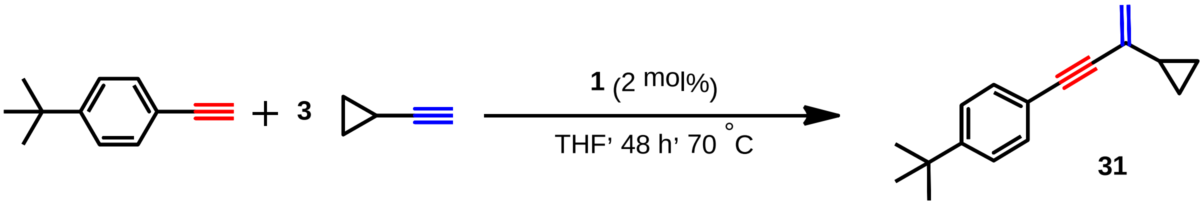

31

Inside an Ar-flushed glovebox an 20 mL-microwave vial was charged with **1** (90 mg, 2 mol%), ^{t}Bu-phenylacetylene (11 mmol, 1 eq.) and cyclopropylacetylene (33 mmol, 3 eq.). A stirring bar was added and 5 mL THF were added. The vial was sealed, transferred out of the glovebox, and heated to 70 °C for 48 h. The color of the mixture changes from colorless to red upon heating. The reaction mixture was allowed to reach room temperature and exposed to air. A sample was taken for GC-MS analysis. The solvent was removed under reduced pressure. The crude products were isolated by flash chromatograph using PE as eluent, yielding **31** (1.36 g, 55%)

Additional table of cross coupling of alkynes

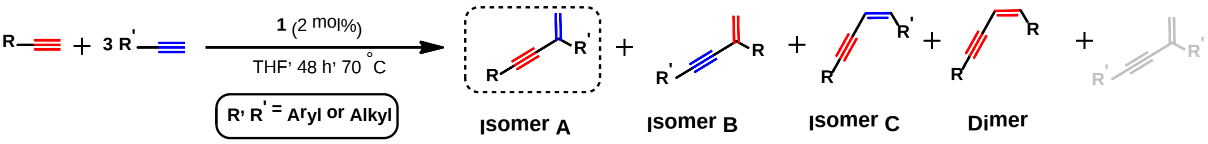

Table S2. Isomer ratio for the cross coupling of alkynes

Alkyne 1 (1 eq.)	Alkyne 2 (3 eq.)	Conversion Alkyne 1 [%]	A [%]	B [%]	C [%]	Dimer [%]
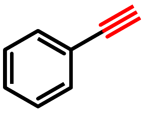	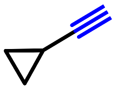	>99	52	21	9	18
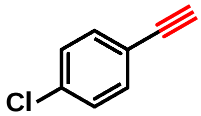	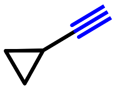	>99	38	18	3	32
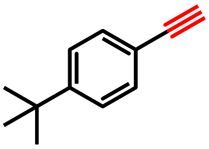	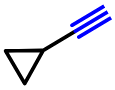	>99	58	18	9	15
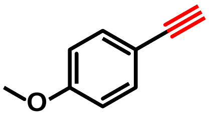	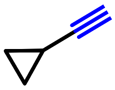	>99	60	22	10	7
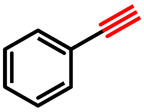	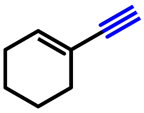	>99	48	32	n.d.	21
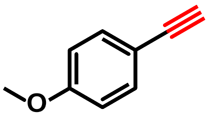	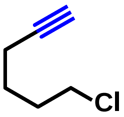	98	72	15	n.d.	10
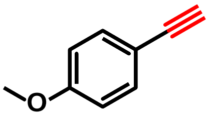	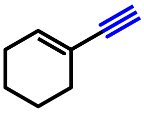	96	84	n.d.	n.d.	12
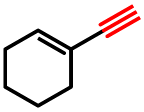	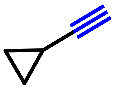	80	47	15	6	5

3. Experimental Mechanistic Studies

Detection of 1-butanal

Inside an Ar-flushed glovebox an NMR tube was charged with **1** (4.5 mg, 1 mol%) and phenylacetylene (1.1 mmol, 1 eq.). 0.5 mL THF-*d8* were added. The NMR tube was sealed, transferred out of the glovebox and heated to 70 °C for 2 h. ^{1}H-NMR was recorded.

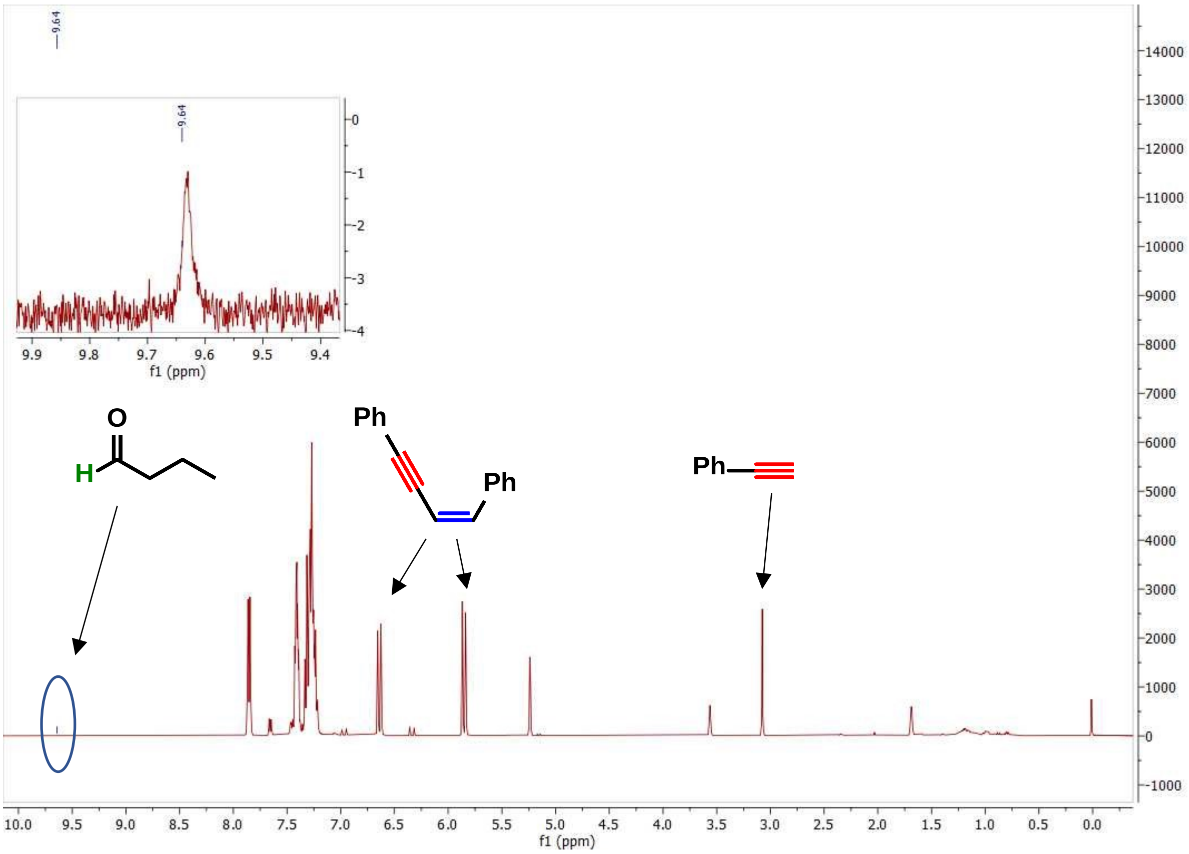

Dimerization of Ph-C≡^{13}C-H

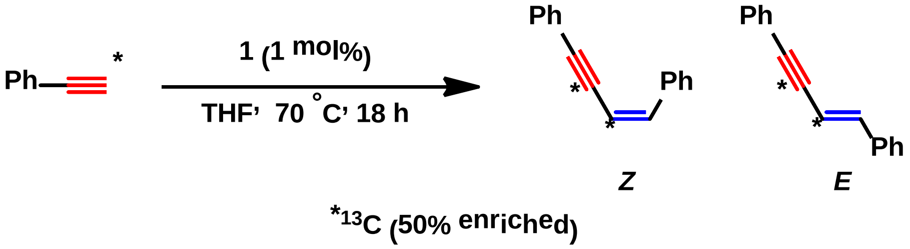

Inside an Ar-flushed glovebox an 8 mL-screw cap vial was charged with complex **1** (1 mol%) and Ph-C≡^{13}C-H (50% ^{13}C-labeled; 1.1 mmol, 1 eq.). A stirring bar was added and 0.5 mL solvent were added. The vial was sealed, transferred out of the glovebox, and stirred for 18 h at 70 °C. The color of the mixture changes from colorless to dark brown upon reaction progress. The reaction mixture was allowed to reach room temperature and exposed to air. A sample was taken for GC-MS analysis. The solvent was gently removed and the residue was purified by column chromatography, using 10 g silica and PE as eluent, yielding compound **6*** (^{13}C enriched) as yellow oil (91%).

^{1}H NMR (δ, 400 MHz, CD_2Cl_2, 20 °C): 7.88 – 7.81 (m, 2H), 7.43 – 7.40 (m, 1H), 7.40 – 7.32 (m, 1H), 7.32 – 7.17 (m, 6H), 6.64 (dt, $J_{3H\text{-}H}$ = 11.9, 7.6 Hz (J_2-^{13}C- coupling) 1H,), 6.09 – 5.60 (m, (due to J_1-^{13}C- coupling), 1H).

^{13}C{^{1}H} NMR (δ, 101 MHz, CD_2Cl_2, 20 °C): 107.2 (s, no direct J_1-^{13}C- coupling partner due to only 50% ^{13}C enrichment), 107.2 (d, 88.7 Hz J_1-^{13}C- coupling partner), 88.1 (s, no direct J_1-^{13}C- coupling partner due to only 50% ^{13}C enrichment), 88.1 (d, 88.7 Hz J_1-^{13}C- coupling partner); only ^{13}C enriched signals of *Z*-isomer reported.

The J_1-^{13}C-^{13}C coupling constant is consistent with a similar 1,3-enyne in the literature.[6]

No J_2-^{13}C-^{13}C coupling could be detected, which would be present in case of an 1,2-aryl-shift among the C≡C bond.

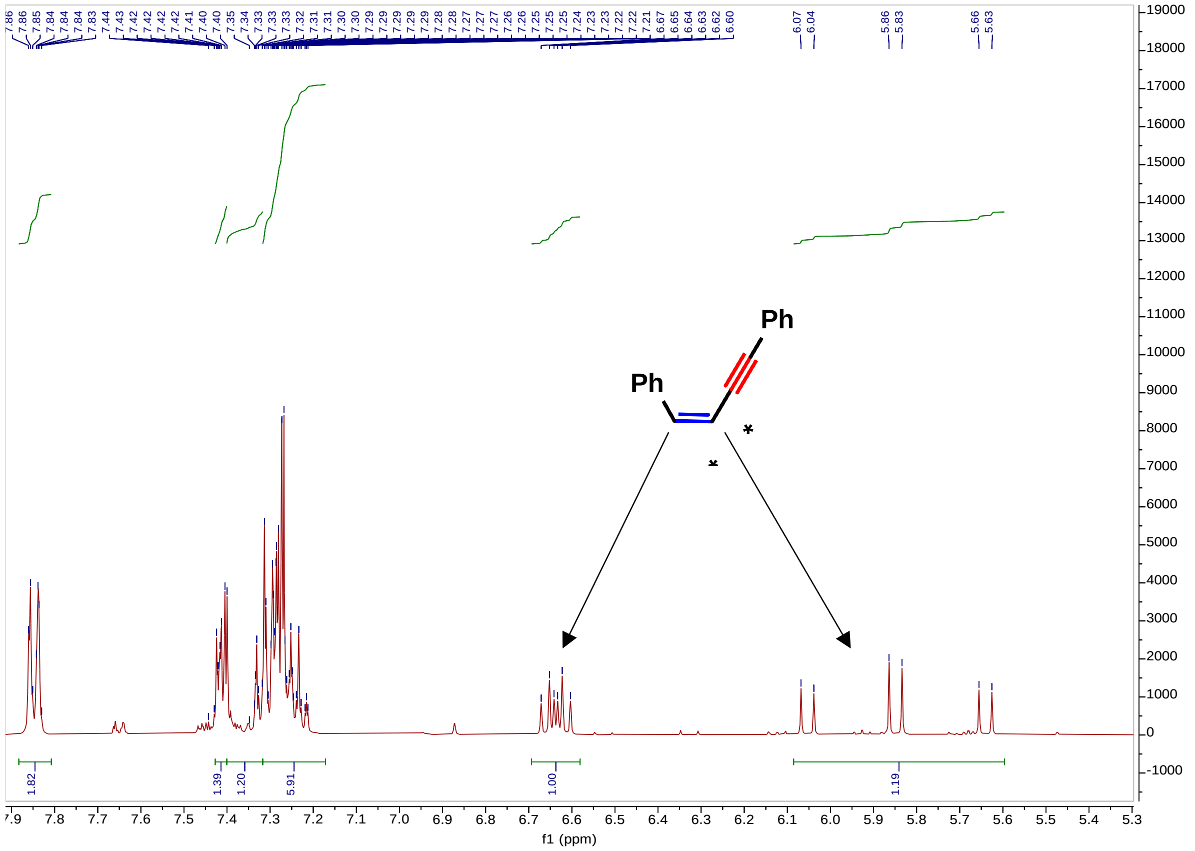
Ph
Ph
1.82
1.39
1.20
5.91
1.00
1.19
f1 (ppm)

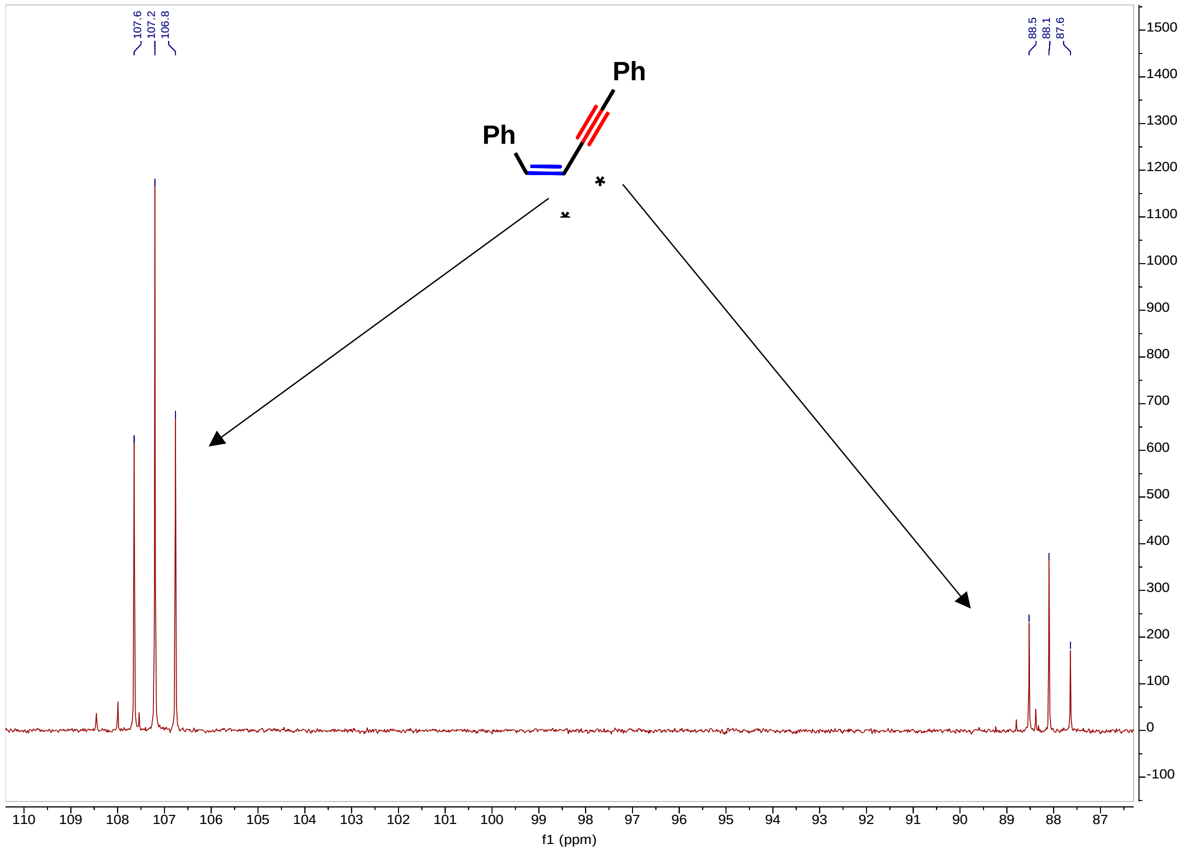
107.6
107.2
106.8
88.5
88.1
87.6
Ph
Ph
f1 (ppm)

4. Computational Details

DFT Calculations: The computational results presented have been achieved in part using the Vienna Scientific Cluster (VSC). All calculations were performed using the GAUSSIAN 09 software package.[7] All calculations were performed with the PBE0 functional without symmetry constraints and a basis set consisting of the Stuttgart/Dresden ECP (SDD) basis set[8] to describe the electrons of Mn, and a standard 6-31G(d,p) basis set[9] for all other atoms. The PBE0 functional uses a hybrid generalized gradient approximation (GGA), including 25 % mixture of Hartree-Fock[10] exchange with DFT[11] exchange-correlation, given by Perdew, Burke and Ernzerhof functional (PBE).[12] Transition state optimizations were performed with the Synchronous Transit-Guided Quasi-Newton Method (STQN) developed by Schlegel *et al*,[13] following extensive searches of the Potential Energy Surface. Frequency calculations were performed to confirm the nature of the stationary points, yielding one imaginary frequency for the transition states and none for the minima. Each transition state was further confirmed by following its vibrational mode downhill on both sides and obtaining the minima presented on the energy profiles. The electronic energies (E_{b1}) were converted to free energy at 298.15 K and 1 atm (G_{b1}) by using zero point energy and thermal energy corrections based on structural and vibration frequency data calculated at the same level.

5. Characterization of organic products

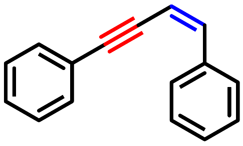

(Z)-but-1-en-3-yne-1,4-diyldibenzene[14] (**6**), yellow oil, 106 mg (92%). ^{1}H NMR (δ, 400 MHz, CD_2Cl_2, 20 °C): 7.87 – 7.81 (m, 2H), 7.46 – 7.36 (m, 2H), 7.35 – 7.19 (m, 6H), 6.63 (d, J = 11.9 Hz, 1H), 5.84 (d, J = 11.9 Hz, 1H) ppm. ^{13}C{^{1}H} NMR (δ, 101 MHz, CD_2Cl_2, 20 °C): 138.7, 136.6, 131.4, 128.7, 128.6, 128.5, 128.3, 123.4, 107.2, 95.8, 88.1 ppm.

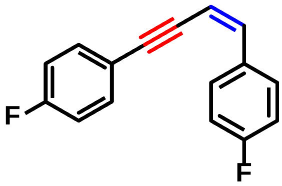

(Z)-4,4'-(but-1-en-3-yne-1,4-diyl)bis(fluorobenzene)[14] (**7**), yellow oil, 114 mg (92%). ^{1}H NMR (δ, 400 MHz, CD_2Cl_2, 20 °C): 8.04 – 7.81 (m, 2H), 7.59 – 7.42 (m, 2H), 7.15 – 7.03 (m, 4H), 6.69 (d, J = 12.0 Hz, 1H), 5.90 (d, J = 11.9 Hz, 1H) ppm. ^{13}C{^{1}H} NMR (δ, 101 MHz, CD_2Cl_2, 20 °C): 162.7 (d, J_{C-F} = 250 Hz), 162.5 (d, J_{C-F} = 249 Hz), 137.4, 133.4, 133.3, 132.9, 132.9, 130.6, 130.5, 115.9, 115.6, 115.3, 115.3, 106.8, 106.7, 94.6, 87.6 ppm.

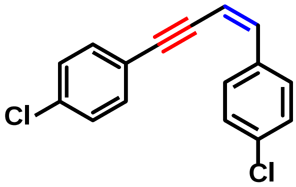

(Z)-4,4'-(but-1-en-3-yne-1,4-diyl)bis(chlorobenzene)[15] (**8**), purified by column chromatography (15g SiO_2, PE/Et_2O 1:1) slightly yellow solid, 99 mg (92%). ^1H NMR (δ, 400 MHz, CD_2Cl_2, 20 °C): 7.90 – 7.82 (m, 2H), 7.49 – 7.31 (m, 6H), 6.70 (d, J = 11.9 Hz, 1H), 5.95 (d, J = 12.0 Hz, 1H) ppm. $^{13}C\{^1H\}$ NMR (δ, 101 MHz, CD_2Cl_2, 20 °C): 139.2, 136.6, 136.1, 135.6, 134.3, 131.6, 130.4, 130.1, 123.3, 109.2, 96.7, 90.3 ppm.

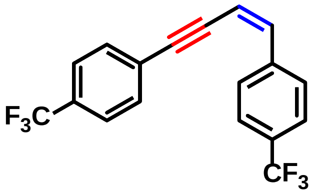

(Z)-4,4'-(but-1-en-3-yne-1,4-diyl)bis(trifluoromethylbenzene)[15] (**9**), brownish oil, 166 mg (96%). ^1H NMR (δ, 400 MHz, CD_2Cl_2, 20 °C): 8.03 (dt, J = 8.8, 0.8 Hz, 2H), 7.70 – 7.60 (m, 6H), 6.84 (d, J = 11.9 Hz, 1H), 6.09 (d, J = 11.9 Hz, 1H) ppm. $^{13}C\{^1H\}$ NMR (δ, 101 MHz, CD_2Cl_2, 20 °C): 141.3, 139.7, 133.4, 131.9, 131.7, 131.5, 131.4, 130.5, 128.4, 127.1 - 126.8 (m, -CF_3), 124.4, 124.2, 111.0, 96.6, 91.2 ppm.

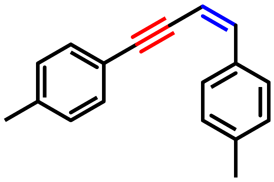
(Z)-4,4'-(but-1-en-3-yne-1,4-diyl)bis(methylbenzene)[14] (**10**), yellow oil, which slowly crystallized upon standing, 81 mg (69%). ^1H NMR (δ, 400 MHz, CD_2Cl_2, 20 °C): 7.88 – 7.81 (m, 2H), 7.44 – 7.36 (m, 2H), 7.28 – 7.15 (m, 5H), 6.67 (d, J = 11.9 Hz, 1H), 5.87 (d, J = 11.9 Hz, 1H), 2.38 (s, 6H) ppm. $^{13}C\{^1H\}$ NMR (δ, 101 MHz, CD_2Cl_2, 20 °C): 140.6, 140.6, 140.1, 135.8, 133.1, 131.0, 130.8, 130.5, 122.2, 108.1, 97.6, 89.6, 23.1, 22.9 ppm.

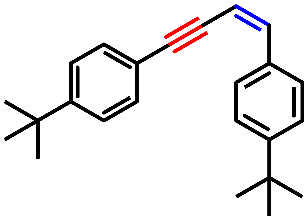
(Z)-4,4'-(but-1-en-3-yne-1,4-diyl)bis(tert-butylbenzene)[14] (**11**), purified by column chromatography (15g SiO_2, PE/Et_2O 3:1) yellow solid, 125 mg (78%). ^1H NMR (δ, 400 MHz, CD_2Cl_2, 20 °C): 7.95 – 7.88 (m, 2H), 7.52 – 7.39 (m, 7H), 6.70 (d, J = 11.9 Hz, 1H), 5.90 (d, J = 11.9 Hz, 1H), 1.37 (s, 9 H), 1.36 (s, 9H) ppm. $^{13}C\{^1H\}$ NMR (δ, 101 MHz,

CD_2Cl_2, 20 °C): 153.6, 139.9, 135.6, 132.8, 130.2, 127.4, 127.2, 127.0, 122.2, 108.2, 97.6, 89.6, 36.4, 36.4, 32.7, 32.6 ppm.

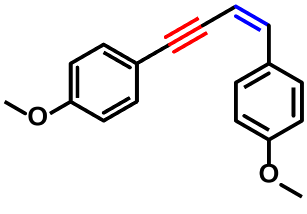
(Z)-4,4'-(but-1-en-3-yne-1,4-diyl)bis(methoxybenzene)[14] (**12**), purified by column chromatography (15g SiO_2, PE/Et_2O 1:1) yellow solid, 84 mg (63%). ^{1}H NMR (δ, 400 MHz, CD_2Cl_2, 20 °C): 7.95 – 7.88 (m, 2H), 7.50 – 7.42 (m, 2H), 6.97 – 6.87 (m, 4H), 6.63 (d, J = 11.9 Hz, 1H), 5.81 (d, J = 11.8 Hz, 1H), 3.84 (s, 3H), 3.83 (s, 3H) ppm. ^{13}C{^{1}H} NMR (δ, 101 MHz, CD_2Cl_2, 20 °C): 161.5, 161.5, 139.0, 134.5, 131.8, 131.4, 130.0, 127.5, 117.3, 115.8, 115.3, 106.6, 97.1, 59.03, 57.0, 56.9 ppm.

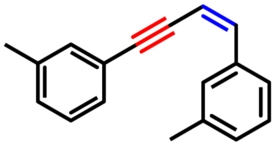
(Z)-3,3'-(but-1-en-3-yne-1,4-diyl)bis(methylbenzene)[15] (**15**), yellow oil, 93 mg (79%). ^{1}H NMR (δ, 400 MHz, CD_2Cl_2, 20 °C): 7.83 (td, J = 1.8, 0.9 Hz, 1H), 7.72 (dt, J = 7.7, 1.5 Hz, 1H), 7.36 – 7.23 (m, 4H), 7.20 - 7.12 (m, 2H), 6.69 (d, J = 12.0 Hz, 1H), 5.91 (d, J = 11.9 Hz, 1H), 2.40 (s, 3H), 2.36 (s, 3H) ppm. ^{13}C{^{1}H} NMR (δ, 101 MHz, CD_2Cl_2, 20 °C): 140.5, 140.1, 139.7, 138.3, 133.7, 131.1, 131.1, 130.2, 130.1, 130.0, 127.7, 125.0, 108.8, 97.7, 89.8, 23.0, 22.7 ppm.

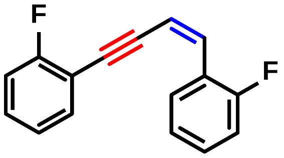

(Z)-2,2'-(but-1-en-3-yne-1,4-diyl)bis(fluorobenzene)[15] (**16**), slightly yellow oil, 118 mg (97%). ^{1}H NMR (δ, 400 MHz, CD_2Cl_2, 20 °C): 8.62 (td, J = 7.8, 1.8 Hz, 1H), 7.48 (td, J = 7.4, 1.9 Hz, 1H), 7.42 – 7.28 (m, 2H), 7.27 – 7.06 (m, 4H), 7.02 (d, J = 12.1 Hz, 1H), 6.07 (d, J = 12.1 Hz, 1H) ppm. ^{13}C{^{1}H} NMR (δ, 101 MHz, CD_2Cl_2, 20 °C): 165.7, 163.2, 163.2, 160.7, 135.0, 132.2, 132.2, 132.1, 132.0, 130.4, 130.2, 125.9, 125.9, 125.8, 125.6, 125.5, 117.3, 117.1, 117.0, 116.7, 113.4, 113.3, 110.2, 94.2, 91.5 ppm.

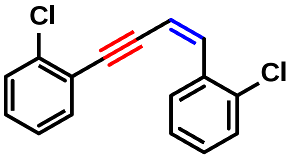

(Z)-2,2'-(but-1-en-3-yne-1,4-diyl)bis(chlorobenzene)[15] (**17**), brownish solid, 131 mg (95%). ^{1}H NMR (δ, 400 MHz, CD_2Cl_2, 20 °C): 8.62 – 8.54 (m, 1H), 7.53 – 7.47

(m, 1H), 7.45 – 7.40 (m, 2H), 7.37 – 7.22 (m, 4H), 7.16 (d, J = 12.0 Hz, 1H), 6.13 (d, J = 12.1 Hz, 1H) ppm. $^{13}C\{^1H\}$ NMR (δ, 101 MHz, CD_2Cl_2, 20 °C): 137.5, 137.2, 135.8, 135.4, 135.4, 131.6, 131.3, 131.2, 128.5, 128.4, 124.8, 111.0, 94.7, 93.9 ppm.

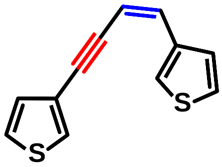

(Z)-3,3'-(but-1-en-3-yne-1,4-diyl)dithiophene[14] (**18**), brownish oil, 89 mg (81%). ^1H NMR (δ, 400 MHz, $CDCl_3$, 20 °C): 7.81 (d, J = 3.1, 1H), 7.69 (d, J = 5.1, 1H), 7.50 (d, J = 3.0), 7.31 (s, 2H), 7.19 (d, J = 5.0, 1H), 6.73 (d, J = 11.7 Hz, 1H), 5.79 (d, J = 11.6 Hz, 1H) ppm. $^{13}C\{^1H\}$ NMR (δ, 101 MHz, $CDCl_3$, 20 °C): 138.8, 132.7, 129.6, 128.5, 127.8, 125.6, 125.5, 125.2, 122.5, 106.0, 91.1, 88.1 ppm.

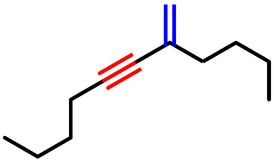

7-methyleneundec-5-yne[16] (**20**), slightly yellow liquid, 70 mg (85%). ^1H NMR (δ, 400 MHz, $CDCl_3$, 20 °C): 5.20 (dt, J = 2.2, 0.7 Hz, 1H), 5.12 (dt, J = 2.5, 1.3 Hz, 1H), 2.31 (t, J = 7.0 Hz, 2H), 2.16 – 2.07 (m, 2H), 1.56 – 1.39 (m, 7H), 1.32 (dt, J = 8.4, 7.0 Hz, 2H), 0.91 (td, J = 7.3, 5.2 Hz, 6H).$^{13}C\{^1H\}$ NMR (δ, 101 MHz, $CDCl_3$, 20 °C): 142.6, 119.3, 90.0, 81.3, 37.3, 30.9, 30.3, 22.0, 22.0, 19.0, 13.9, 13.6.

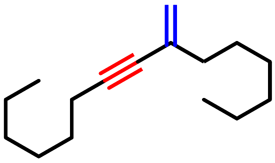

9-methylenepentadec-7-yne[16] (**21**), colorless liquid, 99 mg (89 %). ^1H NMR (δ, 400 MHz, $CDCl_3$, 20 °C): 5.13 (d, *J* = 2.5 Hz, 1H), 5.05 (q, *J* = 1.5 Hz, 1H), 2.23 (t, *J* = 7.0 Hz, 2H), 2.04 (t, *J* = 7.6 Hz, 2H), 1.51 – 1.39 (m, 4H), 1.39 – 1.29 (m, 3H), 1.29 – 1.15 (m, 12H), 0.91 - 0.77 (m, 6H). $^{13}C\{^1H\}$ NMR (δ, 101 MHz, $CDCl_3$, 20 °C): 142.6, 119.3, 91.0, 81.0, 37.6, 31.7, 31.4, 28.8, 28.6, 28.6, 28.1, 22.6, 22.6, 19.3, 14.1, 14.0.

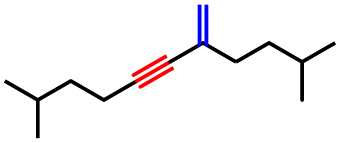

2,10-dimethyl-7-methyleneundec-5-yne (**22**), colorless oil, 91 mg (93 %). ^1H NMR (δ, 400 MHz, $CDCl_3$, 20 °C): 5.19 (dt, J = 2.1, 0.7 Hz, 1H), 5.14 – 5.11 (m, 1H), 2.31 (t, J = 7.4 Hz, 2H), 2.15 – 2.09 (m, 2H), 1.70 (dq, J = 13.4, 6.7 Hz, 1H), 1.57 (dp, J =

13.3, 6.6 Hz, 1H), 1.47 – 1.36 (m, 4H), 0.90 (dd, J = 6.6, 5.0 Hz, 12H). $^{13}C\{^{1}H\}$ NMR (δ, 101 MHz, $CDCl_3$, 20 °C): 142.8, 119.1, 90.1, 81.0, 37.7, 37.6, 35.5, 27.4, 27.2, 22.5, 22.2, 17.3. HRMS (APCI) *m/z* calculated for $C_{14}H_{24}$ $[M-H]^+$: 193.1878, found 193.1847.

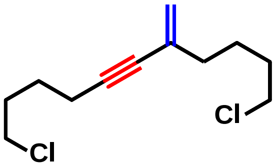

1,11-dichloro-7-methyleneundec-5-yne[16] (**23**), colorless oil, 111 mg (94%). %). ^{1}H NMR (δ, 400 MHz, $CDCl_3$, 20 °C): 5.25 – 5.21 (m, 1H), 5.16 (q, J = 1.5 Hz, 1H), 3.60 – 3.56 (m, 2H), 3.56 – 3.52 (m, 2H), 2.36 (t, J = 6.9 Hz, 2H), 2.16 (t, J = 7.3 Hz, 2H), 1.96 – 1.86 (m, 2H), 1.83 – 1.76 (m, 2H), 1.74 – 1.64 (m, 4H). $^{13}C\{^{1}H\}$ NMR (δ, 101 MHz, $CDCl_3$, 20 °C): 141.7, 120.4, 89.4, 81.3, 44.9, 44.6, 36.6, 31.7, 31.6, 25.9, 25.3, 18.6.

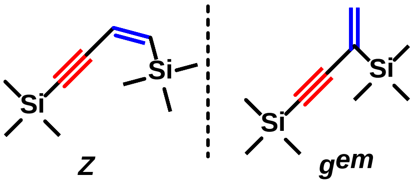

(Z)-but-1-en-3-yne-1,4-diylbis(trimethylsilane) (*Z*-siomer) and but-3-en-1-yne-1,3-diylbis(trimethylsilane) (*gem*-isomer)[14] (**25**), colorless oil, 48 mg (48%) combined yield of *Z*-isomer and *gem*-isomer. ^{1}H NMR (δ, 400 MHz, $CDCl_3$, 20 °C): 6.26 (d, J = 15.1 Hz, 1H)(*Z*-isomer), 6.16 (d, J = 15.1 Hz, 1H)(*Z*-isomer), 6.12 (d, J = 3.4 Hz, 1H) (*gem*-isomer), 5.70 (d, J = 3.4 Hz, 1H) (*gem*-isomer), 0.19 (s, 9H) (Z-isomer), 0.19 (s, 9H) (Z-isomer), 0.19 (s, 6H) (*gem*-isomer), 0.16 (s, 6H) (*gem*-isomer) ppm. $^{13}C\{^{1}H\}$ NMR (δ, 101 MHz, $CDCl_3$, 20 °C): 146.5, 135.2, 135.0, 124. 9, 106.9, 105.4, 98.9, 98.9, 0.4, 0.0, -0.4, -1.95 ppm.

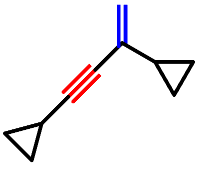

But-3-en-1-yne-1,3-diyldicyclopropane[17] (**26**), colorless liquid, 53 mg (74%). ^{1}H NMR (δ, 400 MHz, $CDCl_3$, 20 °C): 5.24 (d, J = 2.0 Hz, 1H), 5.18 (d, J = 2.0 Hz, 1H), 1.56 – 1.45 (m, 1H), 1.35 – 1.20 (m, 2H), 1.15-0.98 (m, 1H), 0.84 – 0.75 (m, 1H), 0.72 – 0.61 (m, 4H) ppm. $^{13}C\{^{1}H\}$ NMR (δ, 101 MHz, $CDCl_3$, 20 °C): 134.2, 117.5, 93.7, 72.8, 16.6, 8.7, 5.7, 0.0.

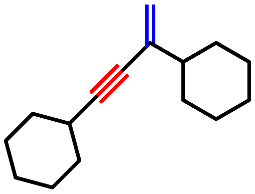

But-3-en-1-yne-1,3-diyldicyclohexane[18] (**27**), yellow oil, 103 mg (87%).^{1}H NMR (δ, 400 MHz, $CDCl_3$, 20 °C): 5.17 (d, J = 2.2 Hz, 1H), 5.13 – 5.09 (m, 1H), 1.92 – 1.59 (m, 16H), 1.56 – 1.39 (m, 6H), 1.39 – 1.02 (m, 16H) ppm. ^{13}C{^{1}H} NMR (δ, 101 MHz, $CDCl_3$, 20 °C): 138.0, 117.2, 94.8, 80.2, 45.3, 32.8, 32.34, 32.0, 31.8, 29.6, 26.3, 26.1, 26.0, 24.8, 20.2, 20.0 ppm.

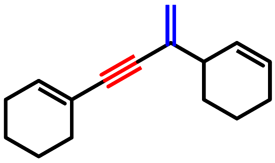

1-(3-(cyclohex-2-en-1-yl)but-3-en-1-yn-1-yl)cyclohex-1-ene[18] (**28**), yellowish oil, which slowly crystallizes upon standing, 98 mg (92%). ^{1}H NMR (δ, 400 MHz, $CDCl_3$, 20 °C): 6.10 (d, J = 11.9 Hz, 1H,), 6.07 – 6.04 (m, 1H), 5.95 (t, J = 3.4 Hz, 1H), 5.41 (d, J = 11.8 Hz, 1H), 2.61 (td, J = 6.2, 5.1, 3.2 Hz, 2H), 2.20 – 2.08 (m, 6H), 1.71 – 1.54 (m, 6H).^{13}C{^{1}H} NMR (δ, 101 MHz, $CDCl_3$, 20 °C): 141.5, 137.3, 134.1, 132.6, 121.3, 104.0, 96.2, 86.7, 28.9, 27.1, 26.1, 25.8, 22.7, 22.3, 22.0, 21.6.

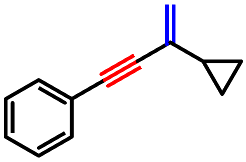

(3-cyclopropylbut-3-en-1-yn-1-yl)benzene[19] (**29**), purified by column chromatography (10 g SiO_2, PE), colorless oil, 65 mg (39%). ^{1}H NMR (δ, 400 MHz, $CDCl_3$, 20 °C): 7.46 – 7.39 (m, 2H), 7.34 – 7.28 (m, 3H), 5.42 (d, J = 1.7 Hz, 1H), 5.39 (d, J = 1.7 Hz, 1H), 1.64 (tt, J = 8.1, 5.0 Hz, 1H), 0.83 – 0.76 (m, 2H), 0.74 (ddt, J = 10.8, 5.2, 2.8 Hz, 2H).). ^{13}C{^{1}H} NMR (δ, 101 MHz, $CDCl_3$, 20 °C): 133.9, 131.3, 128.3, 128.2, 123.1, 118.9, 89.5, 86.6, 16.5, 5.9.

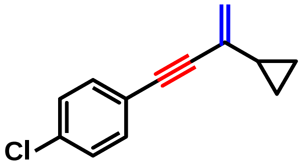

1-chloro-4-(3-cyclopropylbut-3-en-1-yn-1-yl)benzene (**30**), purified by column chromatography (10 g SiO_2, PE), colorless solid, 71 mg (35%). ^{1}H NMR (δ, 400 MHz, $CDCl_3$, 20 °C): 7.29 – 7.24 (m, 2H), 7.23 – 7.17 (m, 2H), 5.36 (d, J = 1.7 Hz, 1H), 5.31 (d, J = 1.7 Hz, 1H), 1.59 – 1.50 (m, 1H), 0.67 (tt, J = 8.1, 2.4 Hz, 4H). ^{13}C{^{1}H} NMR (δ, 101 MHz,

$CDCl_3$, 20 °C): 134.3, 133.6, 132.8, 128.6, 121.6, 119.4, 88.7, 88.0, 16.7, 5.95. HRMS (APCI) *m/z* calculated for $C_{13}H_{11}Cl$ [M-H]$^+$: 203.0549, found 203.0618.

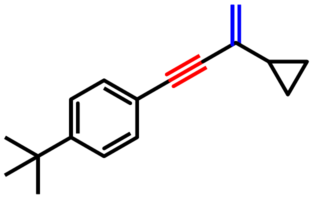

1-(tert-butyl)-4-(3-cyclopropylbut-3-en-1-yn-1-yl)benzene (**31**), purified by column chromatography (10 g SiO_2, PE), colorless oil, 119 mg (53%). ^{1}H NMR (δ, 400 MHz, $CDCl_3$, 20 °C): 7.37 – 7.34 (m, 2H), 7.32 (d, J = 8.8 Hz, 2H), 5.40 (d, J = 1.8 Hz, 1H), 5.36 (d, J = 1.8 Hz, 1H), 1.63 (tt, J = 8.4, 4.9 Hz, 1H), 1.31 (s, 9H), 0.82 – 0.76 (m, 2H), 0.76 – 0.68 (m, 2H). ^{13}C{^{1}H} NMR (δ, 101 MHz, $CDCl_3$, 20 °C): 151.5, 134.0, 131.4, 125.3, 120.1, 118.5, 89.6, 85.8, 30.8, 34.8, 16.5, 5.9. HRMS (APCI) *m/z* calculated for $C_{17}H_{20}$ [M-H]$^+$: 225.1565, found 225.1631.

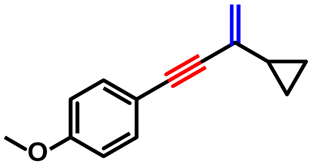

1-(3-cyclopropylbut-3-en-1-yn-1-yl)-4-methoxybenzene[20] (**32**), purified by column chromatography (10 g SiO_2, PE/EE 20:1), orange oil, 107 mg (54%). ^{1}H NMR (δ, 400 MHz, $CDCl_3$, 20 °C): 7.38 – 7.33 (m, 2H), 6.87 – 6.81 (m, 2H), 5.38 (d, J = 2.4 Hz, 1H), 5.35 (d, J = 1.8 Hz, 1H), 3.81 (s, 3H), 1.67 – 1.56 (m, 1H), 0.81 – 0.77 (m, 2H), 0.75 – 0.68 (m, 2H). ^{13}C{^{1}H} NMR (δ, 101 MHz, $CDCl_3$, 20 °C): 159.6, 134.1, 133.1, 118.2, 115.2, 113.9, 89.5, 85.2, 55.3, 16.5, 5.9.

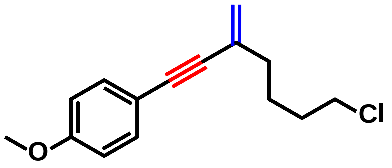

1-(7-chloro-3-methylenehept-1-yn-1-yl)-4-methoxybenzene (**34**), purified by column chromatography (10 g SiO_2, PE), orange oil, 161 mg (65%). ^{1}H NMR (δ, 400 MHz, $CDCl_3$, 20 °C): 7.38 (dd, J = 9.1, 2.5 Hz, 2H), 6.88 – 6.82 (m, 2H), 5.39 (d, J = 2.0 Hz, 1H), 5.27 (q, J = 1.4 Hz, 1H), 3.81 (s, 3H), 3.58 (t, J = 6.5 Hz, 2H), 2.31 – 2.24 (m, 2H), 1.88 – 1.81 (m, 2H), 1.79 – 1.72 (m, 2H). ^{13}C{^{1}H} NMR (δ, 101 MHz, $CDCl_3$, 20 °C): 159.6, 142.2, 133.1, 131.2, 120.9, 115.3, 110.1, 89.5, 88.2, 55.3, 44.9, 36.5, 31.8, 25.4. HRMS (APCI) *m/z* calculated for $C_{15}H_{17}ClO$ [M-H]$^+$: 249.0968, found 249.1043.

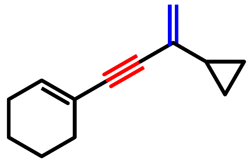

1-(3-cyclopropylbut-3-en-1-yn-1-yl)cyclohex-1-ene (**36**), purified by column chromatography (10 g SiO_2, PE), slightly yellow oil, 71 mg (41%). ^1H NMR (δ, 400 MHz, $CDCl_3$, 20 °C): 6.08 (tt, J = 3.6, 1.6 Hz, 1H), 5.30 (d, J = 1.8 Hz, 1H), 5.23 (d, J = 1.9 Hz, 1H), 2.15 – 2.04 (m, 4H), 1.66 – 1.50 (m, 5H), 0.73 – 0.61 (m, 4H). $^{13}C\{^1H\}$ NMR (δ, 101 MHz, $CDCl_3$, 20 °C): 135.1, 134.1, 117.8, 91.5, 83.8, 29.2, 25.7, 22.3, 21.5, 16.5, 5.8. HRMS (APCI) *m/z* calculated for $C_{13}H_{16}$ $[M-H]^+$: 173.1252, found 173.1325.

6. NMR Spectra

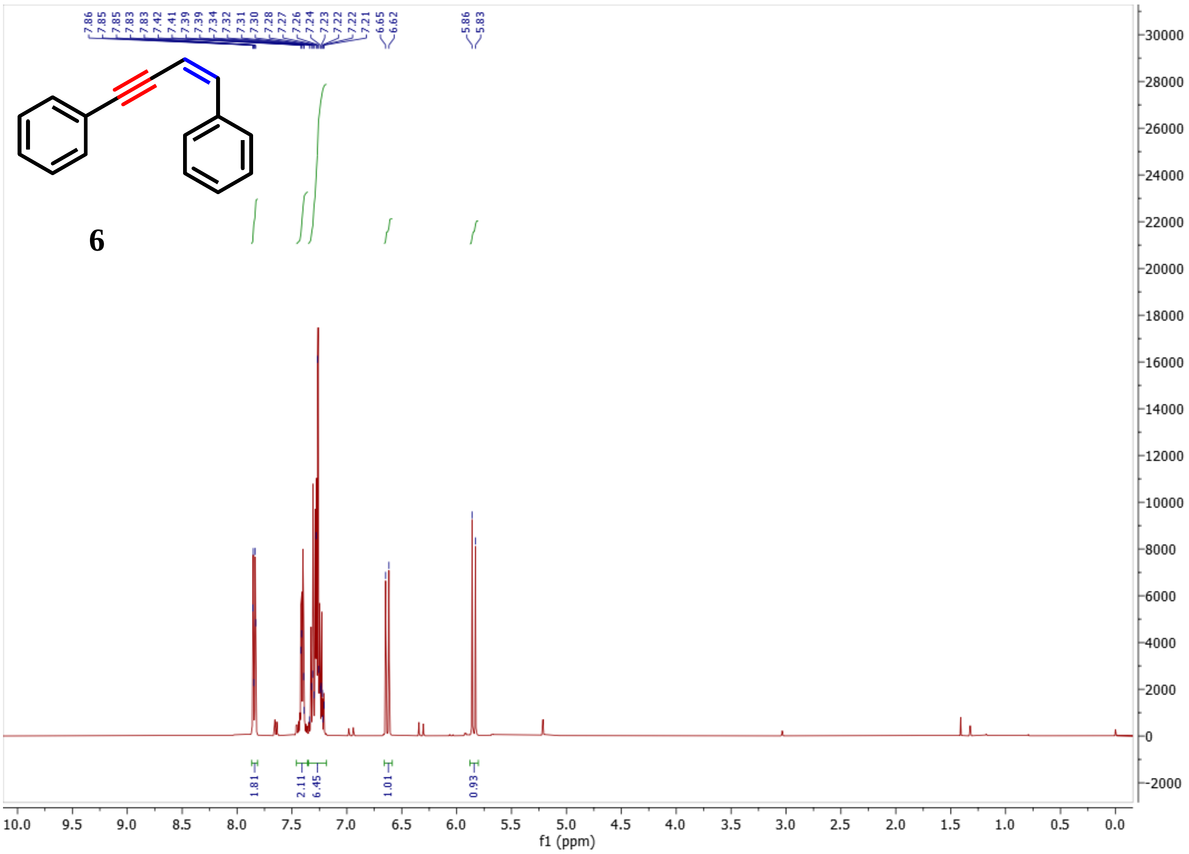

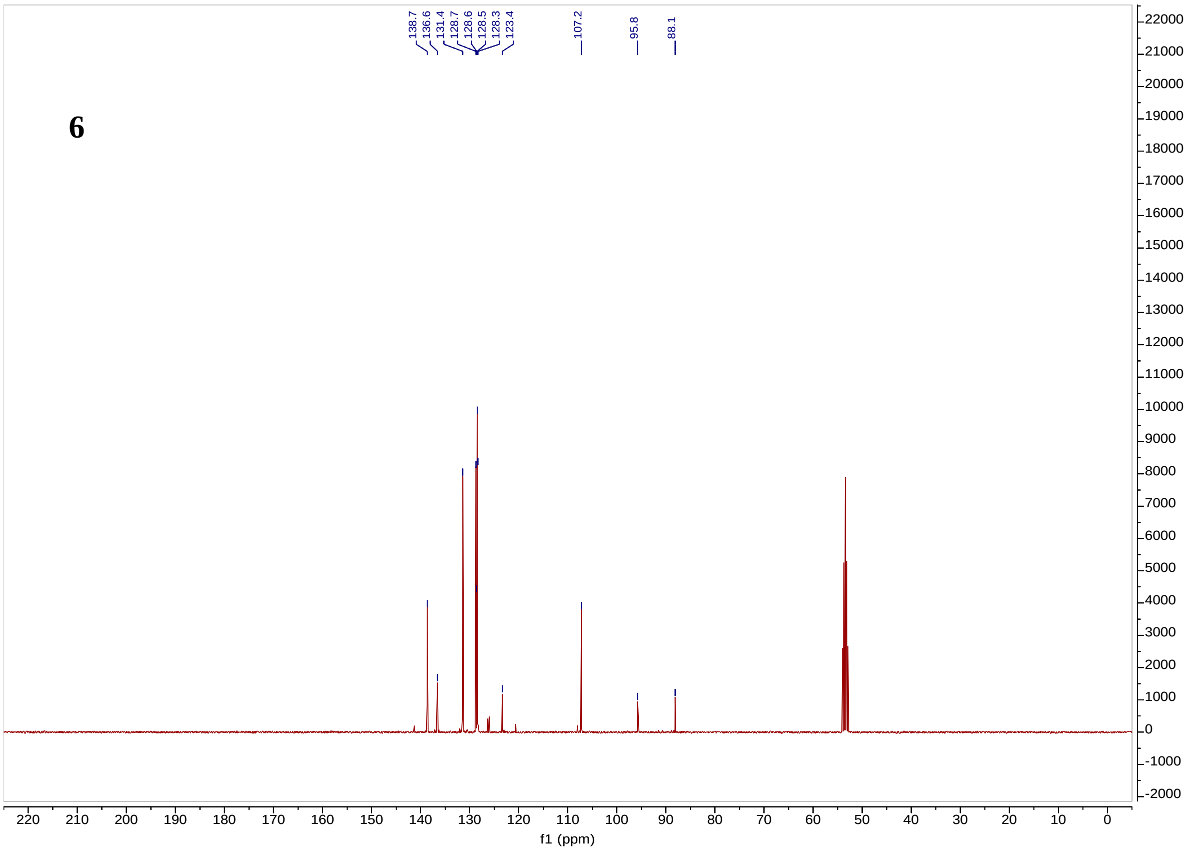

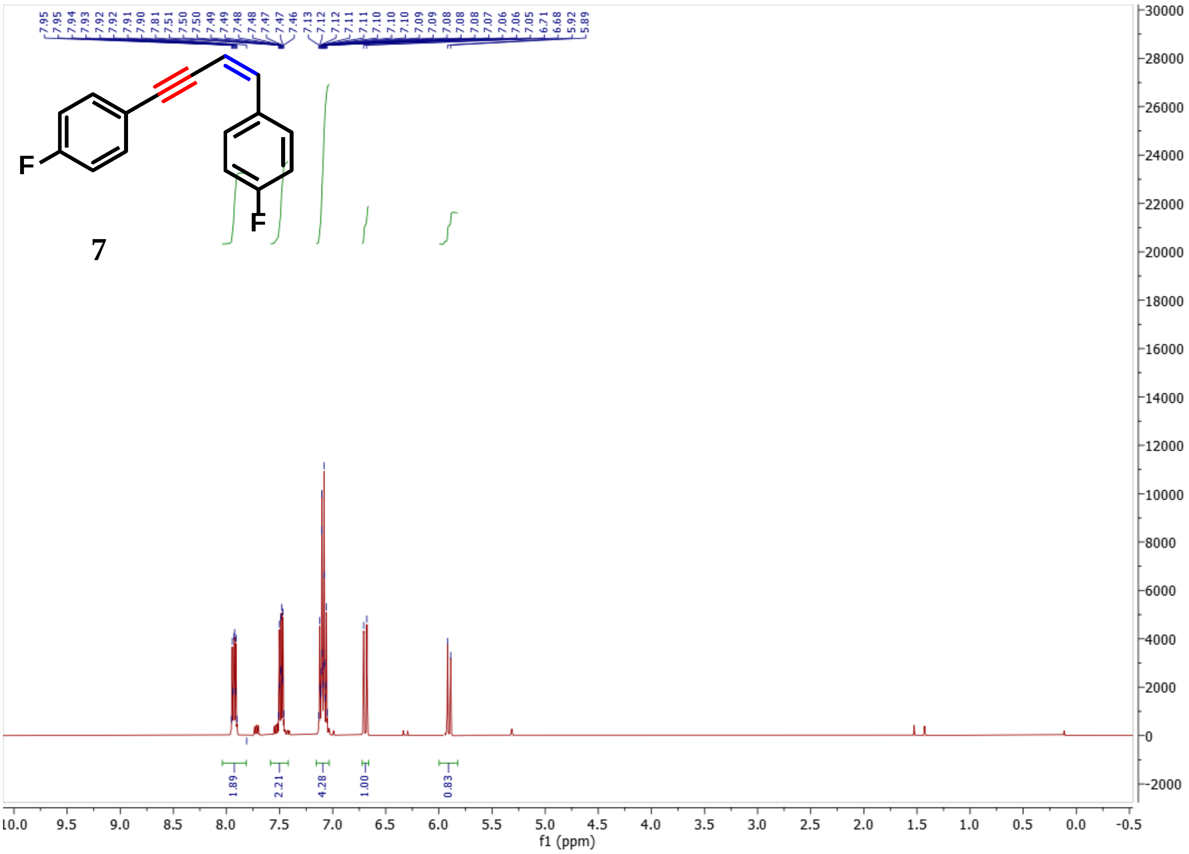
7
F
F
1.89
2.21
4.28
1.00
0.83
f1 (ppm)

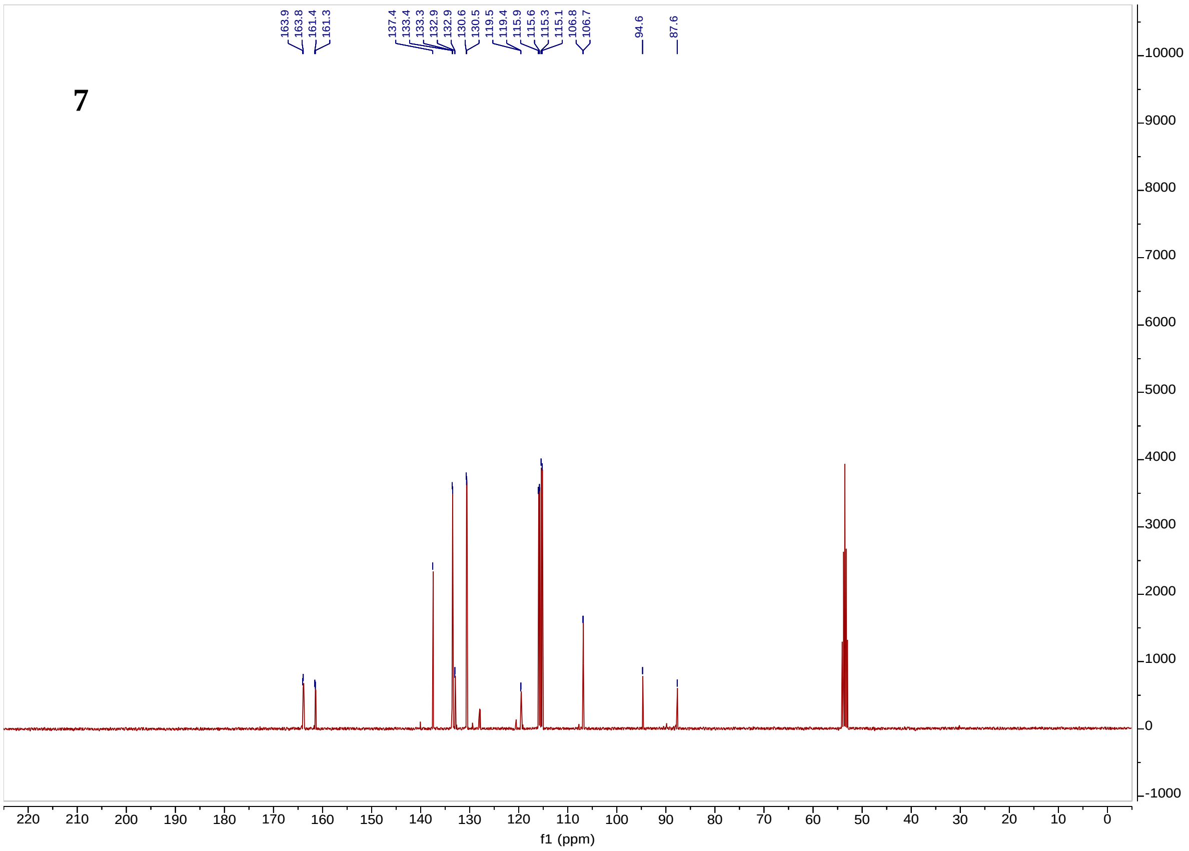
7
163.9
163.8
161.4
161.3
137.4
133.4
133.3
132.9
132.9
130.6
130.5
119.5
119.4
115.9
115.6
115.3
115.1
106.8
106.7
94.6
87.6
f1 (ppm)

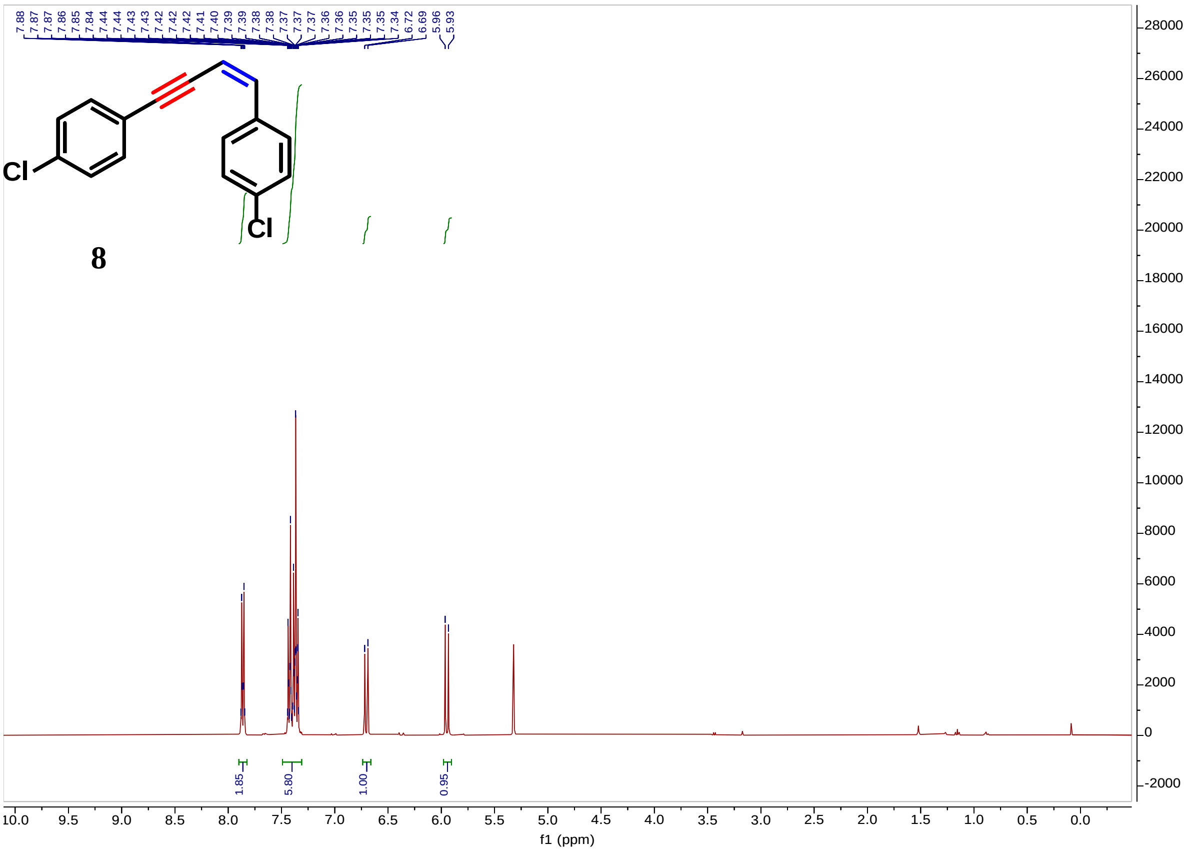

7.88 7.87 7.87 7.86 7.85 7.84 7.44 7.44 7.43 7.43 7.42 7.42 7.42 7.41 7.40 7.39 7.39 7.38 7.38 7.37 7.37 7.37 7.36 7.36 7.35 7.35 7.35 7.34 6.72 6.69 5.96 5.93
Cl
Cl
8
1.85
5.80
1.00
0.95
f1 (ppm)

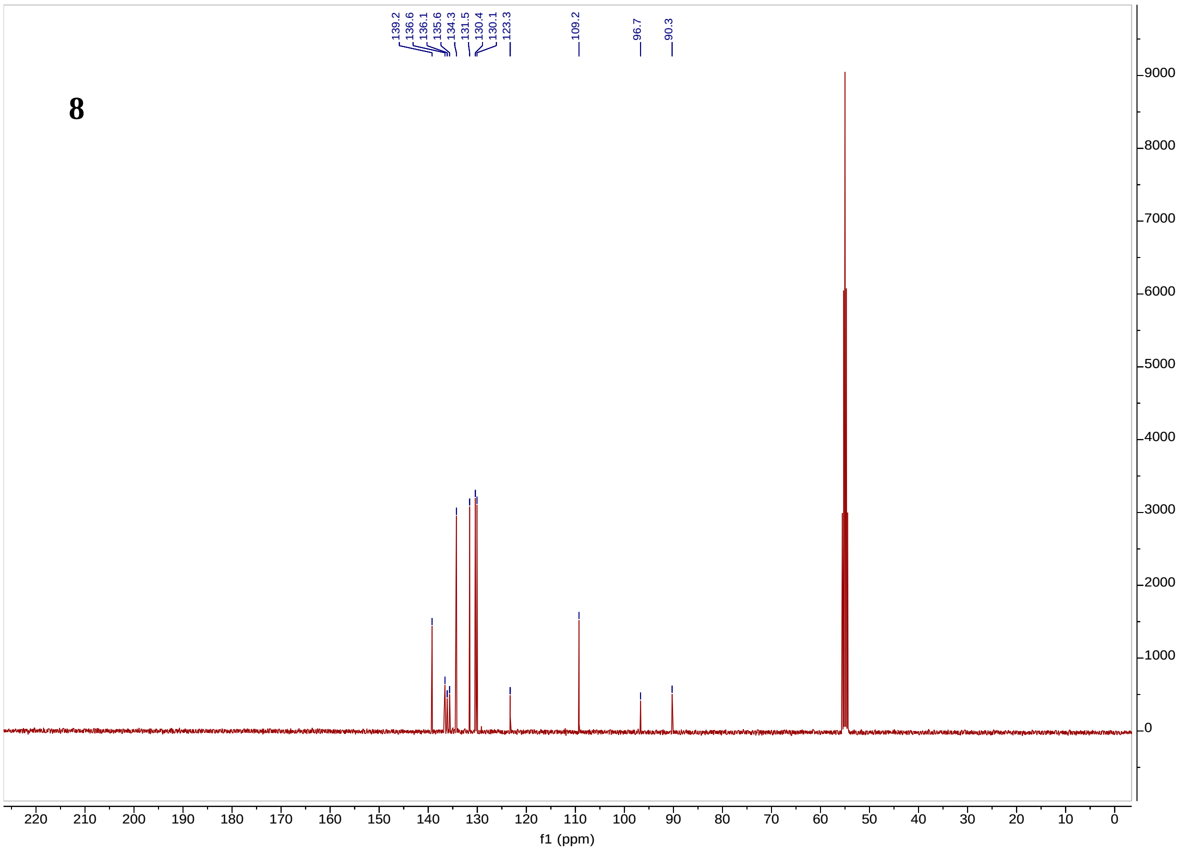

139.2 136.6 136.1 135.6 134.3 131.5 130.4 130.1 123.3
109.2
96.7
90.3
8
f1 (ppm)

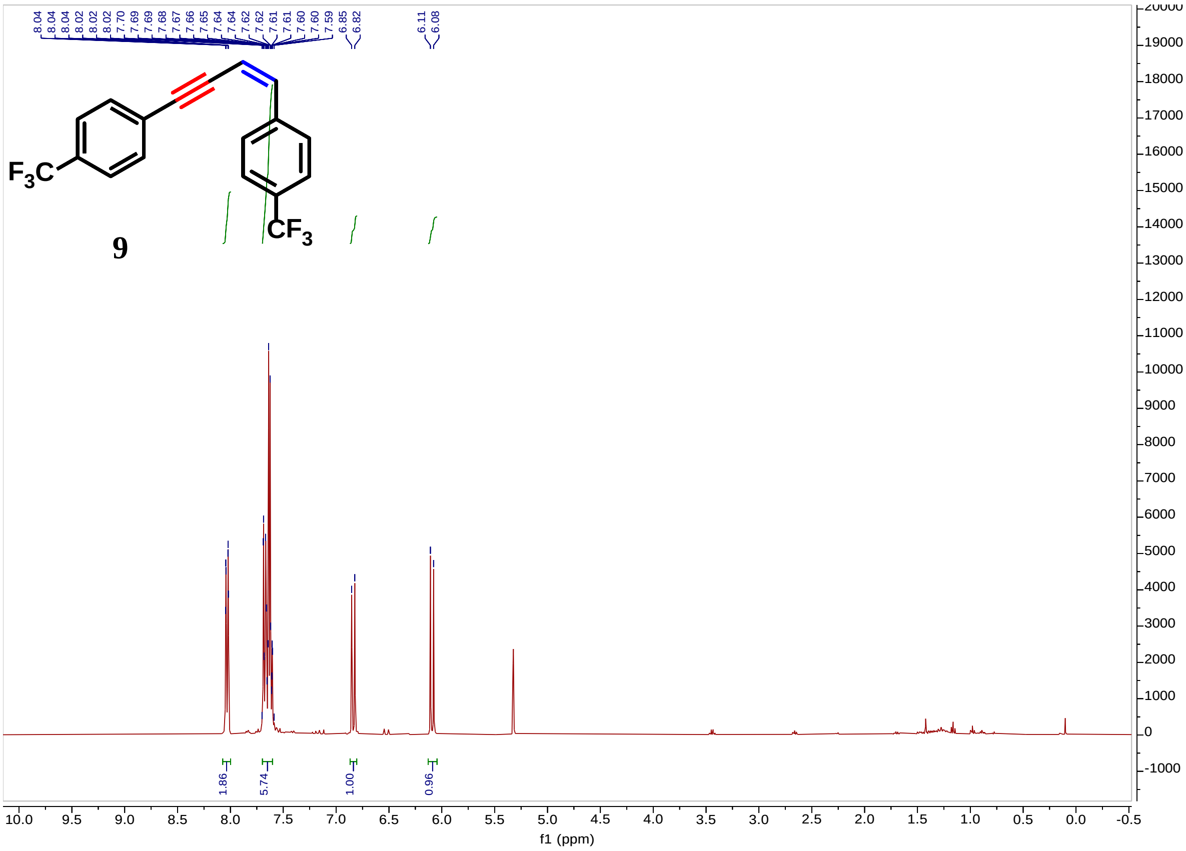
8.04 8.04 8.04 8.02 8.02 8.02 7.70 7.69 7.69 7.68 7.67 7.66 7.65 7.64 7.64 7.62 7.62 7.61 7.61 7.60 7.59 6.85 6.82 6.11 6.08
F3C
CF3
9
1.86
5.74
1.00
0.96
f1 (ppm)

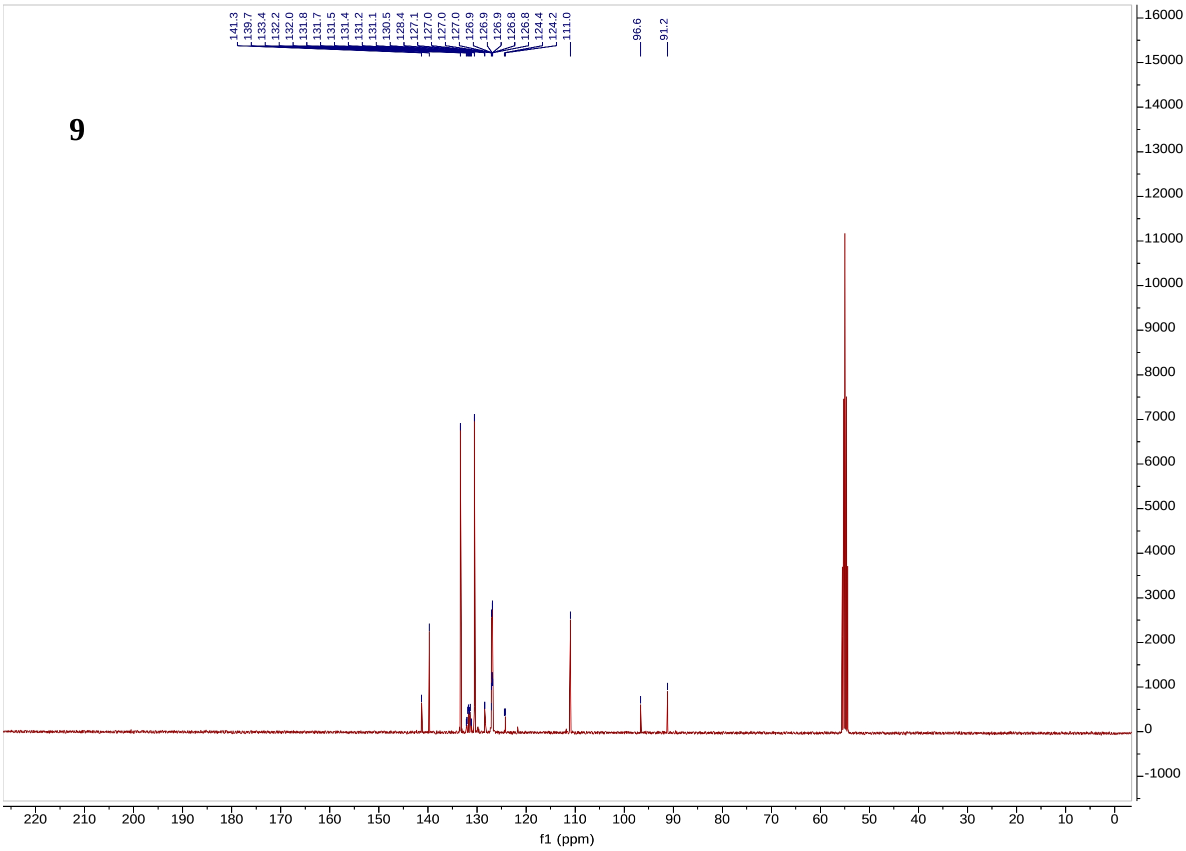
141.3 139.7 133.4 132.2 132.0 131.8 131.7 131.5 131.4 131.2 131.1 130.5 128.4 127.1 127.0 127.0 127.0 126.9 126.9 126.9 126.8 126.8 124.4 124.2 111.0
96.6
91.2
9
f1 (ppm)

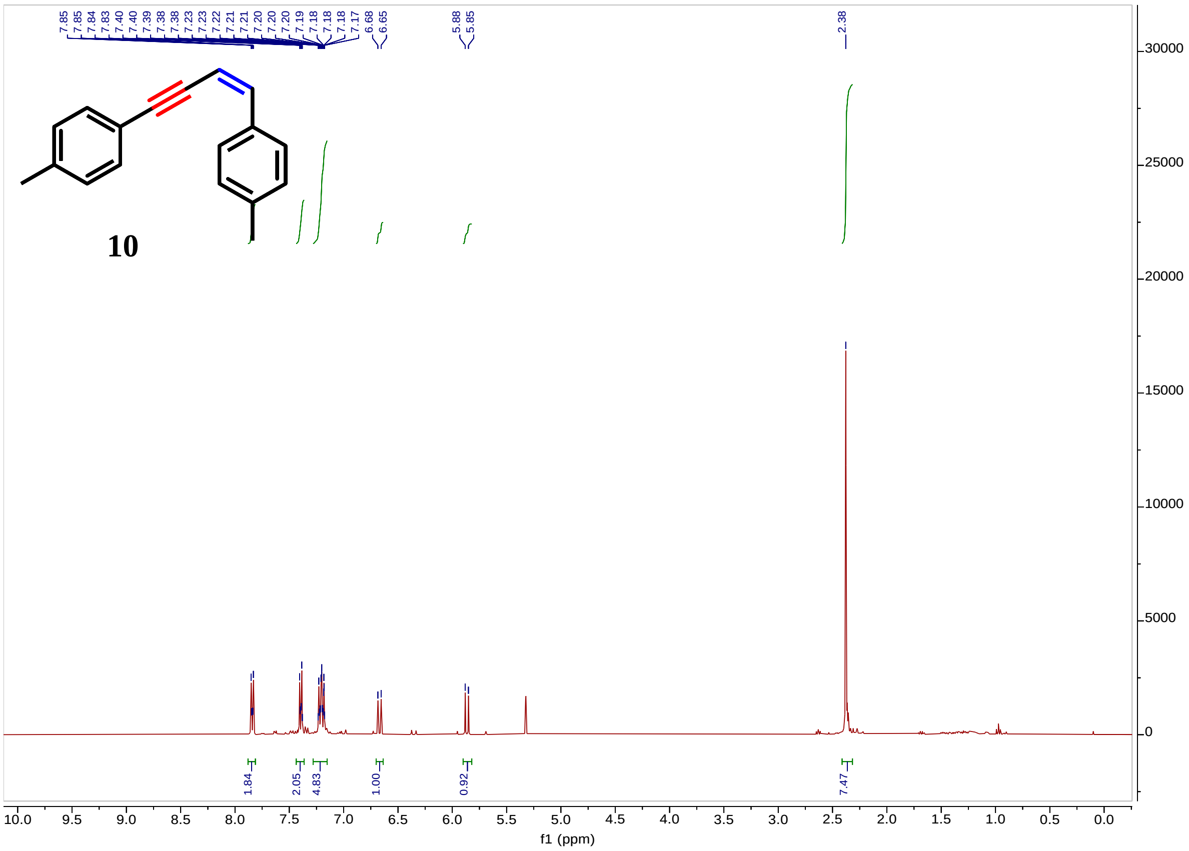
7.85
7.85
7.84
7.83
7.40
7.40
7.39
7.38
7.38
7.23
7.23
7.22
7.21
7.21
7.20
7.20
7.20
7.19
7.18
7.18
7.18
7.17
6.68
6.65
5.88
5.85
2.38
10
1.84
2.05
4.83
1.00
0.92
7.47
f1 (ppm)

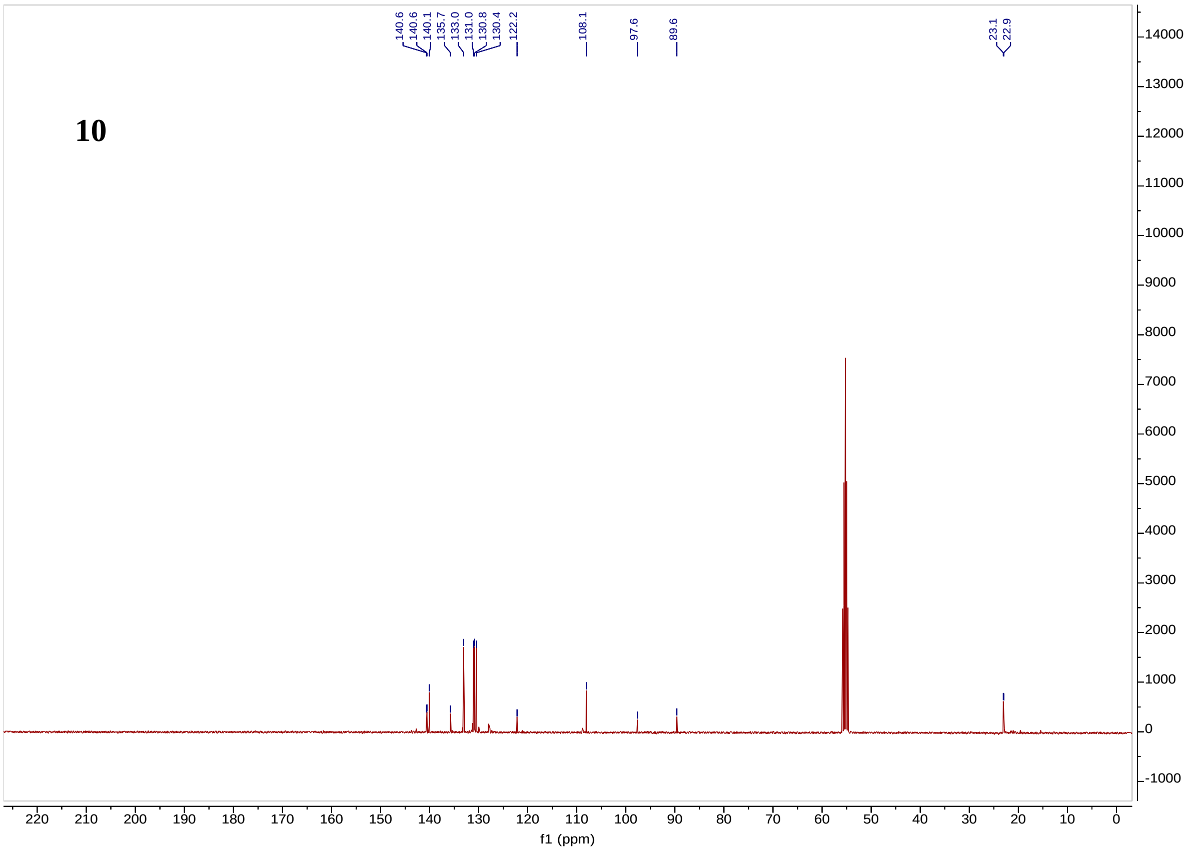
140.6
140.6
140.1
135.7
133.0
131.0
130.8
130.4
122.2
108.1
97.6
89.6
23.1
22.9
10
f1 (ppm)

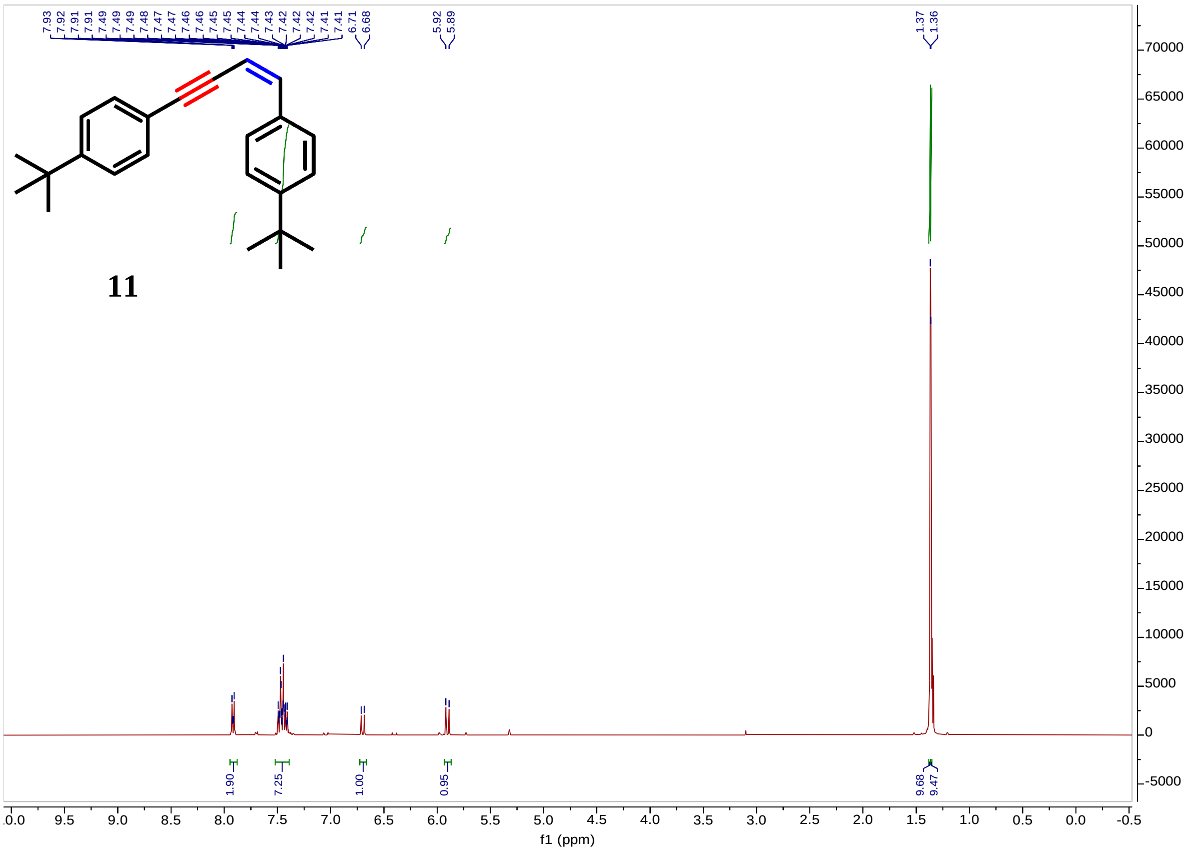
7.93 7.92 7.91 7.91 7.49 7.49 7.49 7.48 7.47 7.47 7.46 7.46 7.45 7.45 7.44 7.44 7.43 7.42 7.42 7.42 7.42 7.41 6.71 6.68
5.92 5.89
1.37 1.36
11
1.90 7.25 1.00 0.95 9.68 9.47
f1 (ppm)

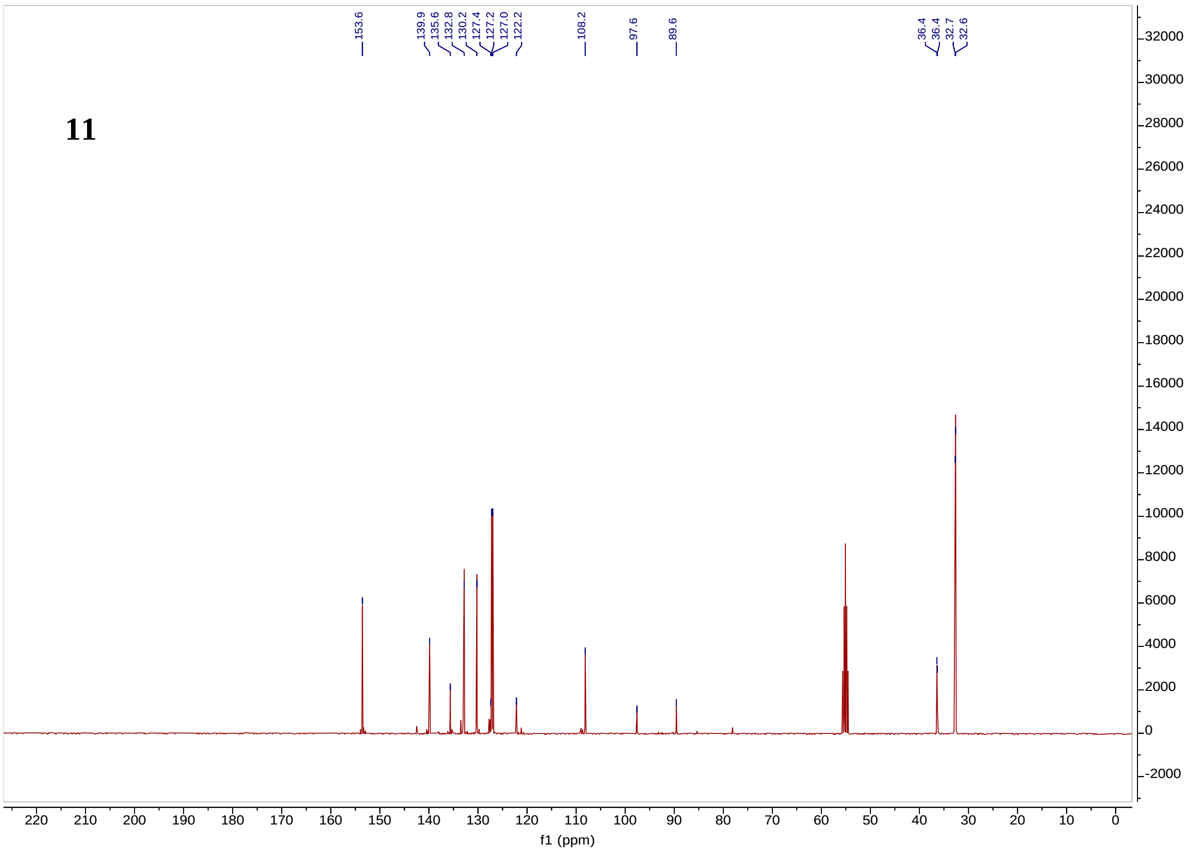
153.6
139.9 135.6 132.8 130.2 127.4 127.2 127.0 122.2
108.2
97.6
89.6
36.4 36.4 32.7 32.6
11
f1 (ppm)

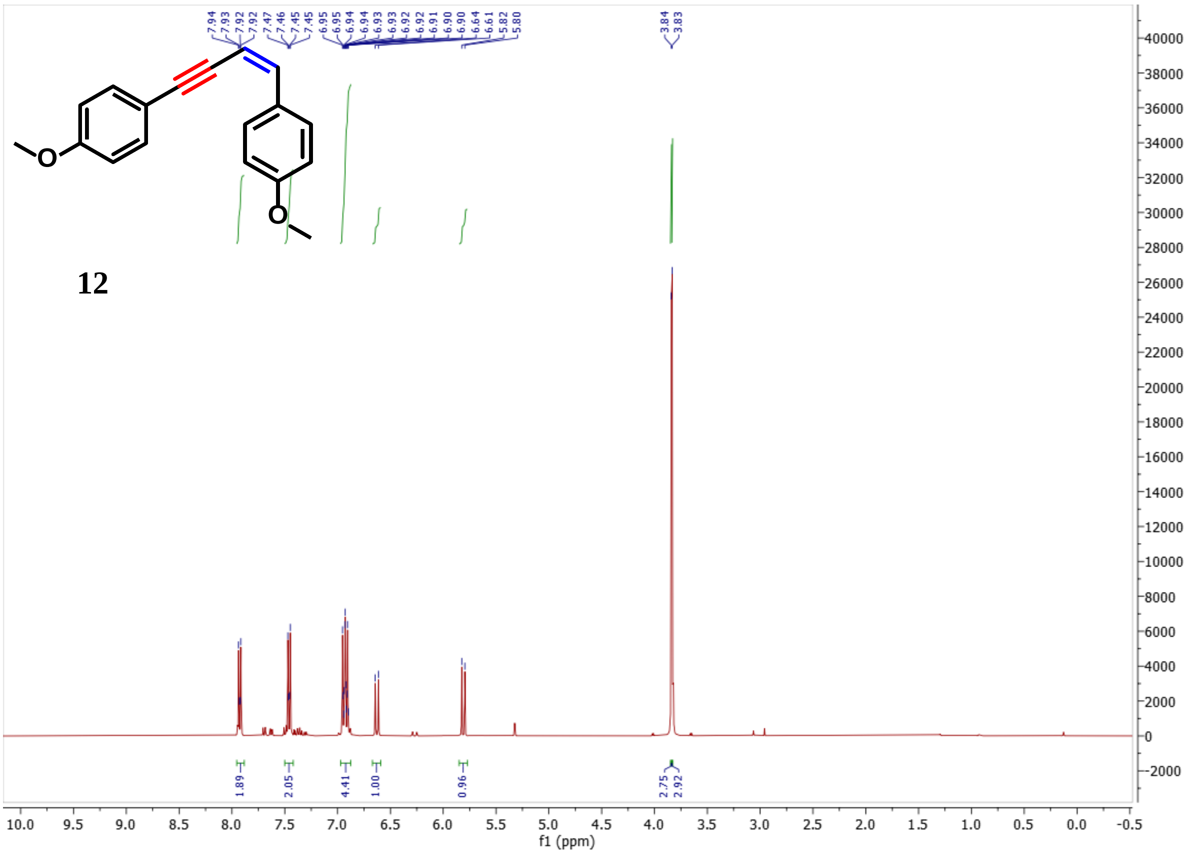
7.94 7.93 7.92 7.92 7.47 7.46 7.45 7.45 6.95 6.95 6.94 6.94 6.93 6.93 6.92 6.92 6.91 6.90 6.90 6.64 6.61 5.82 5.80
3.84 3.83
12
1.89 2.05 4.41 1.00 0.96 2.75 2.92
f1 (ppm)

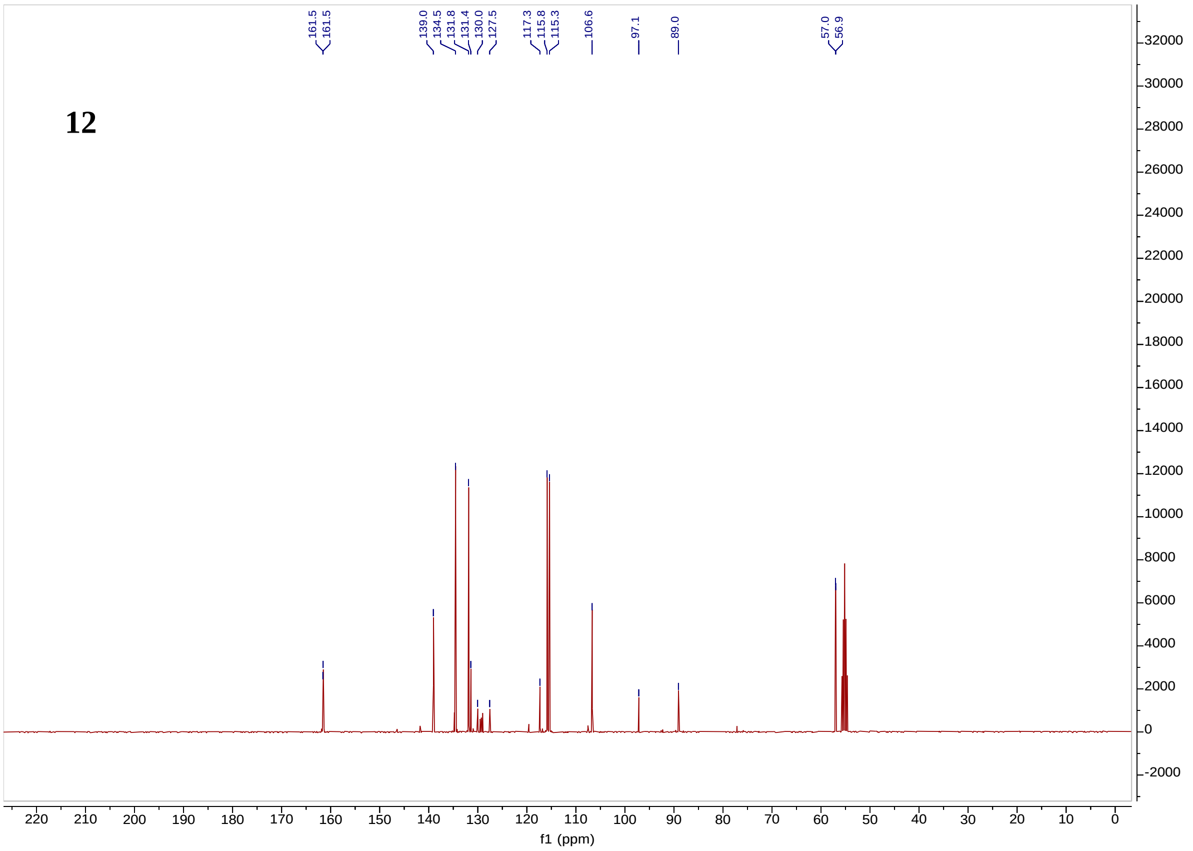
161.5 161.5
139.0 134.5 131.8 131.4 130.0 127.5
117.3 115.8 115.3
106.6
97.1
89.0
57.0 56.9
12
f1 (ppm)

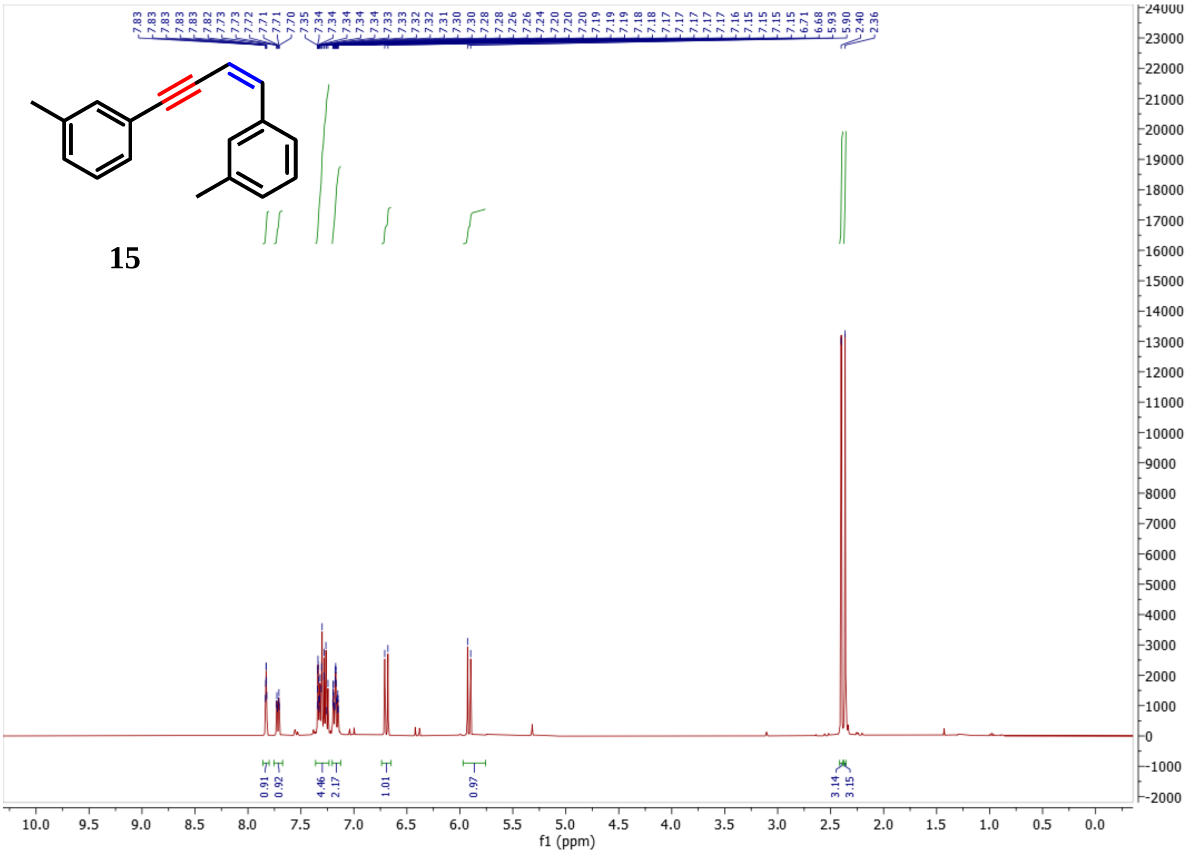
15
f1 (ppm)

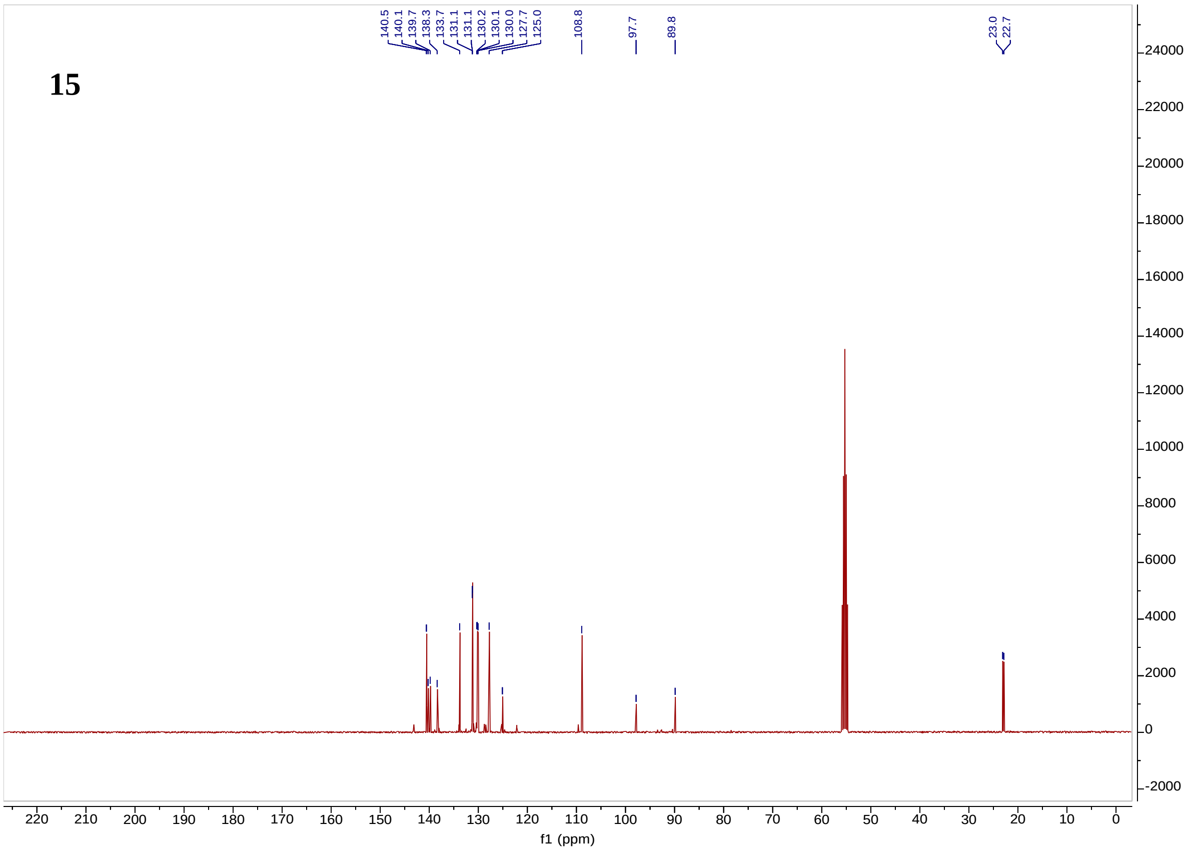
15
f1 (ppm)

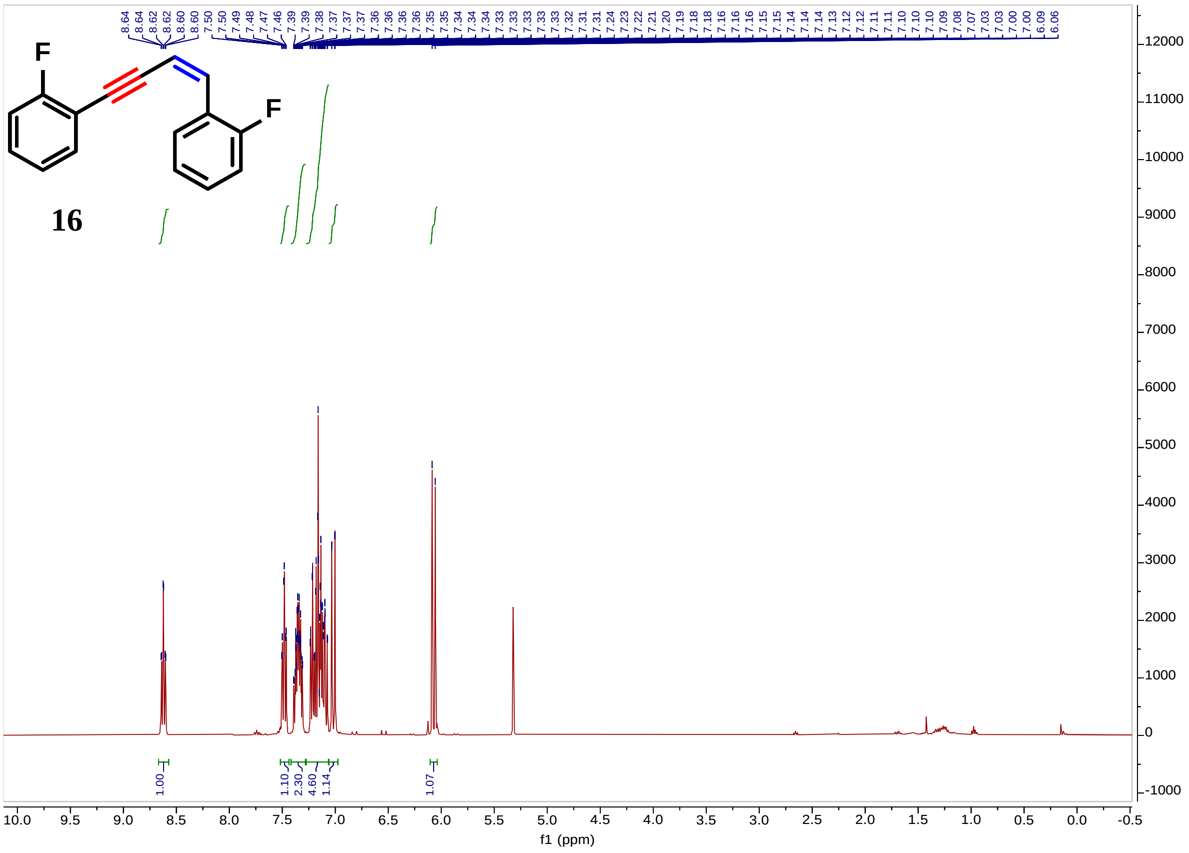

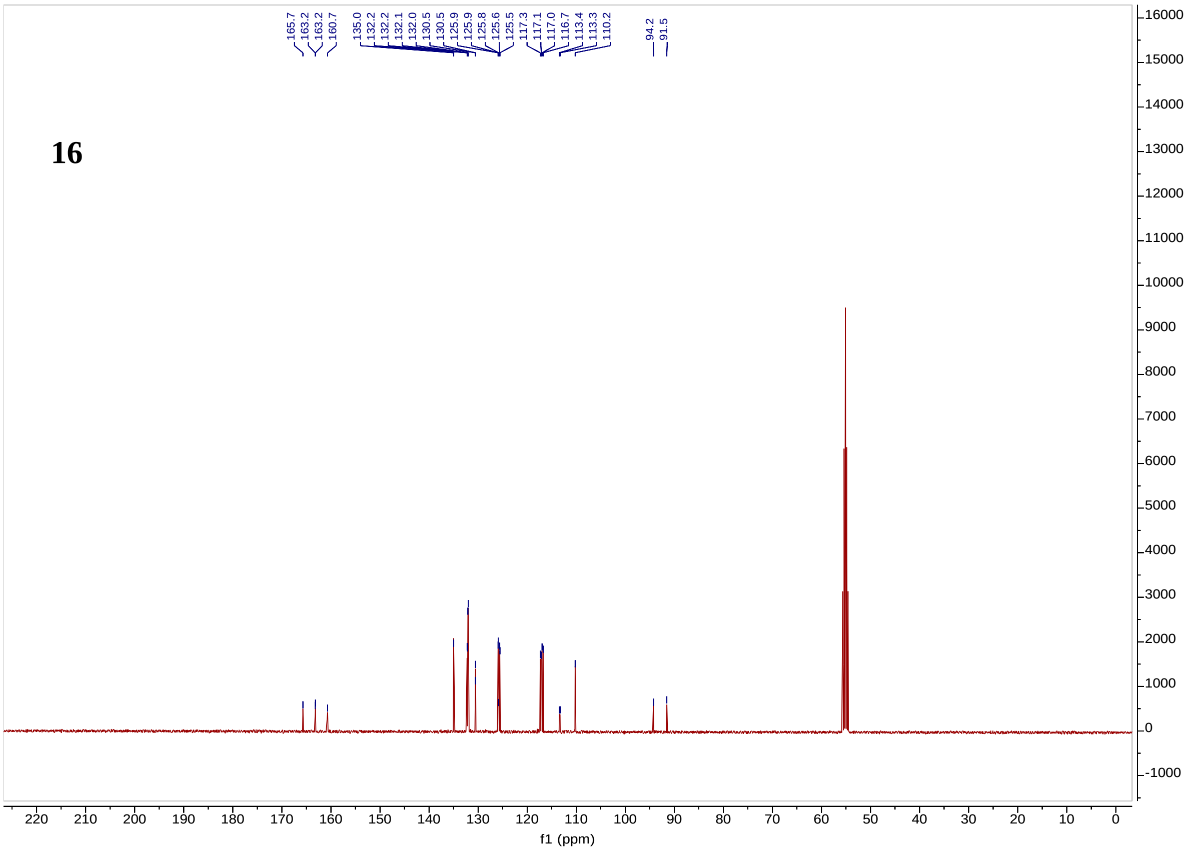

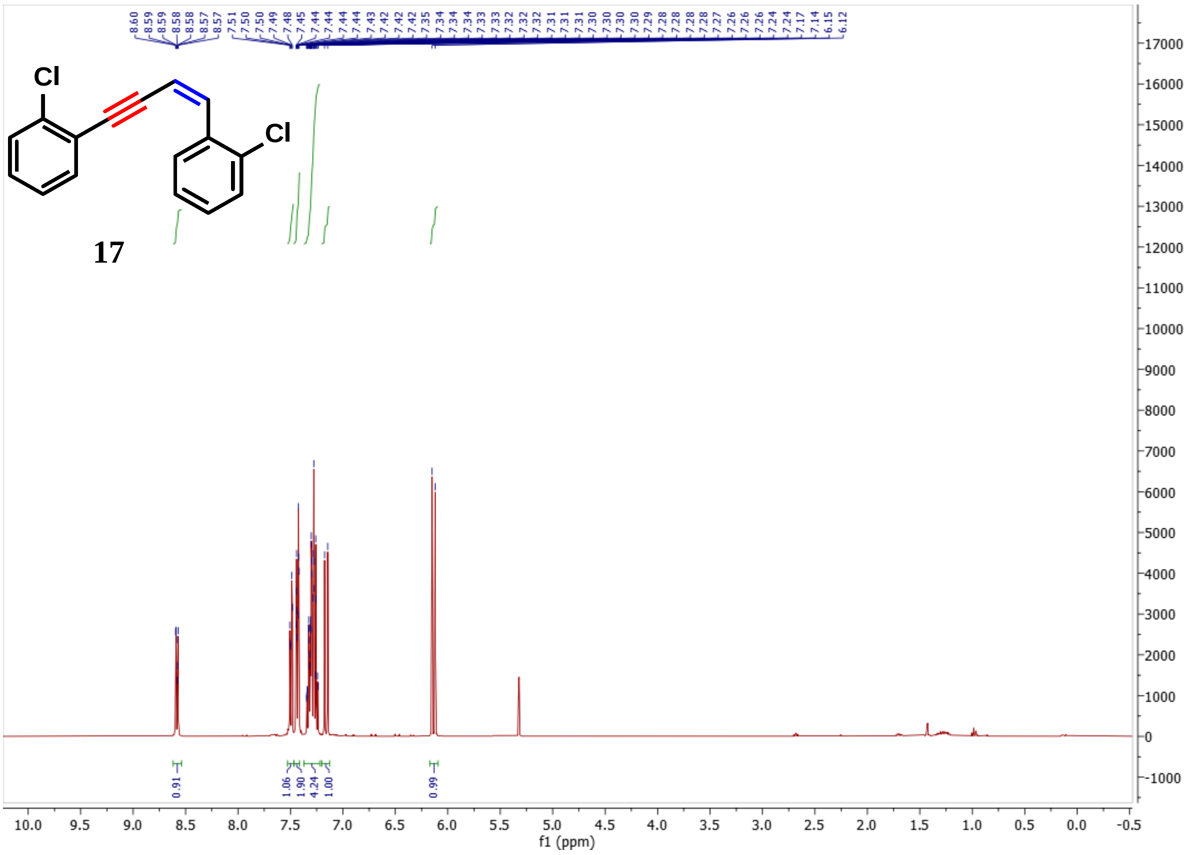

Cl
Cl
17
8.60
8.59
8.59
8.58
8.58
8.57
8.57
7.51
7.50
7.50
7.49
7.48
7.45
7.44
7.44
7.44
7.44
7.43
7.42
7.42
7.35
7.34
7.34
7.34
7.34
7.33
7.33
7.32
7.32
7.32
7.31
7.31
7.30
7.30
7.30
7.30
7.29
7.28
7.28
7.28
7.28
7.27
7.26
7.26
7.26
7.24
7.24
7.17
7.14
6.15
6.12
0.91
1.06
1.90
4.24
1.00
0.99
f1 (ppm)

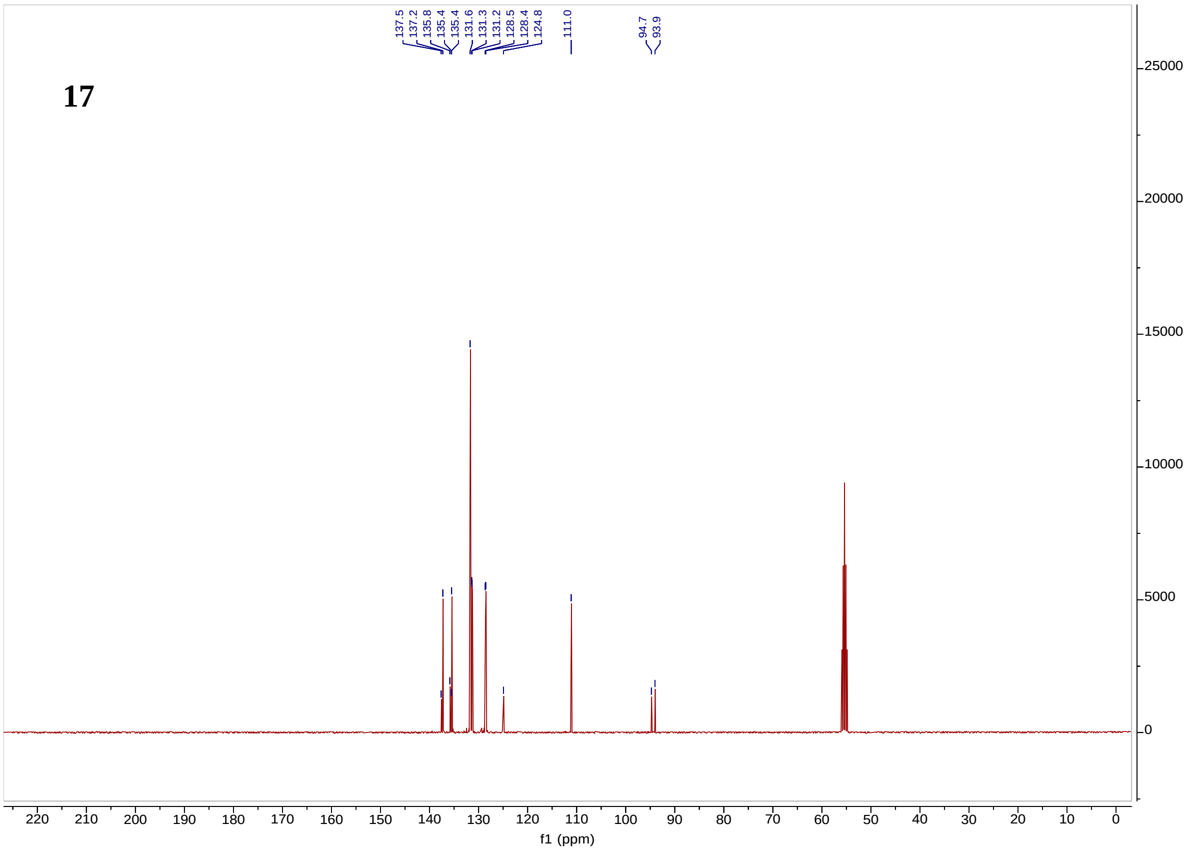

17
137.5
137.2
135.8
135.4
135.4
131.6
131.3
131.2
128.5
128.4
124.8
111.0
94.7
93.9
f1 (ppm)

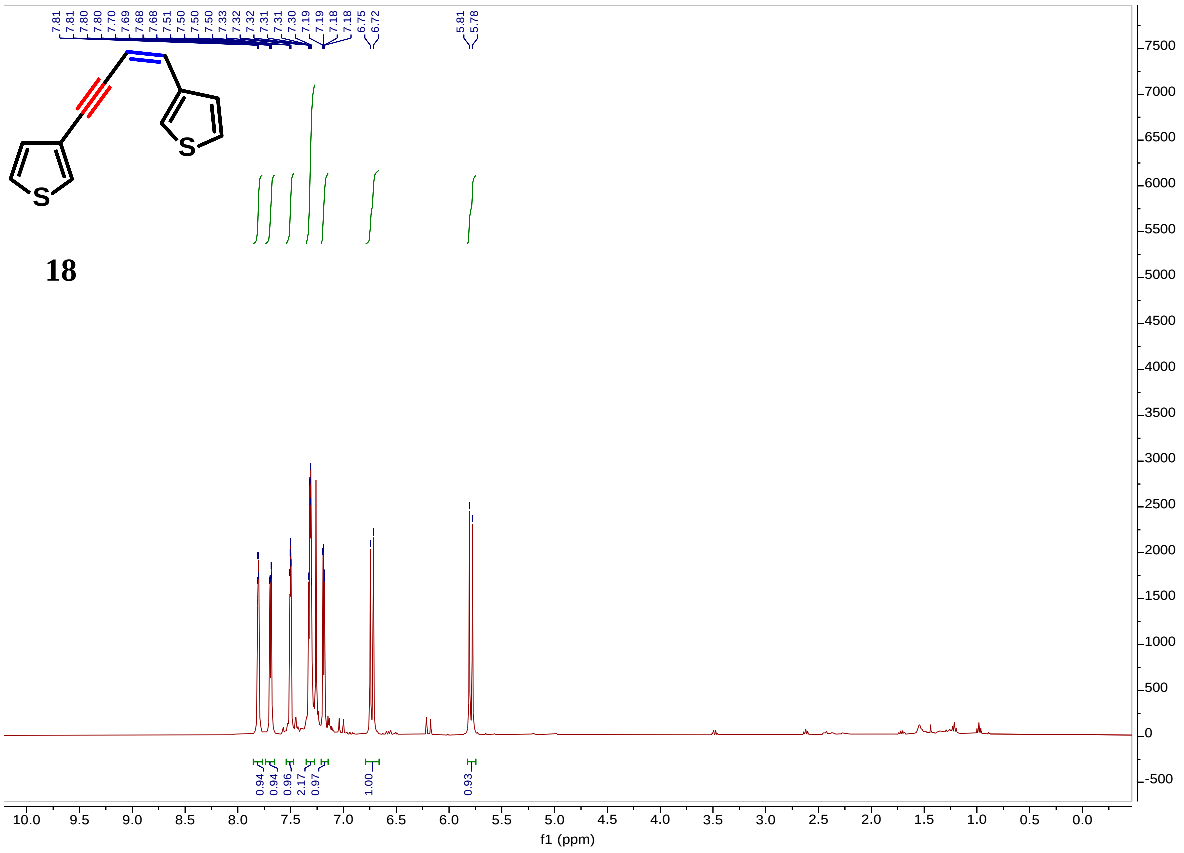

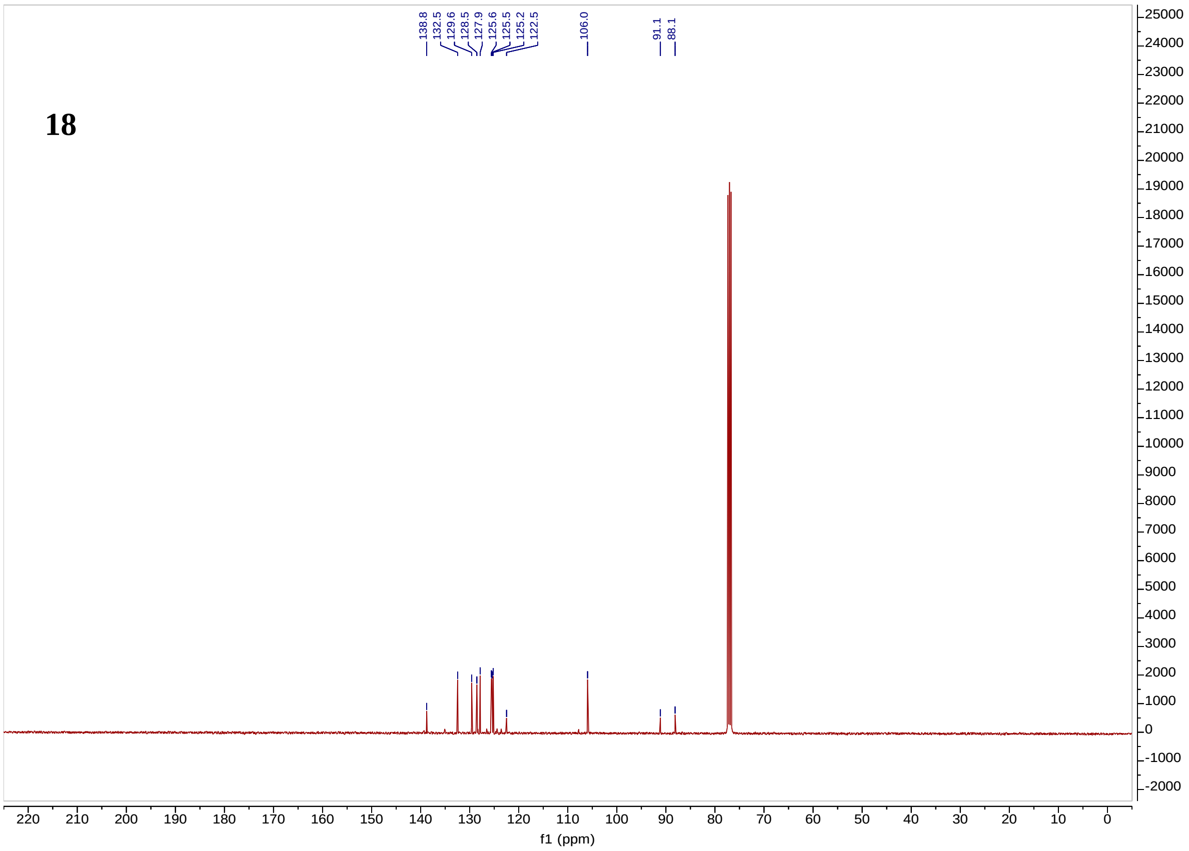

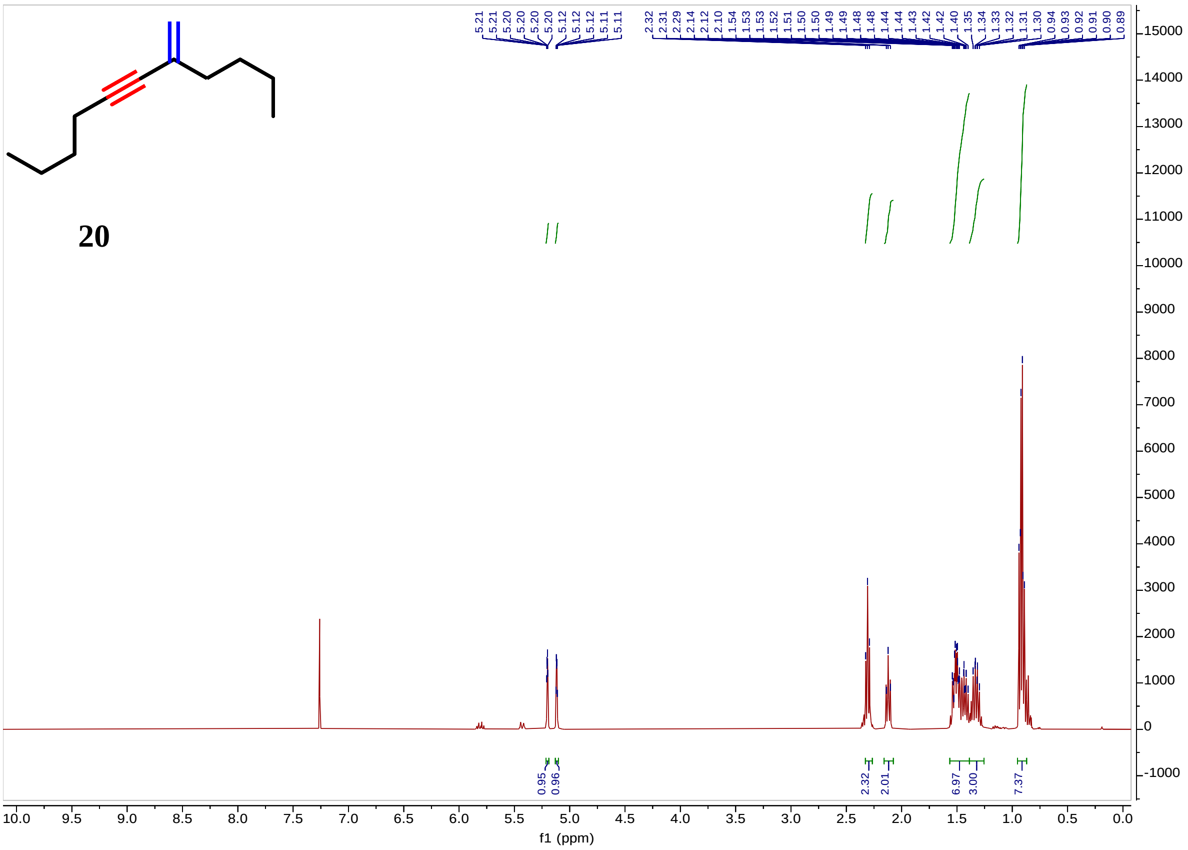

5.21
5.21
5.20
5.20
5.20
5.20
5.12
5.12
5.12
5.11
5.11
2.32
2.31
2.29
2.14
2.12
2.10
1.54
1.53
1.53
1.52
1.51
1.50
1.50
1.49
1.49
1.48
1.48
1.44
1.44
1.43
1.42
1.42
1.40
1.35
1.34
1.33
1.32
1.31
1.30
0.94
0.93
0.92
0.91
0.90
0.89
0.95
0.96
2.32
2.01
6.97
3.00
7.37
f1 (ppm)
20

20

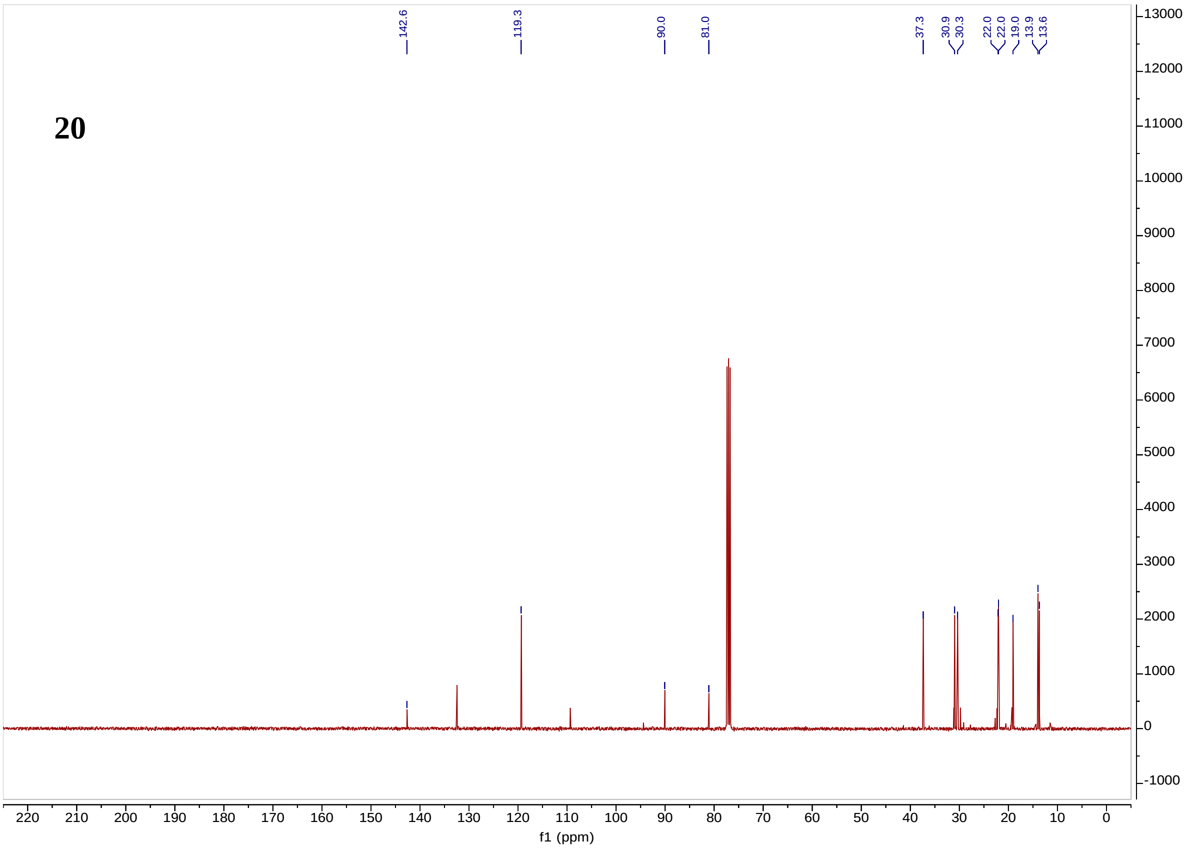

142.6
119.3
90.0
81.0
37.3
30.9
30.3
22.0
22.0
19.0
13.9
13.6
f1 (ppm)

21

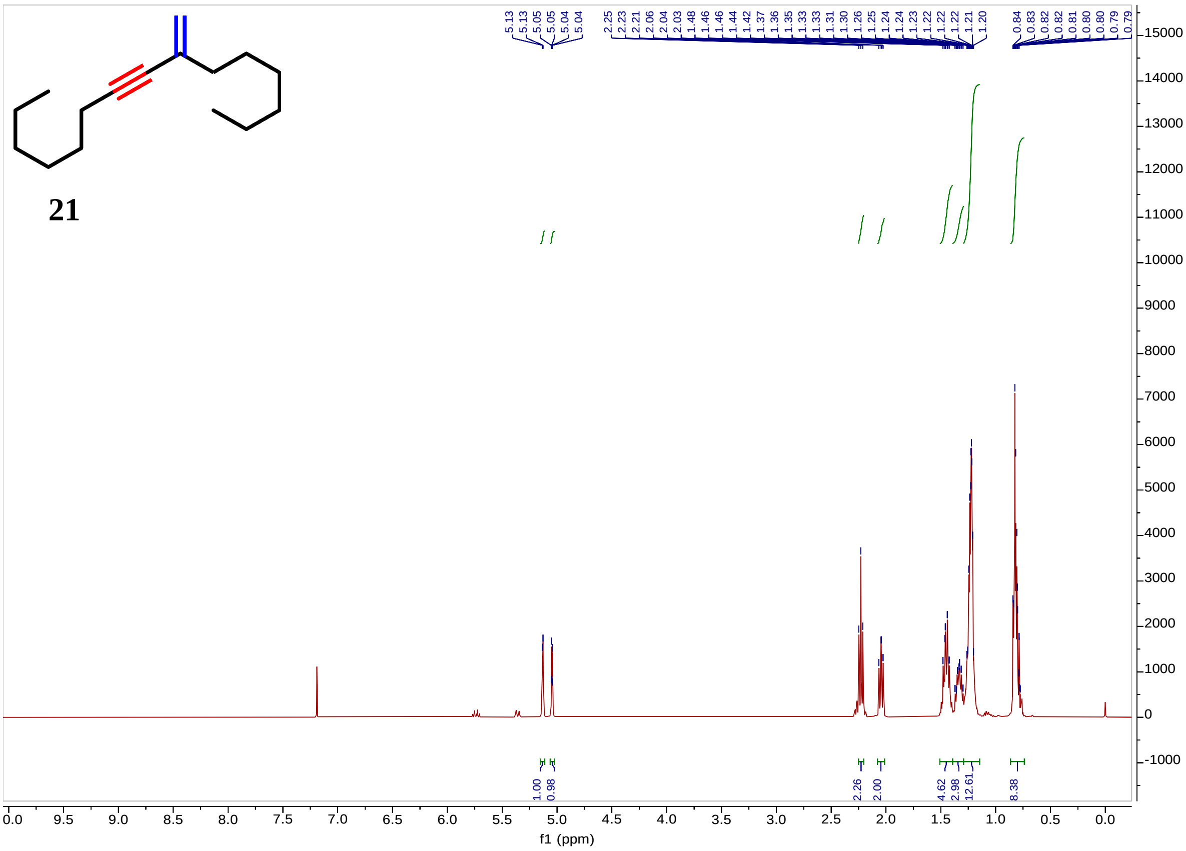

5.13
5.13
5.05
5.05
5.04
5.04
2.25
2.23
2.21
2.06
2.04
2.03
1.48
1.46
1.46
1.44
1.42
1.37
1.36
1.35
1.33
1.33
1.31
1.30
1.26
1.25
1.24
1.24
1.23
1.22
1.22
1.22
1.21
1.20
0.84
0.83
0.82
0.82
0.81
0.80
0.80
0.79
1.00
0.98
2.26
2.00
4.62
2.98
12.61
8.38
f1 (ppm)

21

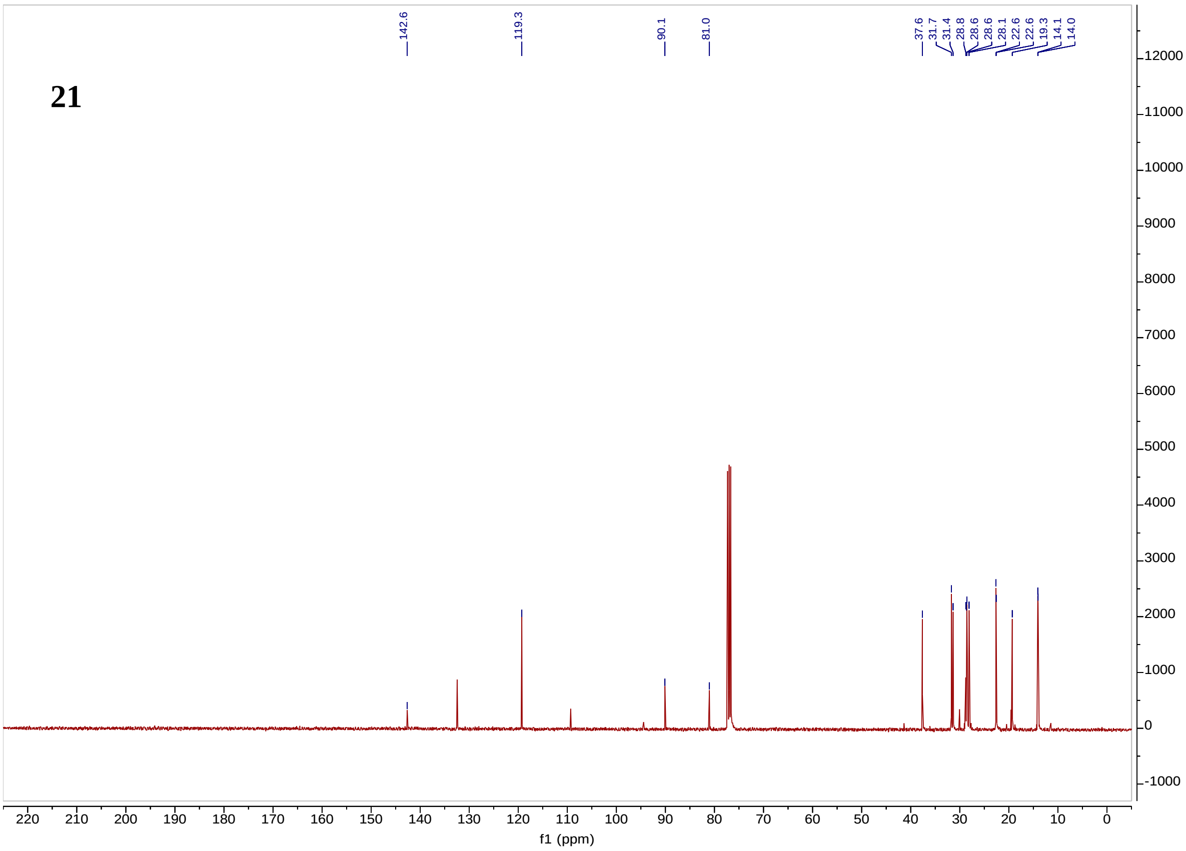

142.6
119.3
90.1
81.0
37.6
31.7
31.4
28.8
28.6
28.6
28.1
22.6
22.6
19.3
14.1
14.0
f1 (ppm)

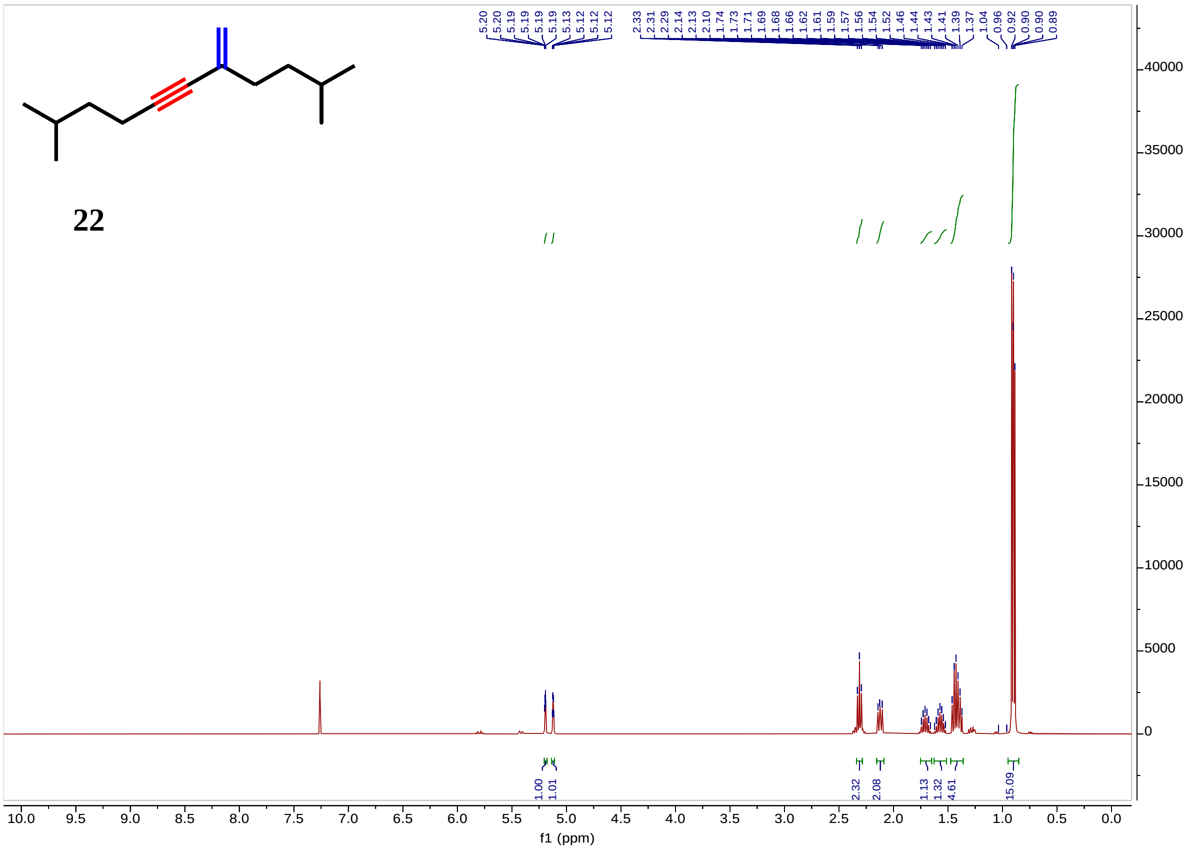

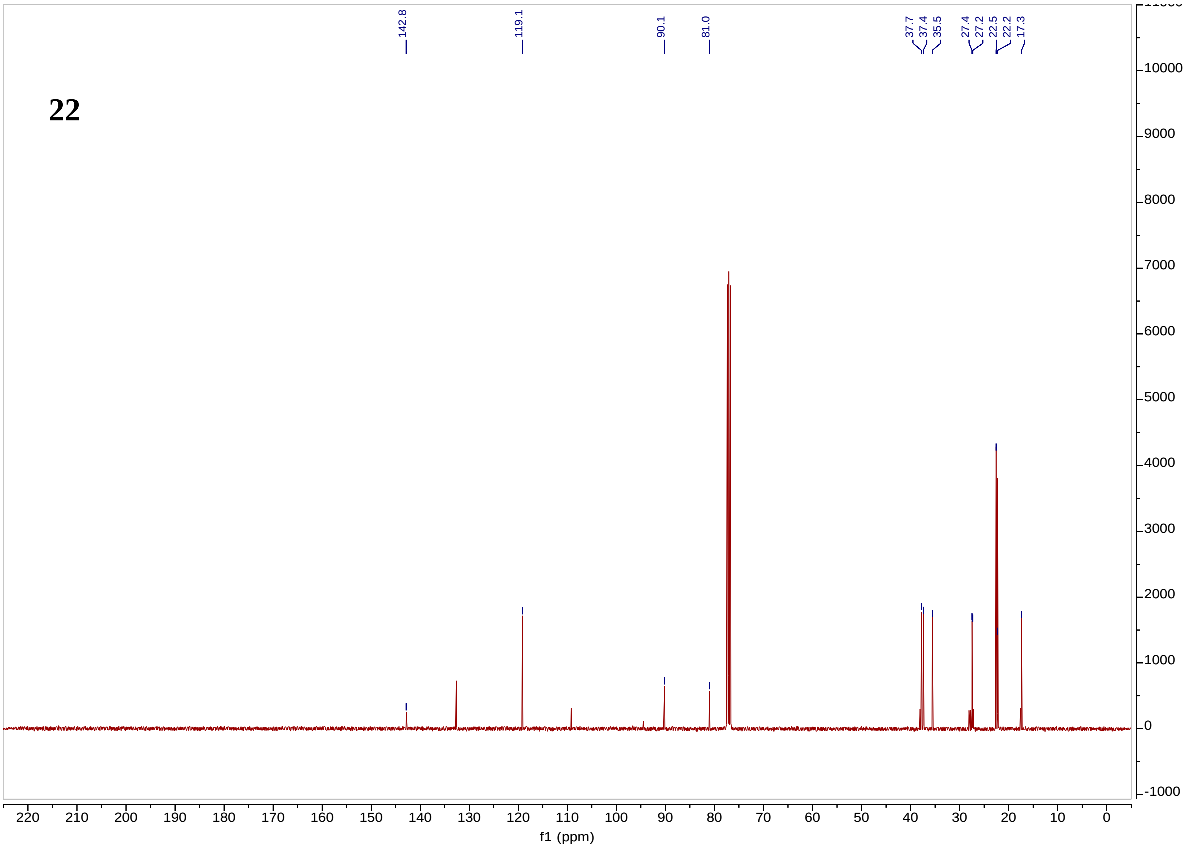

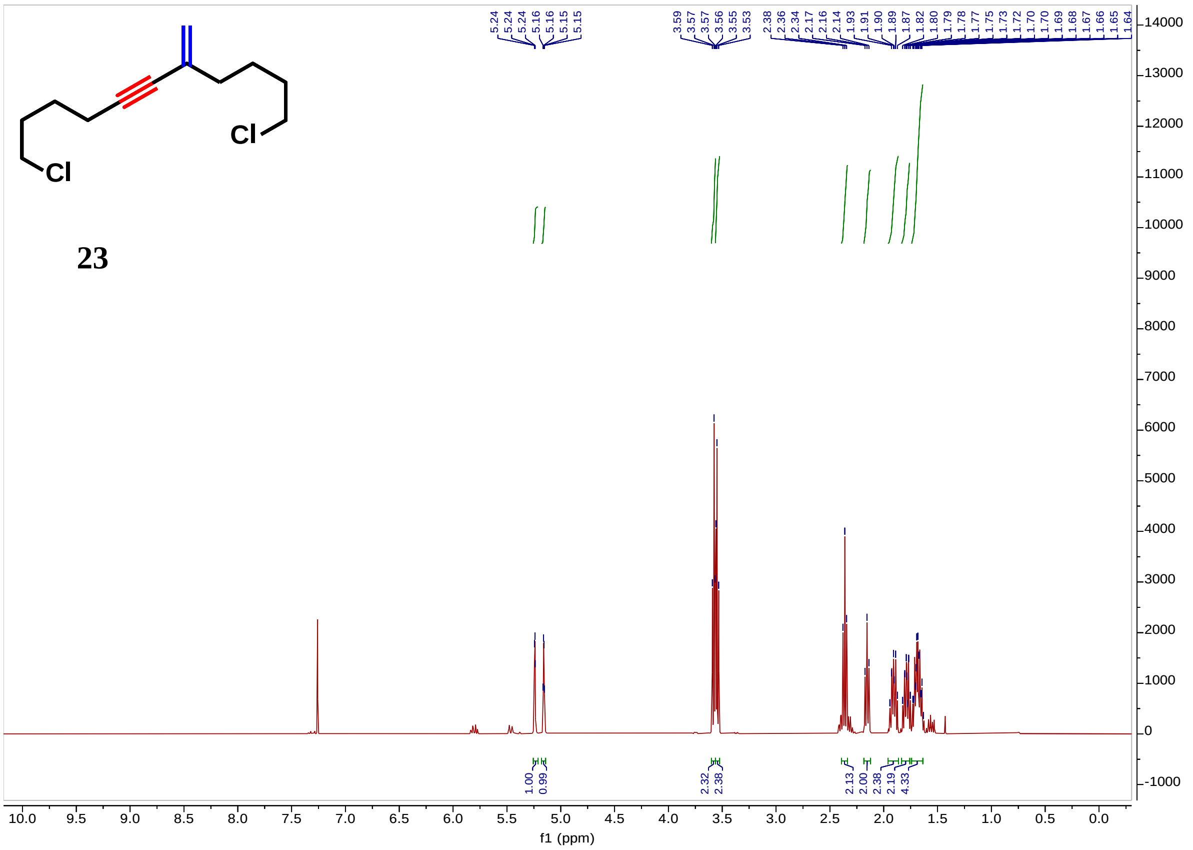
Cl
Cl
23
f1 (ppm)

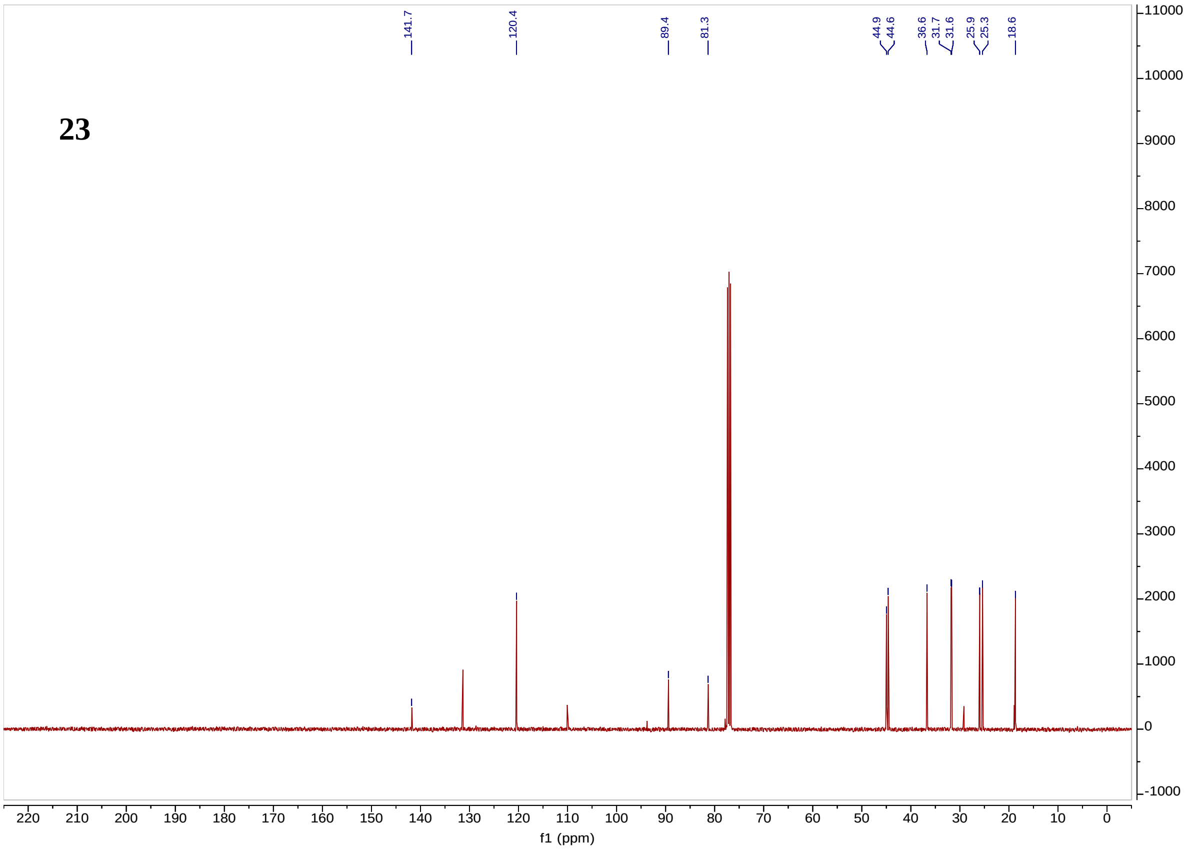
23
f1 (ppm)

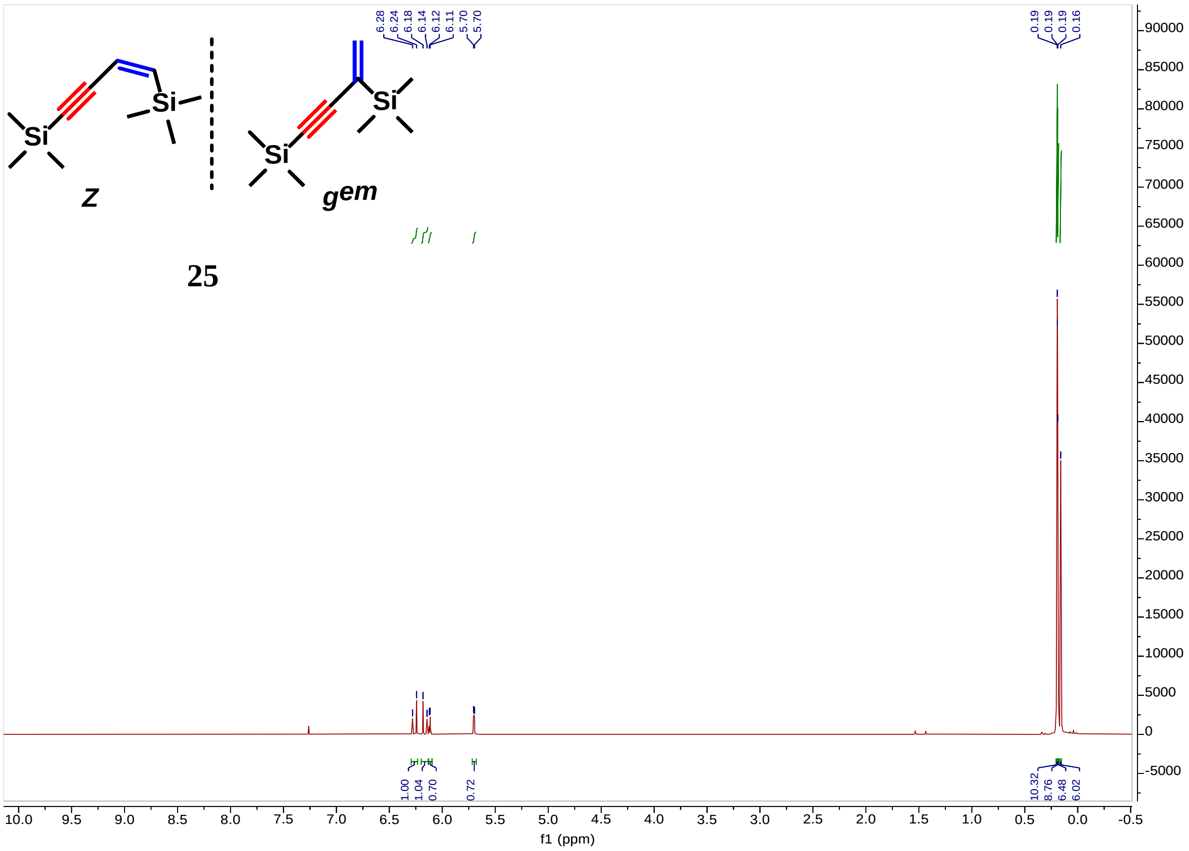
Si
Si
Z
Si
Si
gem
25
f1 (ppm)

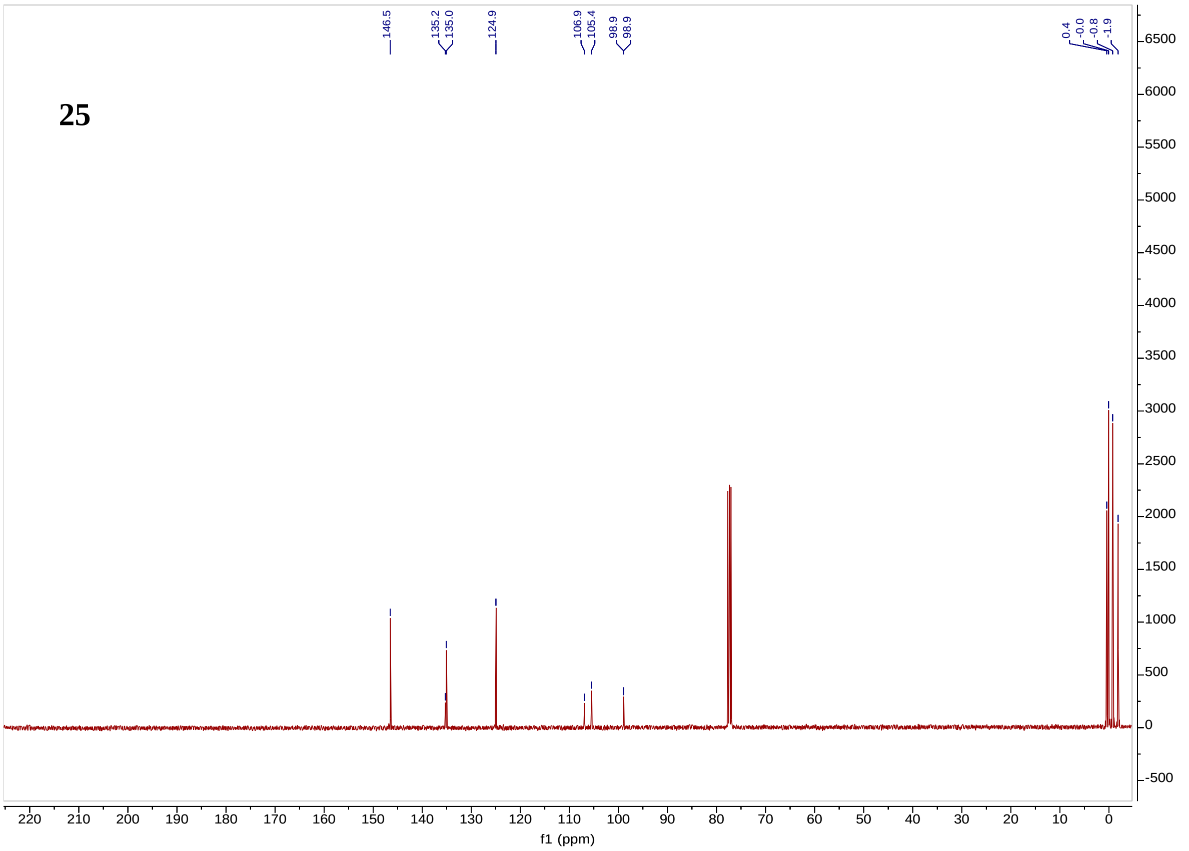
25
f1 (ppm)

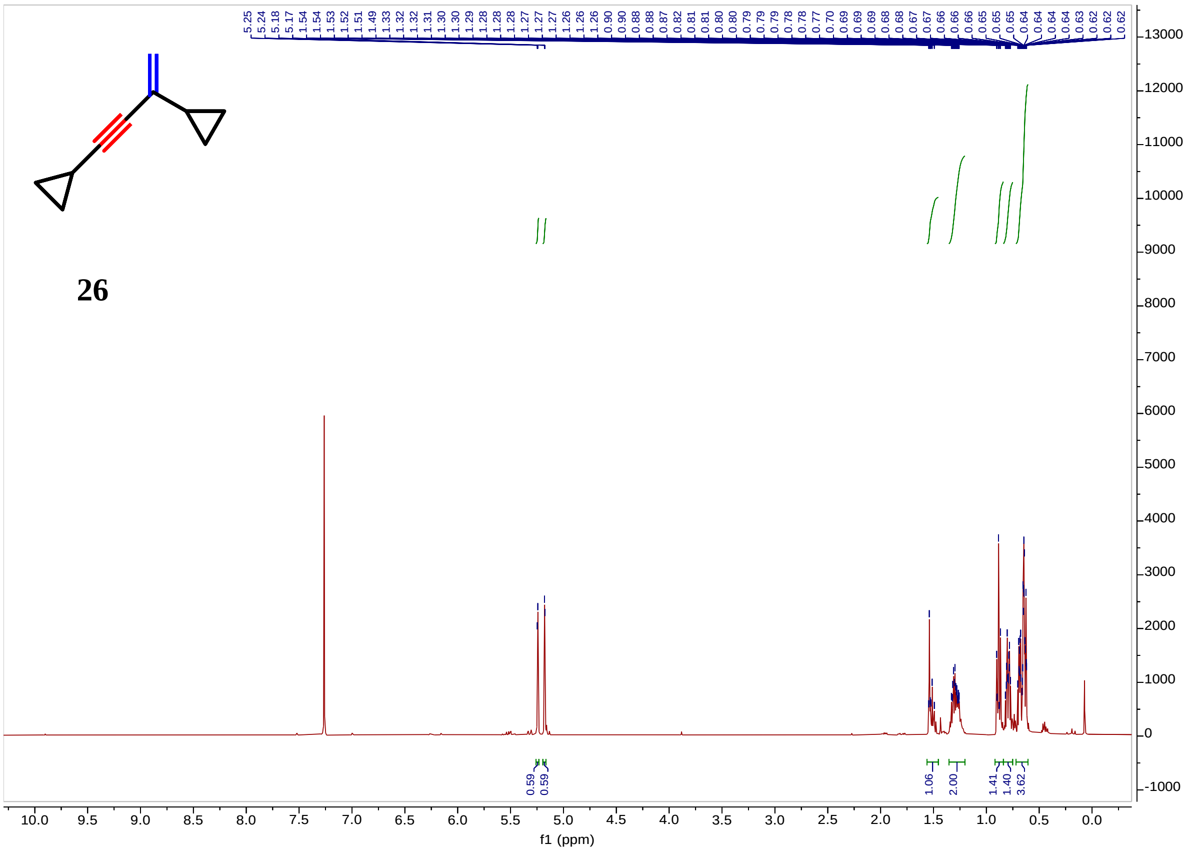

5.25
5.24
5.18
5.17
1.54
1.54
1.53
1.52
1.51
1.49
1.33
1.32
1.32
1.31
1.30
1.30
1.29
1.28
1.28
1.28
1.27
1.27
1.27
1.26
1.26
0.90
0.90
0.88
0.88
0.87
0.82
0.81
0.81
0.80
0.80
0.79
0.79
0.79
0.78
0.78
0.77
0.70
0.69
0.69
0.69
0.68
0.68
0.67
0.67
0.66
0.66
0.66
0.65
0.65
0.65
0.64
0.64
0.64
0.64
0.63
0.62
0.62
0.62
26
0.59
0.59
1.06
2.00
1.41
1.40
3.62
f1 (ppm)

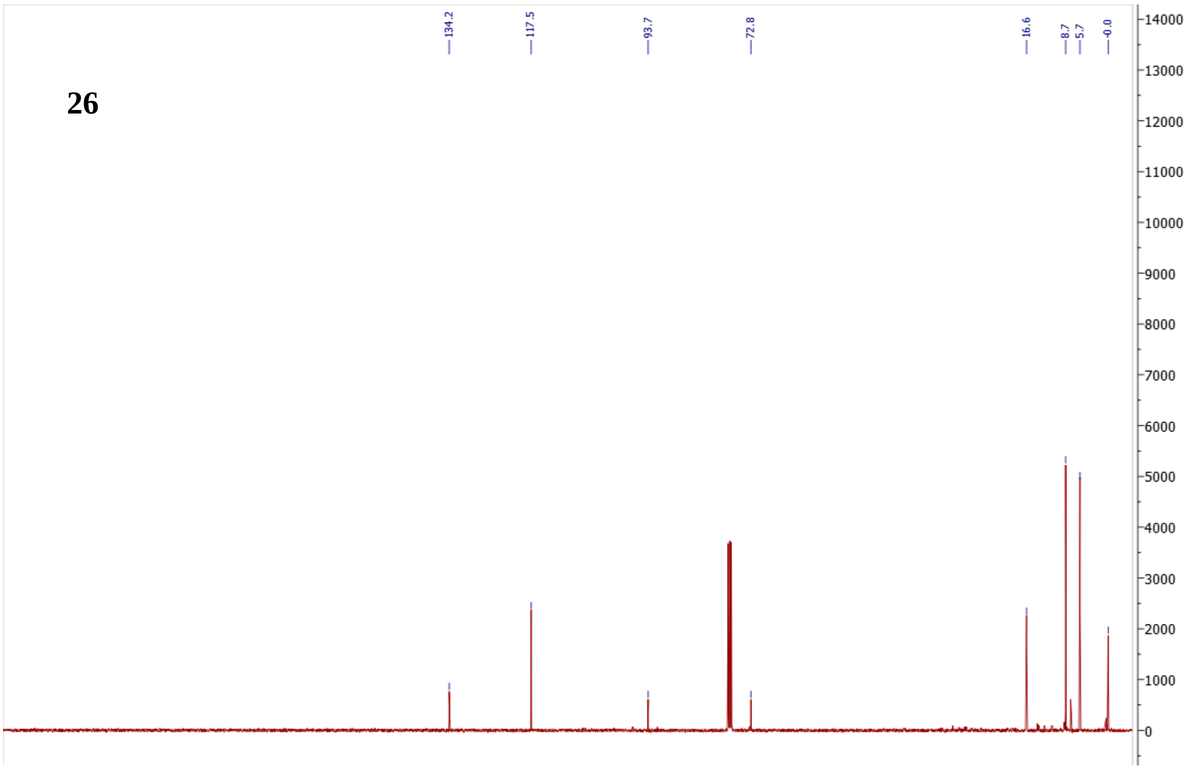

134.2
117.5
93.7
72.8
16.6
8.7
5.7
-0.0
26

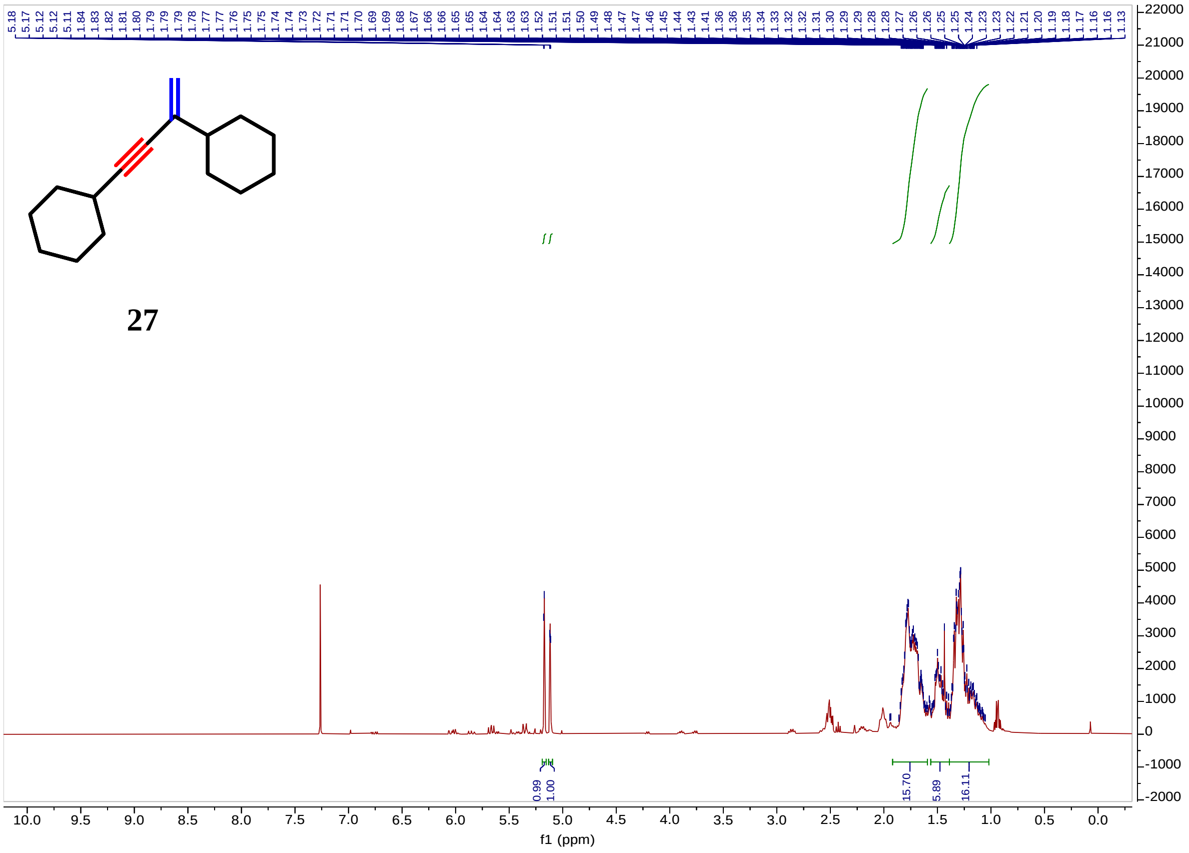
5.18 5.17 5.12 5.12 5.11 1.84 1.83 1.82 1.81 1.80 1.79 1.79 1.79 1.78 1.77 1.77 1.76 1.75 1.75 1.74 1.73 1.72 1.71 1.71 1.70 1.69 1.69 1.68 1.67 1.66 1.66 1.65 1.65 1.64 1.63 1.63 1.52 1.51 1.51 1.50 1.49 1.48 1.47 1.47 1.46 1.45 1.44 1.43 1.41 1.36 1.36 1.35 1.34 1.33 1.32 1.32 1.31 1.30 1.29 1.29 1.28 1.28 1.27 1.26 1.26 1.25 1.25 1.24 1.23 1.23 1.22 1.21 1.20 1.19 1.18 1.17 1.16 1.16 1.13
27
0.99
1.00
15.70
5.89
16.11
f1 (ppm)

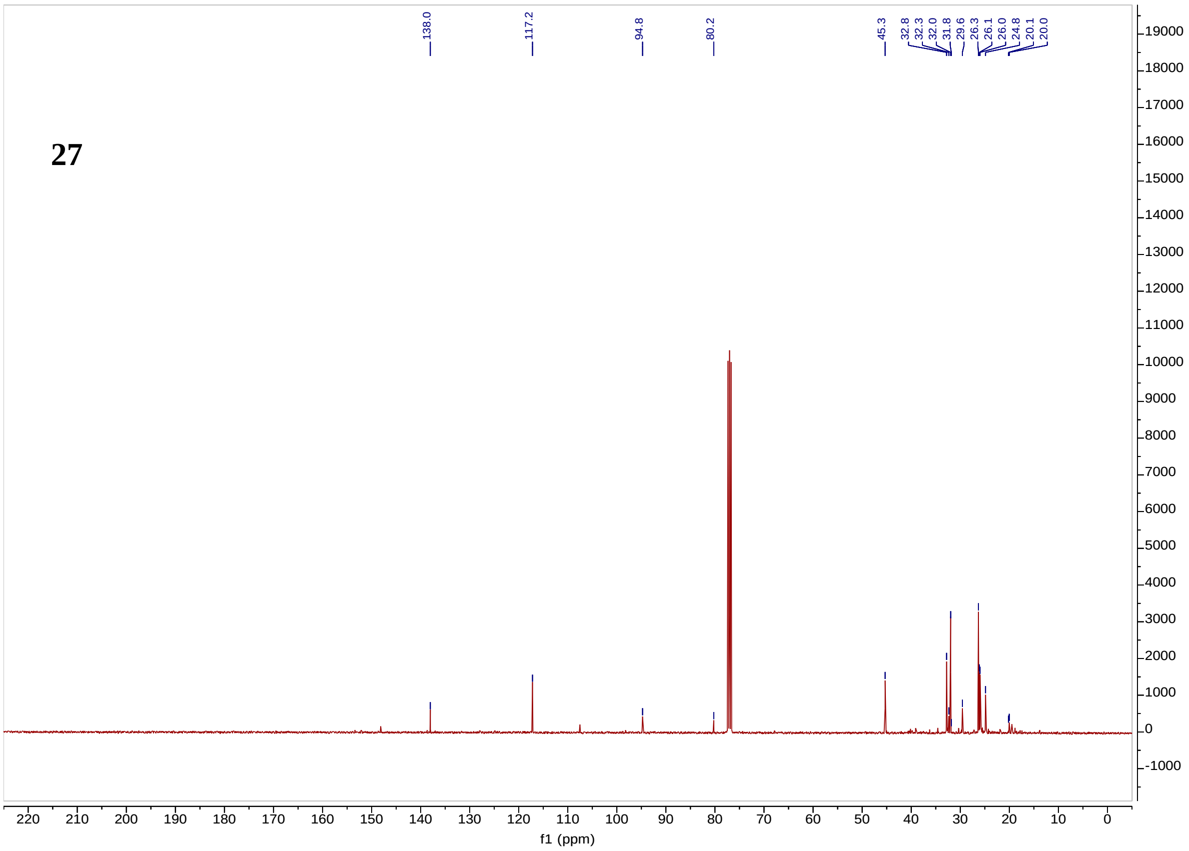
138.0
117.2
94.8
80.2
45.3
32.8
32.3
32.0
31.8
29.6
26.3
26.1
26.0
24.8
20.1
20.0
27
f1 (ppm)

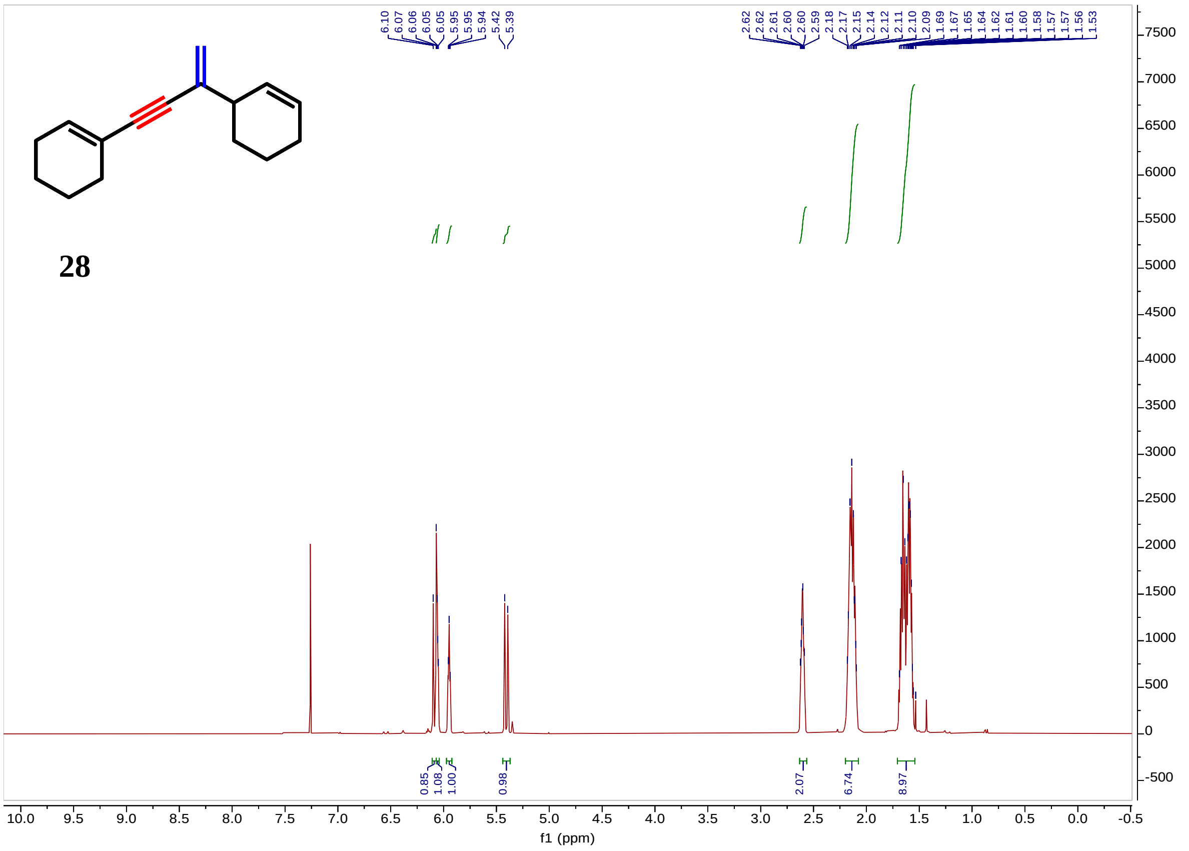
28
6.10 6.07 6.06 6.05 6.05 5.95 5.95 5.94 5.42 5.39
2.62 2.62 2.61 2.60 2.60 2.59 2.18 2.17 2.15 2.14 2.12 2.11 2.10 2.09 1.69 1.67 1.65 1.64 1.62 1.61 1.60 1.58 1.57 1.57 1.56 1.53
0.85 1.08 1.00 0.98 2.07 6.74 8.97
f1 (ppm)

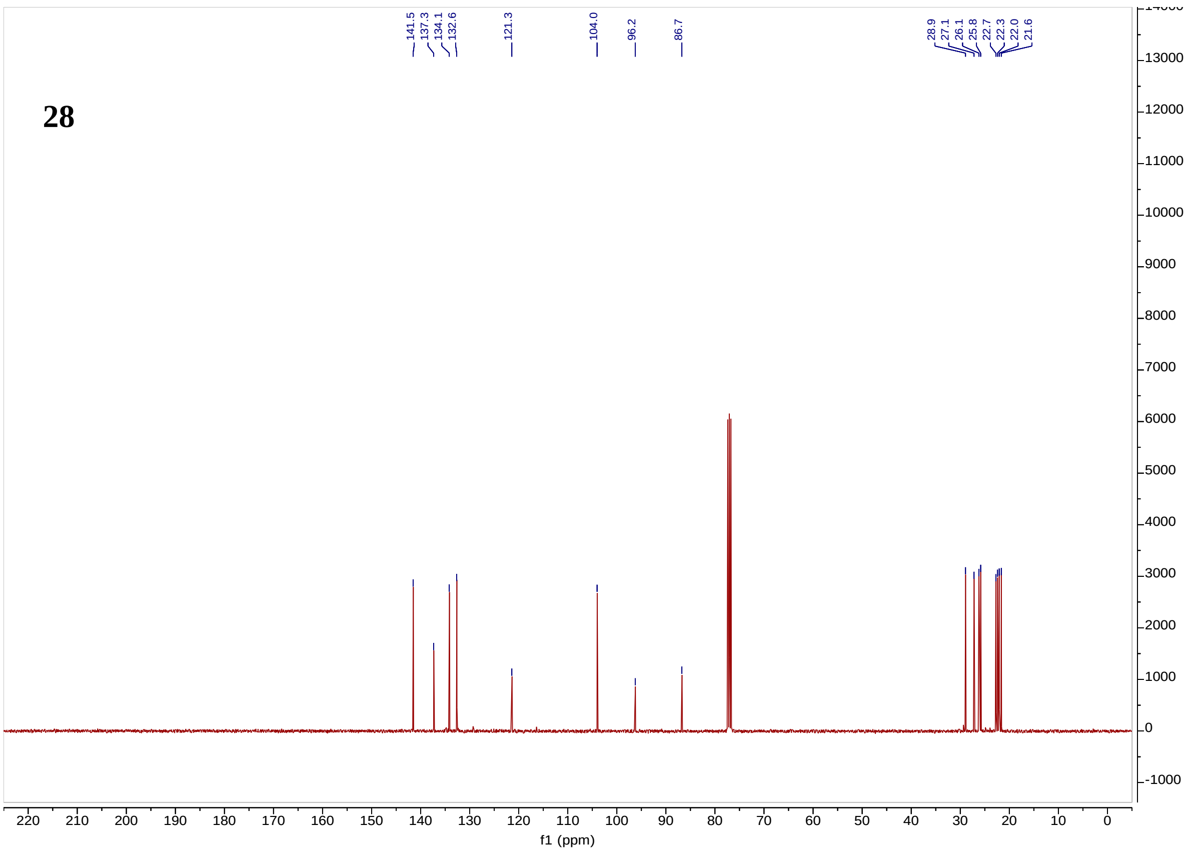
28
141.5 137.3 134.1 132.6 121.3 104.0 96.2 86.7
28.9 27.1 26.1 25.8 22.7 22.3 22.0 21.6
f1 (ppm)

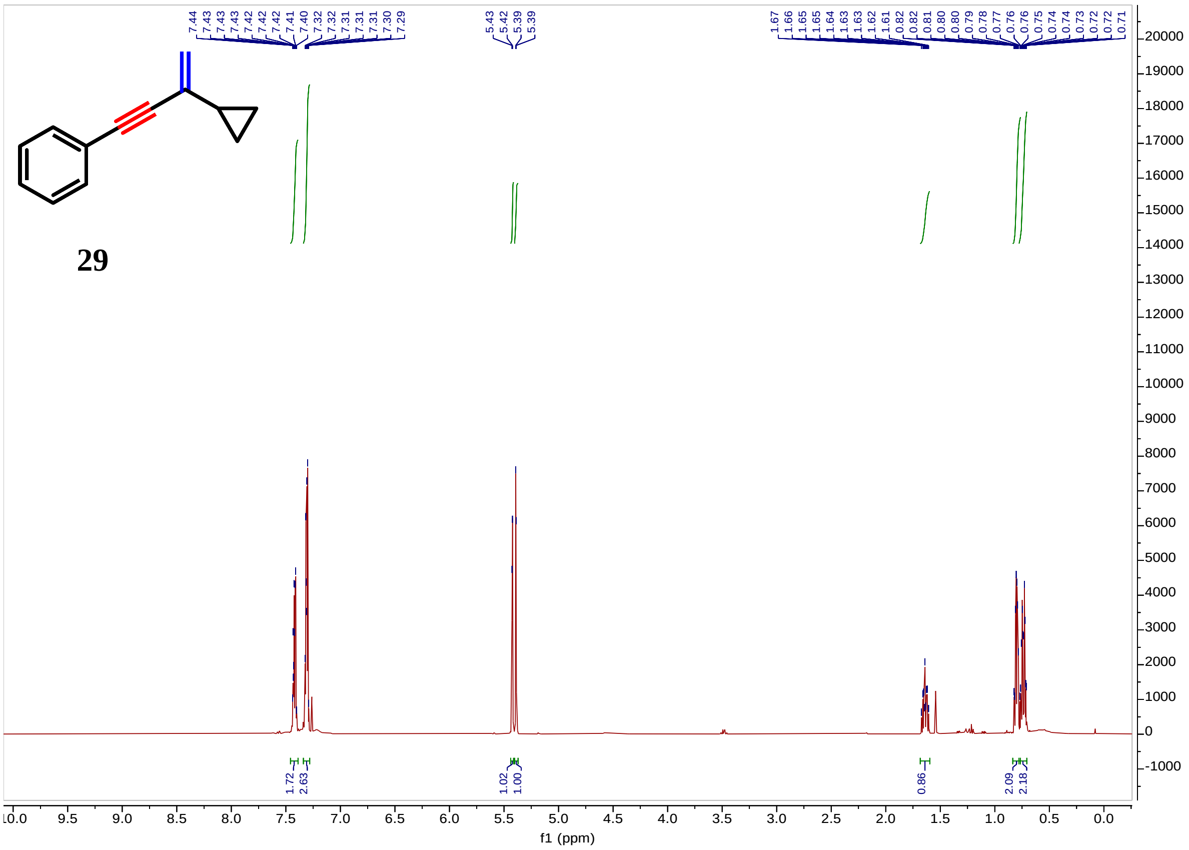
29
f1 (ppm)

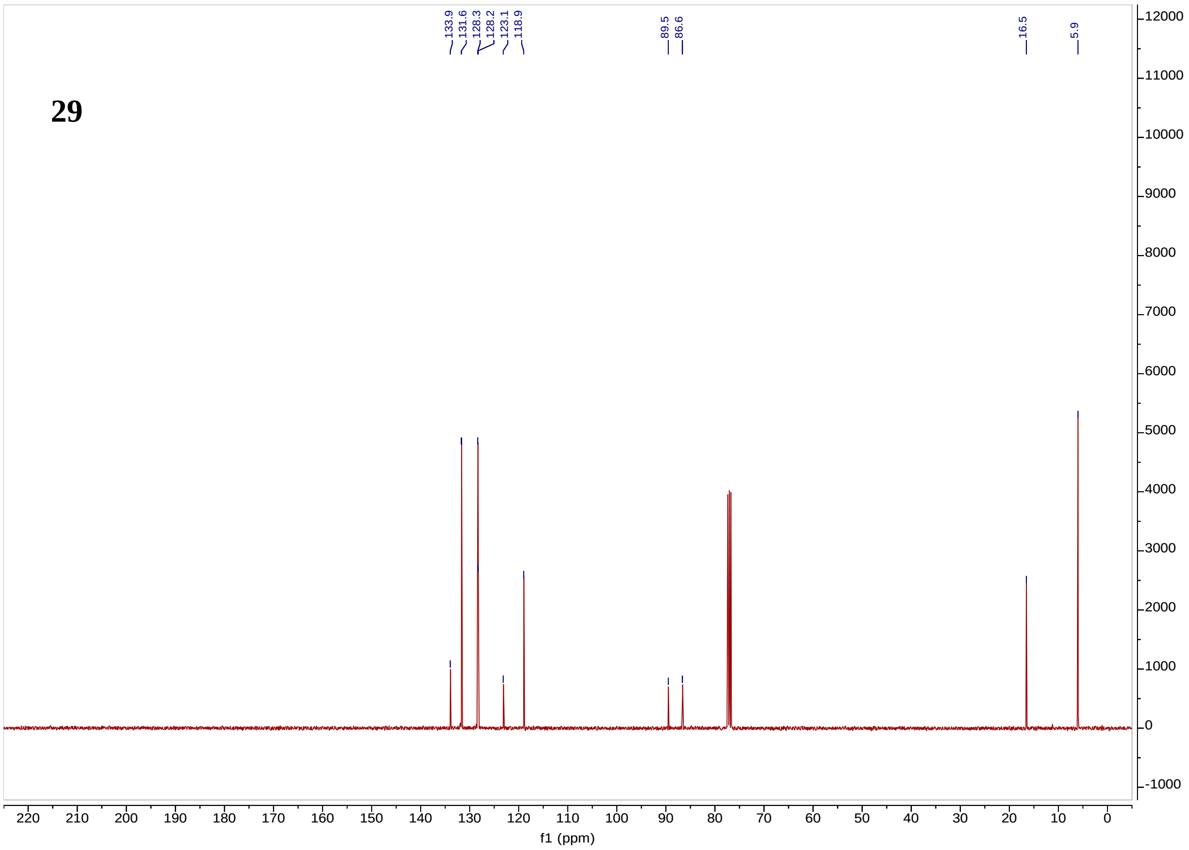
29
f1 (ppm)

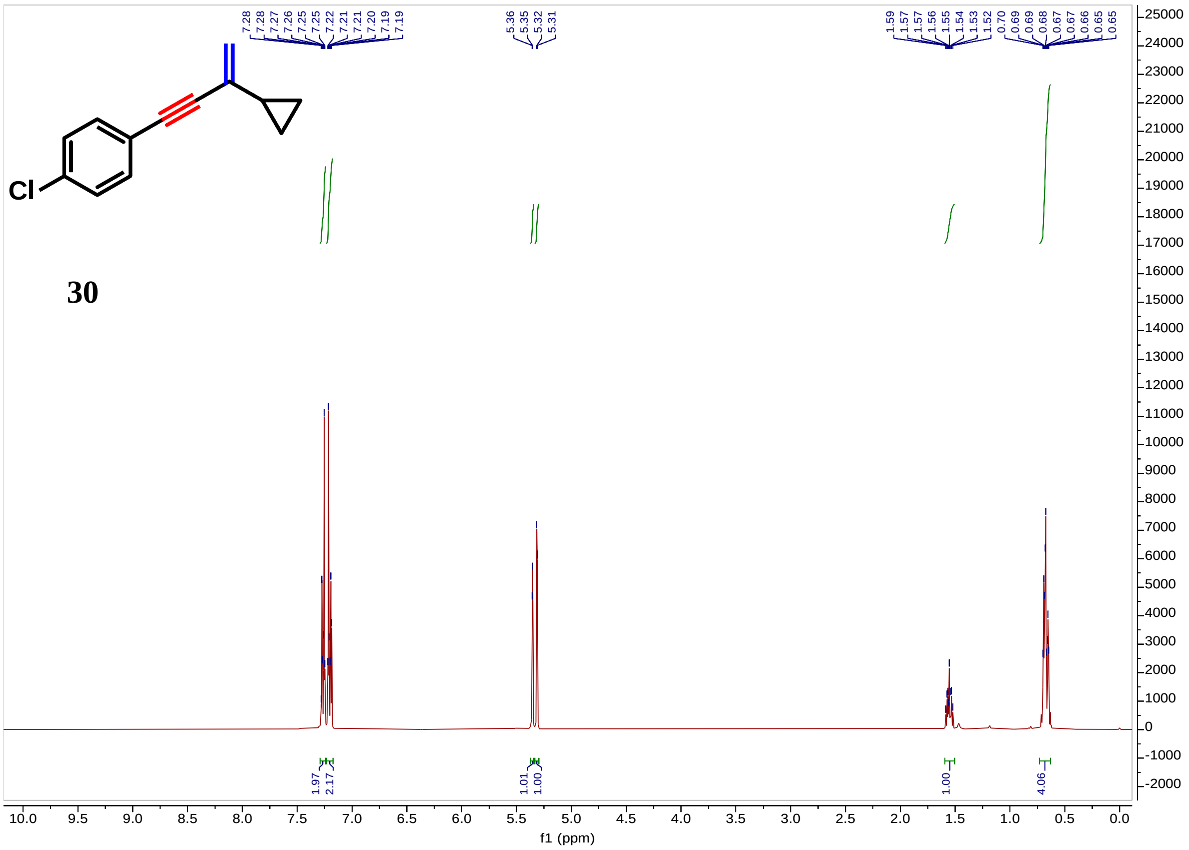

7.28 7.28 7.27 7.26 7.25 7.25 7.22 7.21 7.21 7.20 7.19 7.19
5.36 5.35 5.32 5.31
1.59 1.57 1.57 1.56 1.55 1.55 1.54 1.53 1.52
0.70 0.69 0.69 0.68 0.67 0.67 0.66 0.65 0.65
Cl
30
1.97 2.17 1.01 1.00 1.00 4.06
f1 (ppm)

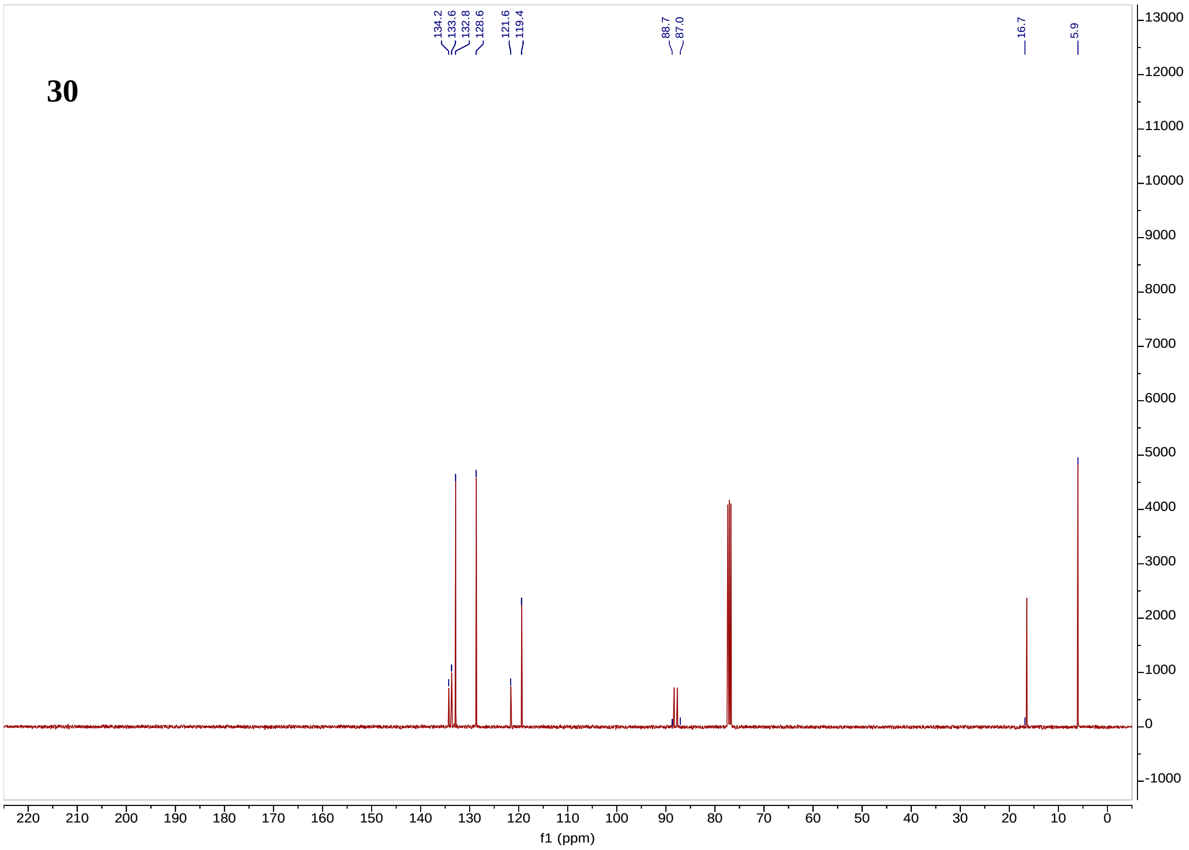

134.2 133.6 132.8 128.6 121.6 119.4
88.7 87.0
16.7
5.9
30
f1 (ppm)

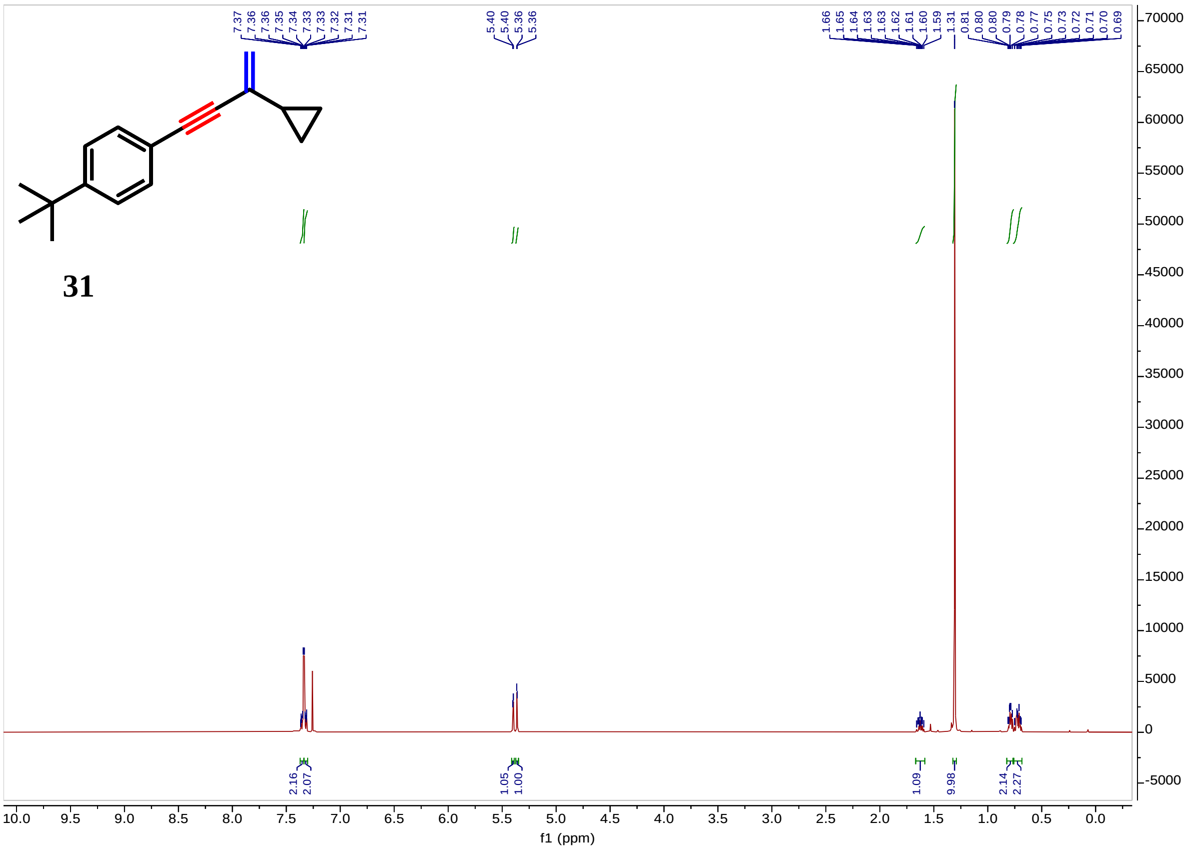
31
f1 (ppm)

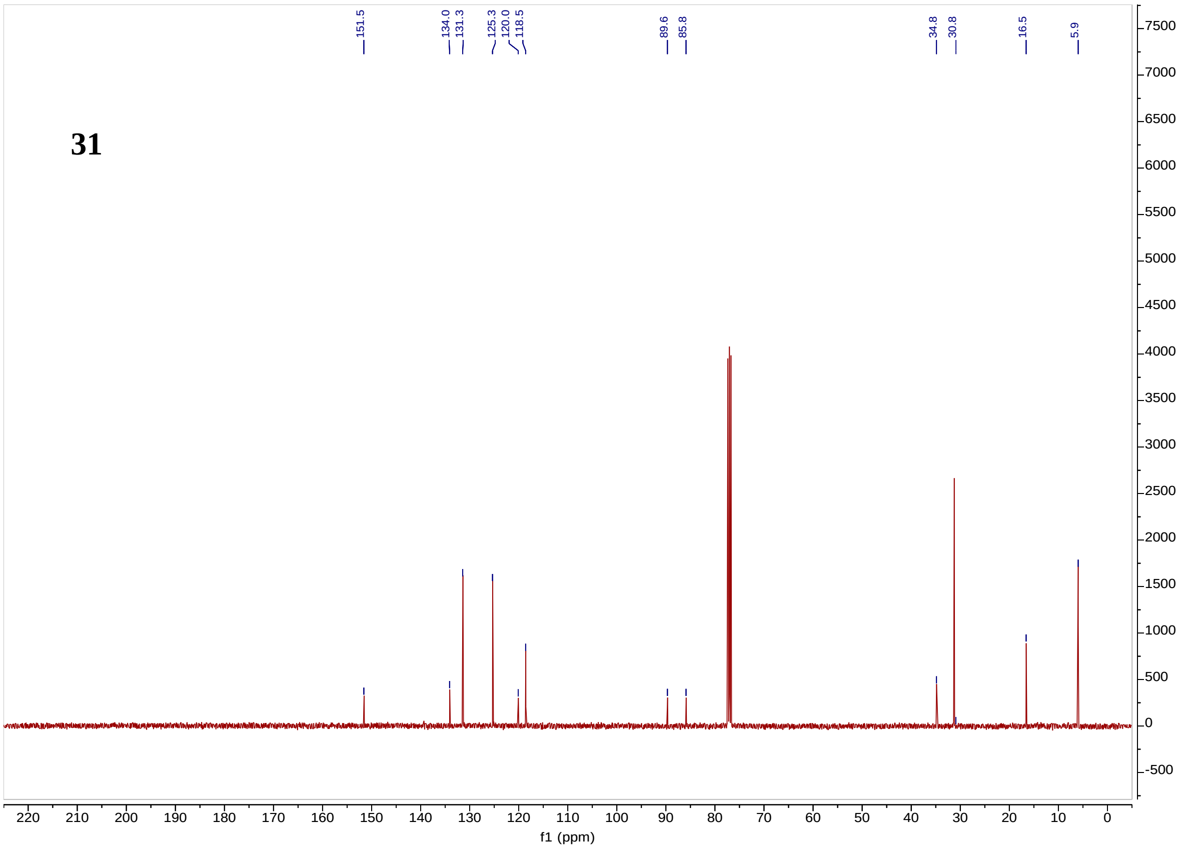
31
f1 (ppm)

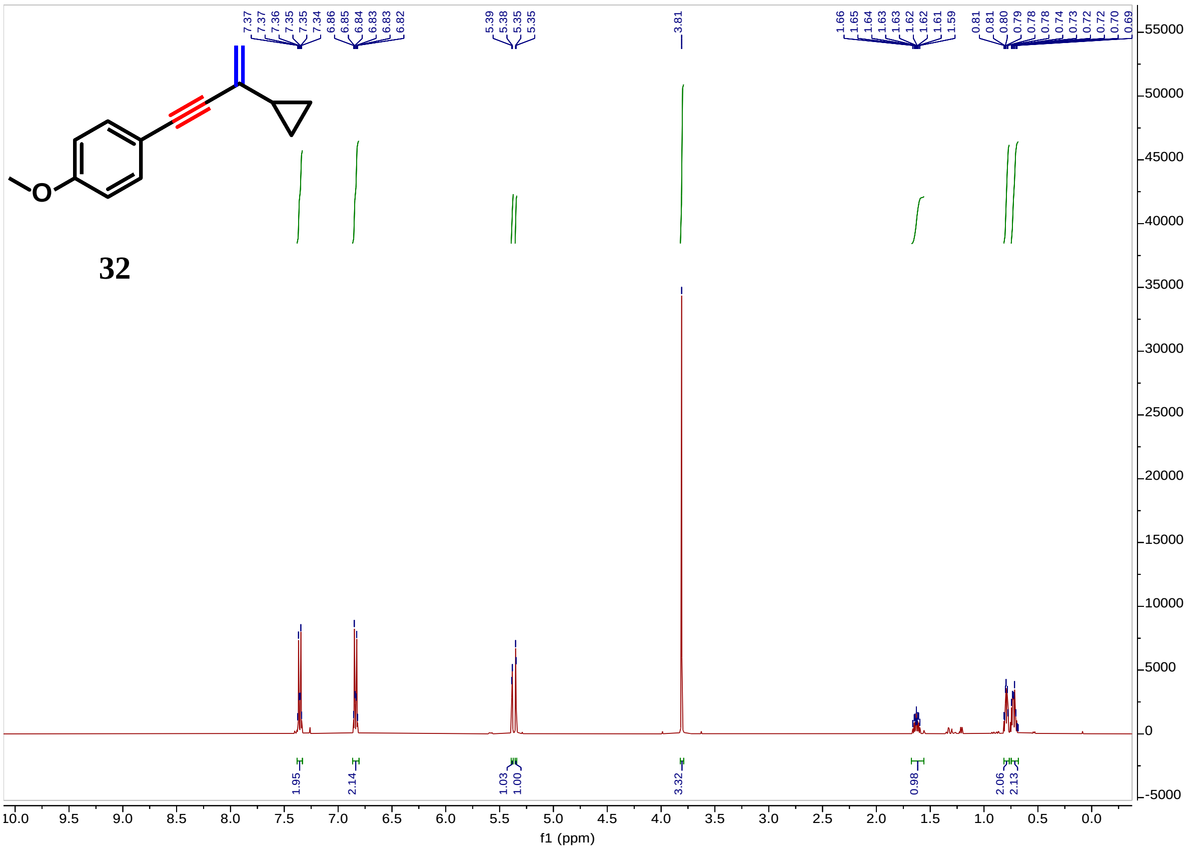
32
7.37
7.37
7.36
7.35
7.35
7.34
6.86
6.85
6.84
6.83
6.83
6.82
5.39
5.38
5.35
5.35
3.81
1.66
1.65
1.64
1.63
1.63
1.62
1.62
1.61
1.59
0.81
0.81
0.80
0.79
0.78
0.78
0.74
0.73
0.72
0.72
0.70
0.69
1.95
2.14
1.03
1.00
3.32
0.98
2.06
2.13
f1 (ppm)

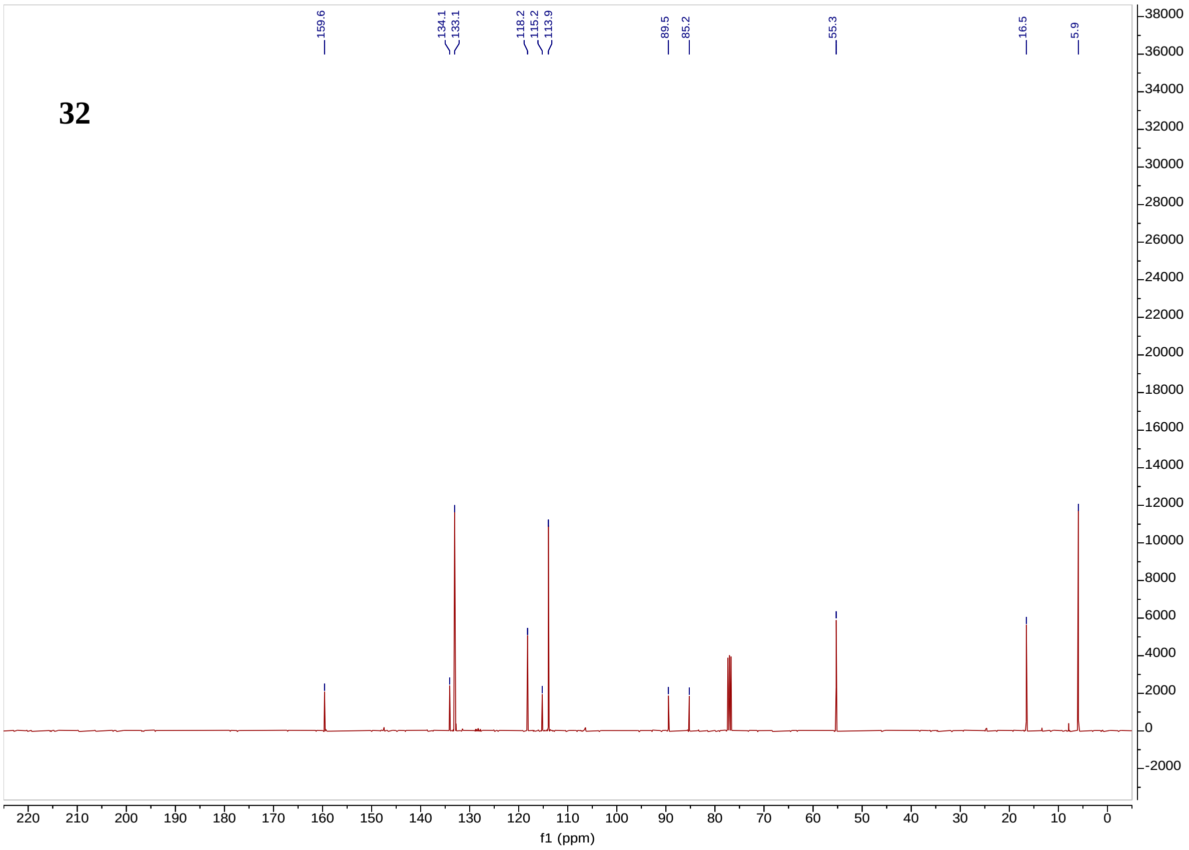
32
159.6
134.1
133.1
118.2
115.2
113.9
89.5
85.2
55.3
16.5
5.9
f1 (ppm)

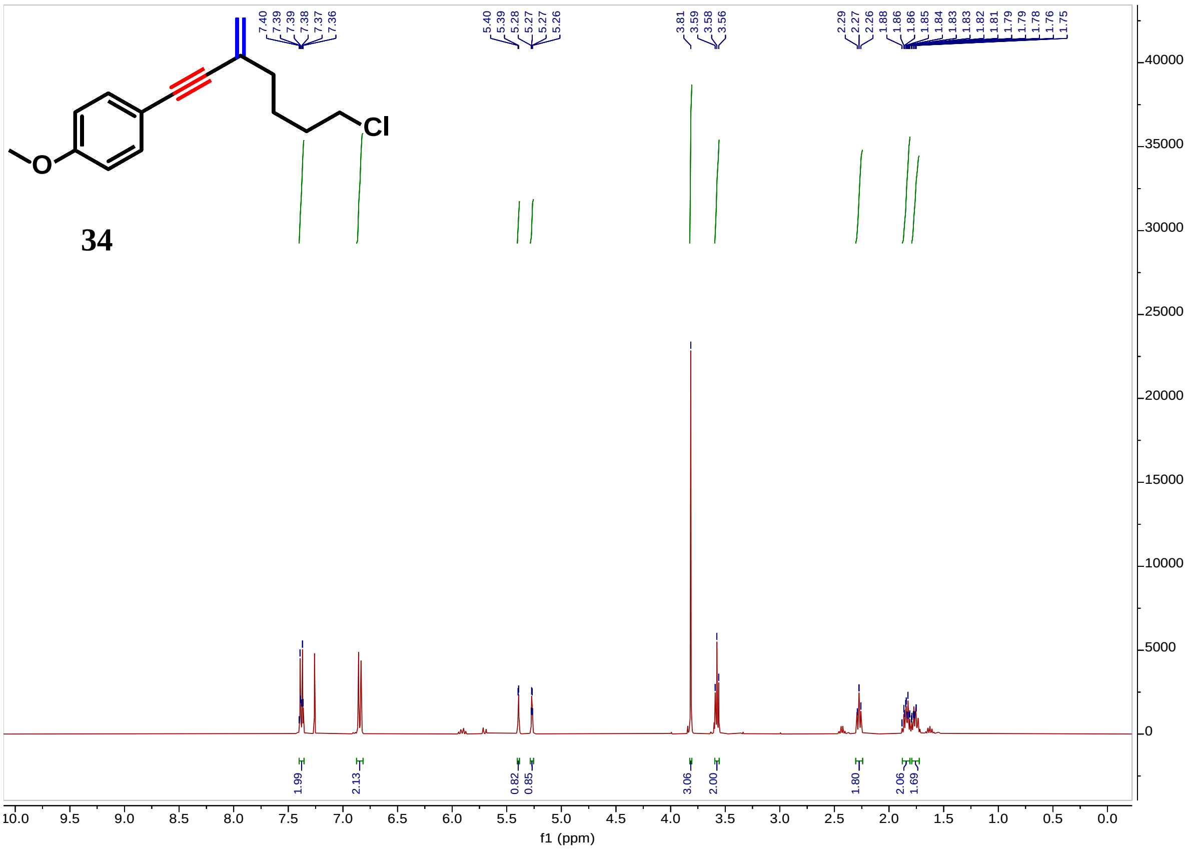

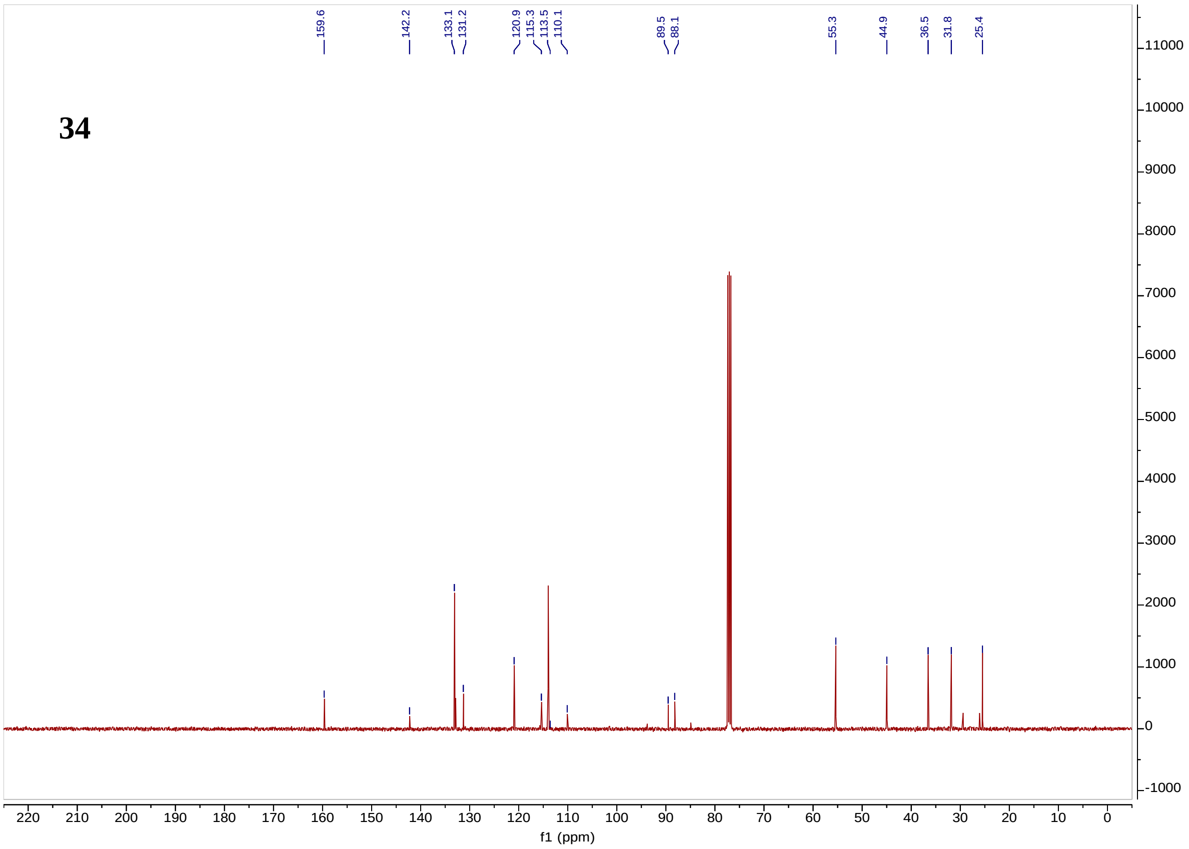

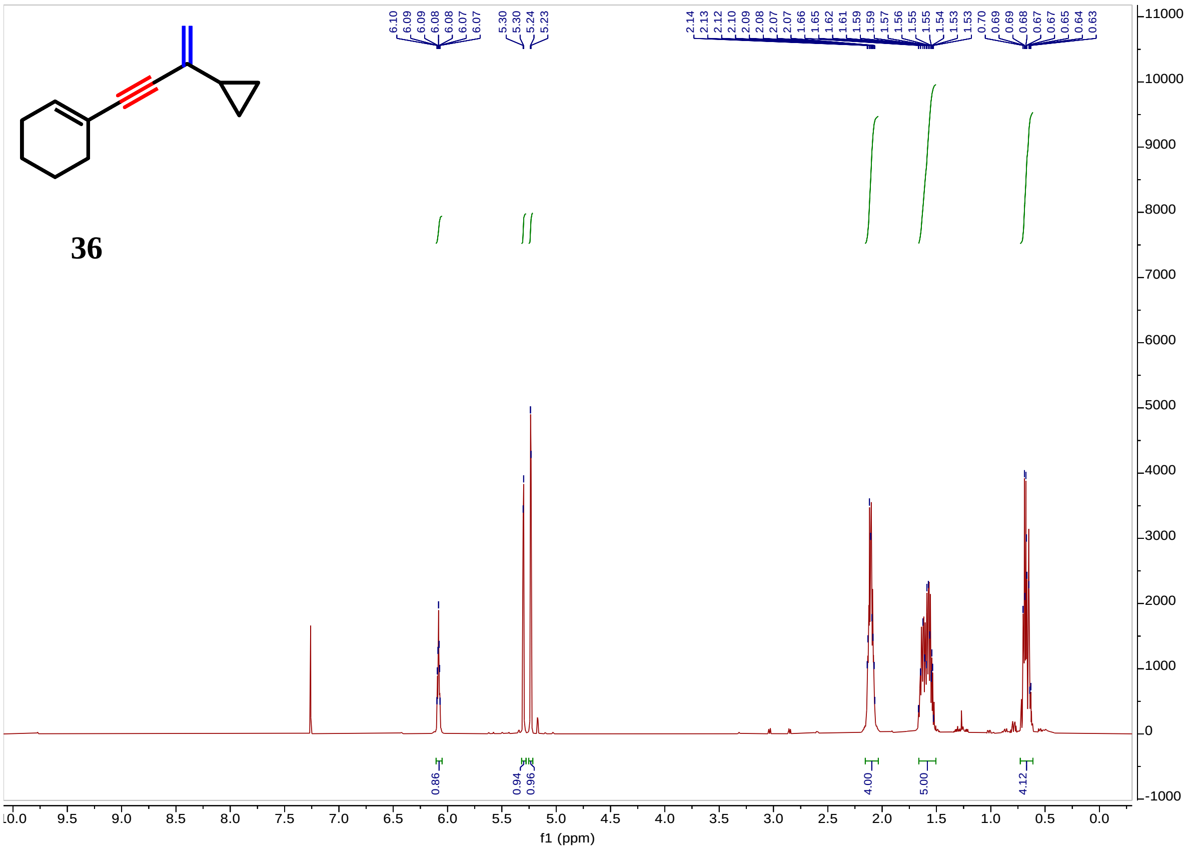
36
f1 (ppm)

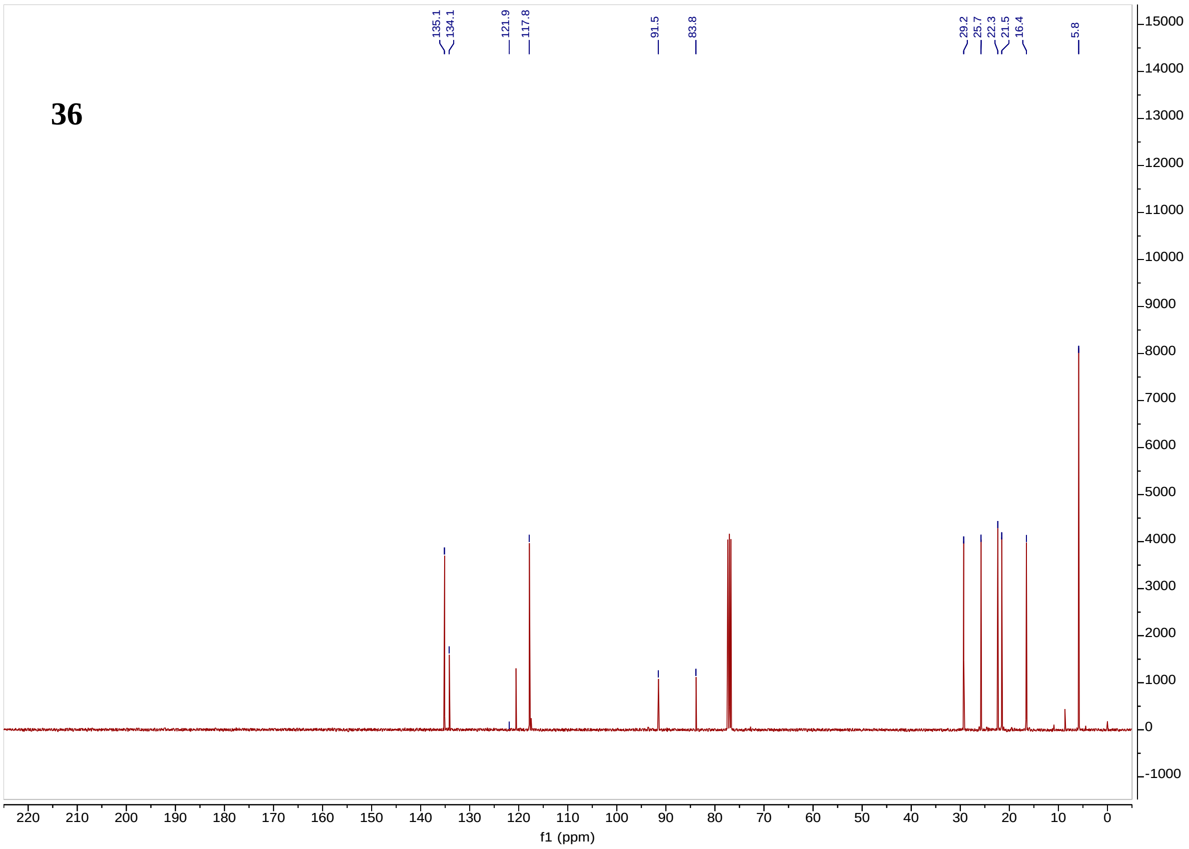
36
f1 (ppm)

7. References

(1) Weber, S.; Stöger, B.; Veiros, L. F.; Kirchner, K. Rethinking Basic Concepts - Hydrogenation of Alkenes Catalyzed by Bench-Stable Alkyl Mn(I) Complexes. *ACS Catal.* **2019**, *9*, 9715-9720.

(2) Kraihanzel, C. M., Maples, P.K. Structures of Acyl- and Methylmanganese Carbonyl Complexes of Chelating Diphosphines. *J. Organometal. Chem.* **1969**, *20*, 269-272.

(3) Garduño, J.A.; Arévalo, A.; Flores-Alamo, M.; García, J.J. Mn(I) organometallics containing the $^{i}Pr_2P(CH_2)_2P^{i}Pr_2$ ligand for the catalytic hydration of aromatic nitriles. *Catal. Sci. Technol.* **2018**, *8*, 2606-2016.

(4) Garduño, J. A.; García, J. J. Non-Pincer Mn(I) Organometallics for the Selective Catalytic Hydrogenation of Nitriles to Primary Amines. *ACS Catal.* **2018**, *9*, 392-401.

(5) Kuninobu, Y.; Ueda, H.; Kawata, A.; Takai, K. Rearrangement of Indene Skeletons under Mild Conditions. *J. Org. Chem.* **2007**,*72*, 6749-6752.

(6) Kalinowski, H.-O.; Berger, S.; Braun, S. (Eds) In *^{13}C-NMR-Spektroskopie*, Georg Thieme Verlag: Stuttgart, Germany, 1984; pp 498.

(7) Gaussian 09, Revision **A.01**, Frisch, M. J.; Trucks, G. W.; Schlegel, H. B.; Scuseria, G. E.; Robb, M. A.; Cheeseman, J. R.; Scalmani, G.; Barone, V.; Mennucci, B.; Petersson, G. A.; Nakatsuji, H.; Caricato, M.; Li, X.; Hratchian, H. P.; Izmaylov, A. F.; Bloino, J.; Zheng, G.; Sonnenberg, J. L.; Hada, M.; Ehara, M.; Toyota, K.; Fukuda, R.; Hasegawa, J.; Ishida, M.; Nakajima, T.; Honda, Y.; Kitao, O.; Nakai, H.; Vreven, T.; Montgomery, Jr., J. A.; Peralta, J. E.; Ogliaro, F.; Bearpark, M.; Heyd, J. J.; Brothers, E.; Kudin, K. N.; Staroverov, V. N.; Kobayashi, R.; Normand, J.; Raghavachari, K.; Rendell, A.; Burant, J. C.; Iyengar, S. S.; Tomasi, J.; Cossi, M.; Rega, N.; Millam, J. M.; Klene, M.; Knox, J. E.; Cross, J. B.; Bakken, V.; Adamo, C.; Jaramillo, J.; Gomperts, R.; Stratmann, R. E.; Yazyev, O.; Austin, A. J.; Cammi, R.; Pomelli, C.; Ochterski, J. W.; Martin, R. L.; Morokuma, K.; Zakrzewski, V. G.; Voth, G. A.; Salvador, P.; Dannenberg, J. J.; Dapprich, S.; Daniels, A. D.; Farkas, Ö.; Foresman, J. B.; Ortiz, J. V.; Cioslowski, J.; Fox, D. J. Gaussian, Inc., Wallingford CT, 2009.

(8) a) Haeusermann, U.; Dolg, M.; Stoll, H.; Preuss, H.; Schwerdtfeger, P.; Pitzer, R. M. Accuracy of energy-adjusted quasirelativistic ab initio pseudopotentials *Mol. Phys.* **1993**, *78*, 1211-1224. b) Kuechle, W.; Dolg, M.; Stoll, H.; Preuss, H. Energy-adjusted pseudopotentials for the actinides. Parameter sets and test calculations for thorium and

thorium monoxide *J. Chem. Phys.* **1994**, *100*, 7535-7542. c) Leininger, T.; Nicklass, A.; Stoll, H.; Dolg, M.; Schwerdtfeger, P. The accuracy of the pseudopotential approximation. II. A comparison of various core sizes for indium pseudopotentials in calculations for spectroscopic constants of InH, InF, and InCl *J. Chem. Phys.* **1996**, *105*, 1052-1059.

(9) a) Ditchfield, R.; Hehre, W. J.; Pople, J. A. Self-Consistent Molecular-Orbital Methods. IX. An Extended Gaussian-Type Basis for Molecular-Orbital Studies of Organic Molecules *J. Chem. Phys.* **1971**, *54*, 724-728. b) Hehre, W. J.; Ditchfield, R.; Pople, J. A. Self-Consistent Molecular Orbital Methods. 12. Further extensions of Gaussian-type basis sets for use in molecular-orbital studies of organic-molecules *J. Chem. Phys.* **1972**, *56*, 2257-2261. c) Hariharan, P. C.; Pople, J. A. Accuracy of AH equilibrium geometries by single determinant molecular-orbital theory *Mol. Phys.* **1974**, *27*, 209-214. d) Gordon, M. S. The isomers of silacyclopropane *Chem. Phys. Lett.* **1980**, *76*, 163-168. e) Hariharan, P. C.; Pople, J. A. Influence of polarization functions on molecular-orbital hydrogenation energies *Theor. Chim. Acta* **1973**, *28*, 213-222.

(10) Hehre, W. J., Radom, L., Schleyer, P. v.R. & Pople, J. A. *Ab Initio Molecular Orbital Theory*, John Wiley & Sons, NY, 1986.

(11) Parr, R. G. & Yang, W. *Density Functional Theory of Atoms and Molecules*; Oxford University Press: New York, 1989.

(12) a) Perdew, J. P.; Burke, K.; Ernzerhof, M. Generalized Gradient Approximation Made Simple *Phys. Rev. Lett.* **1996**, *77*, 3865-3868; b) Perdew, J. P.; Burke, K.; Ernzerhof, M. Generalized Gradient Approximation Made Simple *Phys. Rev. Lett.* **1997**, *78*, 1396-1396. c) Perdew, J. P. Density-functional approximation for the correlation energy of the inhomogeneous electron gas *Phys. Rev. B* **1986**, *33*, 8822-8824.

(13) a) Peng, C.; Ayala, P. Y.; Schlegel, H. B.; Frisch, M. J. Using redundant internal coordinates to optimize equilibrium geometries and transition states *J. Comp. Chem.* **1996**, *17*, 49-56. b) Peng, C.; Schlegel, H. B. Combining Synchronous Transit and Quasi-Newton Methods for Finding Transition States *Israel J. Chem.* **1993**, *33*, 449-454.

(14) Gorgas, N.; Alves, L.G.; Stöger, B.; Martins, A.M.; Veiros, L.F., Kirchner, K. Stable, Yet Highly Reactive Nonclassical Iron(II) Polyhydride Pincer Complexes: *Z*-Selective Dimerization and Hydroboration of Terminal Alkynes. *J. Am. Chem Soc.* **2017**, *139*, 8130 – 8133.

(15) Brar, A.; Mummadi, S.; Unruh, D.K.; Krempner C. Verkade Base in FLP Chemistry– From Stoichiometric C–H Bond Cleavage to the Catalytic Dimerization of Alkynes. *Organometallics* **2020**, *39*, 4307 – 4311.

(16) Xu, D.; Sun, Q.; Quan, Z.; Wang, X.; Sun, W. Cobalt-Catalyzed Dimerization and Homocoupling of Terminal Alkynes. *Asian J. Org. Chem.* **2018**, *7,* 155 – 159.

(17) Rubio-Pérez, L.; Azpíroz, R.; Di Guiseppe, A., Polo, V.; Castarlenas, R.; Pérez-Torrente, Oro, L.A. Pyridine-Enhanced Head-to-Tail Dimerization of Terminal Alkynes by a Rhodium–N-Heterocyclic-Carbene Catalyst. *Chem. Eur. J.* **2013**, *19*, 15304 – 15314.

(18) Chen, T.; Guo, C.; Goto, M.; Han, L.-B. A Brønsted acid-catalyzed generation of palladium complexes: efficient head-to-tail dimerization of alkynes. *Chem. Commun.* **2013**, *68*, 7498-7500.

(19) Chen, J.-F.; Li, C. Cobalt-Catalyzed gem-Cross-Dimerization of Terminal Alkynes. *ACS Catal.* **2020**, *10*, 3881 – 3889.

(20) Liu, C.-H.; Zhuang, Z.; Bose, S.; Yu, Z.-X. $Fe_2(CO)_9$-mediated [5+1] cycloaddition of vinylcyclopropanes and CO for the synthesis of α,β-cyclohexenones. *Tetrahydron* **2016**, *72*, 2752 – 2755.
